# Beyond Takai's Olefination Reagent: Persistent Dehalogenation Emerges in a Chromium(III)‐μ_3_‐Methylidyne Complex

**DOI:** 10.1002/anie.202106608

**Published:** 2021-08-01

**Authors:** Simon Trzmiel, Jan Langmann, Daniel Werner, Cäcilia Maichle‐Mössmer, Wolfgang Scherer, Reiner Anwander

**Affiliations:** ^1^ Institut für Anorganische Chemie Eberhard-Karls-Universität Tübingen Auf der Morgenstelle 18 72076 Tübingen Germany; ^2^ Institut für Physik Universität Augsburg Universitätsstr. 1 86159 Augsburg Germany

**Keywords:** chromium, cylopentadienyl, magnetism, methylidyne, olefination

## Abstract

Reaction of CHI_3_ with six equivalents of CrCl_2_ in THF at low temperatures affords [Cr_3_Cl_3_(μ_2_‐Cl)_3_(μ_3_‐CH)(thf)_6_] as the first isolable high‐yield Cr^III^ μ_3_‐methylidyne complex. Substitution of the terminal chlorido ligands via salt metathesis with alkali‐metal cyclopentadienides generates isostructural half‐sandwich chromium(III)‐μ_3_‐methylidynes [Cp^R^_3_Cr_3_(μ_2_‐Cl)_3_(μ_3_‐CH)] (Cp^R^=C_5_H_5_, C_5_Me_5_, C_5_H_4_SiMe_3_). Side and decomposition products of the Cl/Cp^R^ exchange reactions were identified and structurally characterized for [Cr_4_(μ_2_‐Cl)_4_(μ_2_‐I)_2_(μ_4_‐O)(thf)_4_] and [(η^5^‐C_5_H_4_SiMe_3_)CrCl(μ_2_‐Cl)_2_Li(thf)_2_]. The Cl/Cp^R^ exchange drastically changed the ambient‐temperature effective magnetic moment *μ*_eff_ from 9.30/9.11 *μ*_B_ (solution/solid) to 3.63/4.32 *μ*_B_ (Cp^R^=C_5_Me_5_). Reactions of [Cr_3_Cl_3_(μ_2_‐Cl)_3_(μ_3_‐CH)(thf)_6_] with aldehydes and ketones produce intricate mixtures of species through oxy/methylidyne exchange, which were partially identified as radical recombination products through GC/MS analysis and ^1^H NMR spectroscopy.

## Graphical abstract

The methylidyne complex [Cr^III^_3_Cl_3_(μ‐Cl)_3_(μ_3_‐CH)(thf)_6_] displays the ultimate product of the CrCl_2_/CHI_3_ redox reaction, significantly affecting carbonylic group transformation; the trimetallic compound also exhibits unusual magnetic behavior and provides ready access to half‐sandwich clusters.

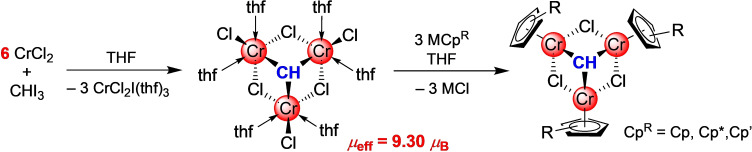

## Introduction

Carbyne or alkylidyne moieties display archetypal ligands in organo(transition)metal chemistry.^[1]^ In particular, alkylidyne complexes of the high‐oxidation‐state heavier group 6 metals molybdenum and tungsten emerged as eminent alkyne metathesis catalysts.^[2]^ Such discrete complexes feature multiply bonded terminal moieties of the type M≡CR (M=Mo, W) and have been studied comprehensively.[^3^, ^4^] On the other hand, derivatives of the first‐row homologue chromium are very rare. While molecules [X_3_Cr≡CH] (X=F, Cl) have been observed in an argon matrix (8 K),^[5]^ heteroatom‐substituted carbyne derivatives such as [(C_5_Me_5_)Cr(≡CN*i*Pr_2_)(CN*t*Bu)_3_][PF_6_]_2_ ^[6]^ and the trimetallic cluster [Cp_3_Cr_3_(μ_2_‐Cl)_3_(μ_3_‐CH)] (**A**)^[7]^ display crystalline compounds.^[7]^ Purple trivalent **A** has remained the only structurally characterized methylidyne complex of chromium.[^7^, ^8^] On the other hand, the M_3_(μ_3_‐CH) entity exhibits a common structural motif detected throughout *d*‐transition metal chemistry (Ti, Fe, Co, Ru, Re and Os).^[9]^ Prominent examples of the μ_3_‐alkylidyne compound class are tricobalt nonacarbonyl clusters, which were investigated comprehensively by Seyferth et al.^[10]^ Methylidyne complexes structurally related to **A** comprise [{Cp*Ti(μ_2_‐O)}_3_(μ_3_‐CH)]^[11]^ and [Cp*_3_Mo_3_(μ_2_‐O)_2_(μ_2_‐CH_2_)(μ_3_‐CH)]^[12]^ the reactivity of which has been investigated as well.

**Scheme 1:**
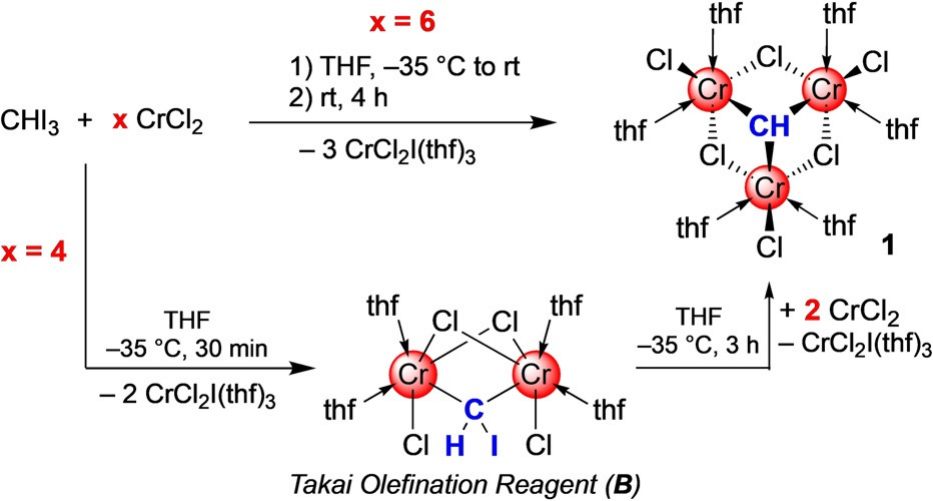
Synthesis of methylidyne complex **1**, directly (upper path) or via the Takai olefination reagent **B** (lower path).

Complex **A** has been obtained by thermal treatment (60 °C) of [CpCr(CH_3_)(μ_2_‐Cl)]_2_ via multiple abstraction of hydrogen from a methyl ligand,^[13]^ while its reactivity was not commented on.^[7]^ Dehalogenation of organic halides features another viable pathway to alkylidyne/methylidyne complexes.^[14]^ Both pathways can proceed via intermediate alkylidene/methylidene species. For chromium, well‐defined alkylidene species are just as rare as alkylidyne complexes.[^15^, ^16^] The most prominent chromium alkylidene complex is Takai's olefination/cyclopropanation reagent [Cr_2_Cl_2_(μ_2_‐Cl)_2_(μ_2_‐CHI)(thf)_4_] (**B**, shown in Scheme 1).^[16]^ The active reagent is routinely generated in situ applying the dehalogenation protocol, with CrCl_2_ and CHX_3_ (X=Cl, Br, I) as the main components in varying ratios.^[17]^ Recently, we succeeded in determining the solid‐state structure of the Takai haloalkylidene complex **B**,^[18]^ only confirming the connectivity originally proposed by Takai. By taking a closer look at the formation of the Takai olefination reagent, we have now uncovered the chromium(III) methylidyne species [Cr_3_Cl_3_(μ_2_‐Cl)_3_(μ_3_‐CH)(thf)_6_] (**1**) as the ultimate product of the CrCl_2_/CHI_3_ reaction. Interestingly, complex **1** engages in selective halogenido exchange reactions, preserving the M_3_(μ_3_‐CH) entity. Preliminary conversions of aldehydes or ketones revealed reaction pathways involving radical intermediates.

## Results and Discussion

### Formation of Methylidyne Complex [Cr_3_Cl_3_(μ_2_‐Cl)_3_(μ_3_‐CH)(thf)_6_] (1) in the Reaction of Chromium(II) Chloride with Iodoform

**Figure 1:**
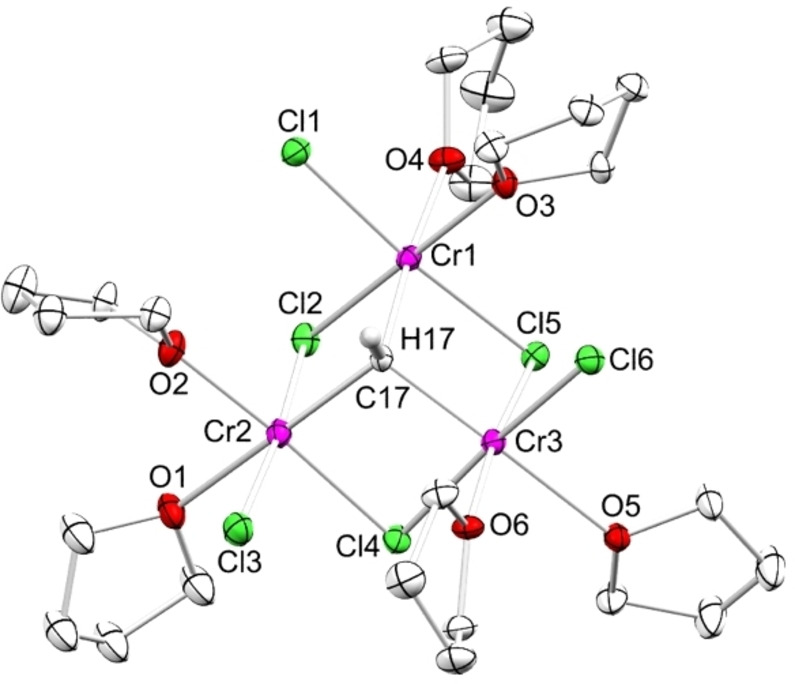
Crystal structure of **1**, ellipsoids shown at 50 % probability, THF lattice solvent and hydrogen atoms are omitted for clarity. Selected interatomic distances/angles are listed in the Supporting Information (ESI, Figure S17).^[32]^

The Takai olefination reagent is routinely generated in situ via a 3:1 mixture of CrCl_2_ and CHX_3_ (Scheme 1). The original report also mentioned the use of a 4:1 ratio in case of bromoform which did not significantly affect the yield and *E*/*Z* ratio of the alkenyl halide product.^[17]^ Therefore we pondered about whether use of excessive CrCl_2_ would affect, if at all, the formation of Takai reagent **B**. Surprisingly, the reaction of CrCl_2_ with CHI_3_ in a 6:1 molar ratio at −35 °C in THF afforded the red methylidyne complex [Cr_3_Cl_3_(μ_2_‐Cl)_3_(μ_3_‐CH)(thf)_6_] (**1**) in up to 70 % yield (Scheme 1, Figure 1) along with the precipitation of three equivalents of CrCl_2_I(thf)_3_.

Compound **1** is also accessible via **B** and addition of another two equivalents of CrCl_2_ (Scheme 1). Crystallization from the THF solution at −35 °C yielded compound **1** as a microcrystalline solid. Repeated crystallization increased the overall yield, but to the expense of co‐crystallizing CrCl_2_I(thf)_3._ Crystallization from less concentrated solutions gave red plates suitable for X‐ray diffraction (XRD) analysis. The crystal structure of **1** shows the known tetrahedral M_3_(μ_3_‐CH) structural motif, with three chromium atoms forming a nearly equilateral triangle (Figure 1). Three μ_2_‐bridging chlorido ligands complement the cluster core, resembling a truncated cube. One terminal chlorido and two THF molecules each complete the slightly distorted octahedral coordination of the Cr^III^ atoms.^[19]^

The Cr−(μ_3_‐CH) distances in **1** of 2.018(3)/2.019(3)/2.022(3) Å appear slightly longer than those in Theopold‘s compound [Cp_3_Cr_3_(μ_2_‐Cl)_3_(μ_3_‐CH)] (**A**; 1.935(10) and 1.949(14) Å), as are the bridging Cr−Cl distances (2.3328(7) to 2.4186(7) Å versus 2.348(4) to 2.360(4) Å).^[7]^ The average Cr—Cr distance of 3.167 Å is also considerably longer than in **A** (2.82 Å), which has been referred to as an unusually short contact (range for Cr−Cr single bonds: 2.65–2.97 Å).^[7]^ Correspondingly, both the Cr‐Cl‐Cr and Cr‐C‐Cr angles are more flat in **1** (81.88(2)–82.96(2)°; 102.72(12)–103.66(12)°) than in **A** (73.0(1) and 73.9(1)°; 92.4(6) and 93.8(5)°).

**Figure 2:**
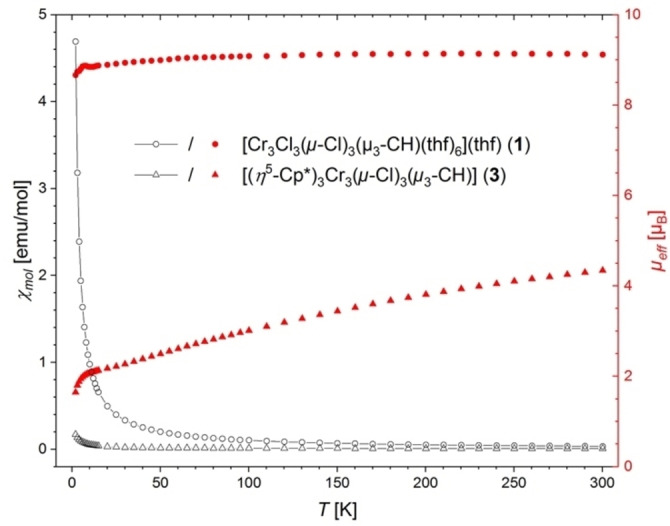
Temperature‐dependent molar magnetic susceptibility *χ*_mol_(*T*) (black open symbols; left ordinate) and effective magnetic moment *μ*_eff_(*T*) (red filled symbols; right ordinate) as obtained by SQUID magnetic measurements on crystalline powders of **1** and **3** in applied fields *H*=3 kOe and 10 kOe, respectively. The *χ*_mol_(*T*) data were corrected for diamagnetic contributions (**1**: −4.243×10^−4^ emu mol^−1^; **3**: −3.071×10^−4^ emu mol^−1^; calculated from Pascal's constants),^[23]^ and a spin‐only *g* factor of 2.0 was assumed in the calculation of *μ*_eff_(*T*). Note, that **1** contains an additional THF solvent molecule per formula unit in the crystal packing.

The ^1^H NMR spectroscopic investigation of **1** in [D_8_]THF did not reveal any signal for the μ_3_‐CH proton in the range of −500 to 500 ppm, presumably caused by paramagnetic broadening.^[20]^ Also, any distinct μ_3_‐CH vibration band was not detectable by IR‐spectroscopy (ESI, Figure S26). The effective magnetic moment of **1** in dissolved and solid form was determined by the Evans method^[21]^ and SQUID magnetic measurements, respectively. Both methods consistently point to ferro‐ or ferrimagnetic coupling between the individual Cr^III^ centers already at ambient temperature. The derived values of *μ*_eff_ (Evans method: 9.30 *μ*_B_; SQUID: 9.11 *μ*_B_) are significantly larger than those expected for three uncoupled Cr^III^ centers (6.71 *μ*_B_). Notably, the effective moment of solid **1** is nearly temperature independent down to 2 K (Figure 2, Figure S30). A fit of the field‐dependent molar magnetization *M*_mol_(H) at 2 K with a Brillouin function (the Landé *g*‐factor was assumed to be 2.0) yields a spin quantum number of S=4.45(4) which is in line with a S=9/2 ground state (Figure S32). A similar large *μ*_eff_ value of 9.61 was found for the related chromium chlorocarbyne complex [Cr_3_Cl_3_(μ_2_‐Cl)_3_(μ_3_‐CCl)(thf)_6_] assuming an S=9/2 ground state.^[8]^ We note, that also the tetranuclear Cr^III^/Cr^II^ complex [Cp^R^_4_Cr_4_(μ_2_‐H)_5_(μ_3_‐H)_2_] (Cp^R^=η^5^‐tetramethyl‐ethyl‐cyclopentadienyl) displays a temperature‐independent high *μ*_eff_ of 8.1 *μ*_B_ with S=3.4(2) which is due to intramolecular ferrimagnetic couplings and in line with a S=7/2 ground state.^[22]^

**Figure 3:**
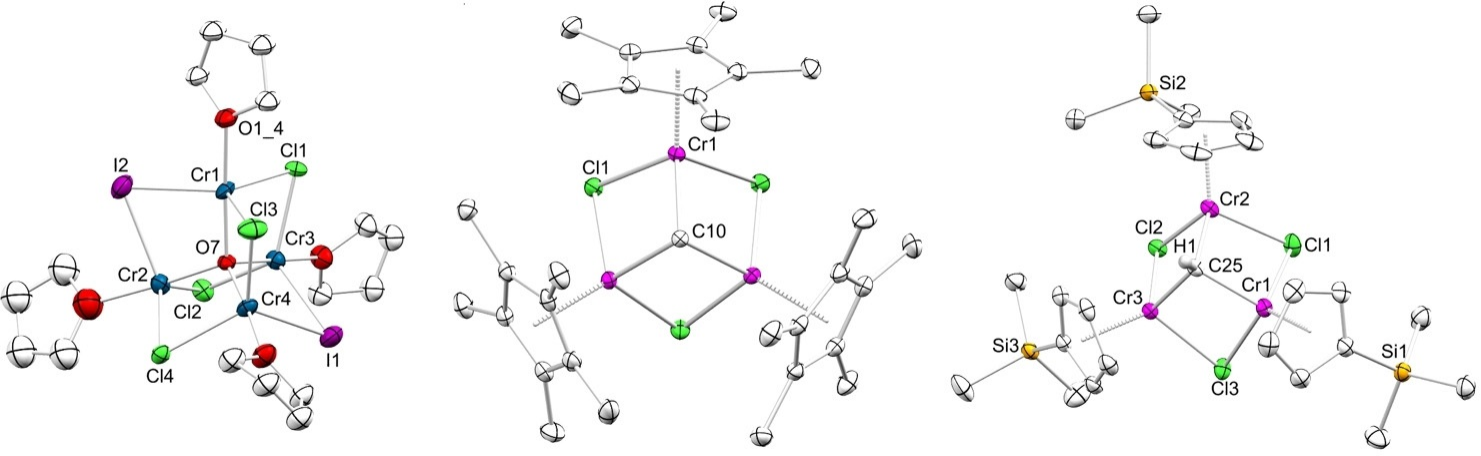
Connectivity of [Cr_4_(μ_2_‐Cl)_4_(μ_2_‐I)_2_(μ_4_‐O)(thf)_4_] (**2 a**, left), ellipsoids shown at 30 % probability, lattice solvent and hydrogen atoms are omitted for clarity. Crystal structures of [(η^5^‐Cp*)_3_Cr_3_(μ_2_‐Cl)_3_(μ_3_‐CH)] (**3**, middle) and [(η^5^‐Cp′)_3_Cr_3_(μ_2_‐Cl)_3_(μ_3_‐CH)] (**6**, right), ellipsoids shown at 50 % probability, lattice solvent and hydrogen atoms (except for the methylidyne hydrogen atom in **6**) are omitted for clarity. Selected interatomic distances/angles are listed in the Supporting Information (Figures S18/S20/S23).^[32]^

Compound **1** was found to be infinitely stable in the solid state, while high purity samples showed minor decomposition in THF at −35 °C over several weeks. Thermal decomposition of **1** in THF occurred rapidly above 40 °C (as indicated by a gradual color change from red to yellow). Similarly, progressive decomposition of **1** was observed in non‐coordinating solvents like toluene as indicated by the formation of a precipitate as well as decoloration. Utilization of high‐purity reactants is crucial for the successful synthesis of **1**, as water‐containing solvents or oxygen‐containing impurities of iodoform or CrCl_2_ (99.99 % trace metal basis, anhydrous CrCl_2_) led to partially inseparable decomposition/side products, as evidenced for the serendipitous identification of [Cr_4_(μ_2_‐Cl)_4_(μ_2_‐I)_2_(μ_4_‐O)(thf)_4_] (**2 a**) and mixed‐valent [Cr_4_Cl(μ_2_‐Cl)_4_(μ_2_‐I)_2_(μ_4_‐O)(thp)_4_] (**2 b**, thp=tetrahydropyran) by XRD analysis. As complexes **2** were obtained in minute amounts and the crystals were of poor quality, the crystal structures represent only connectivities (Figures 3/S18/S19). The molecular structure of **2 a** is isostructural to [Cr_4_(μ_2_‐Cl)_6_(μ_4_‐O)(thf)_4_] reported by Cotton et al. with two iodido ligands instead of chloridos.^[24]^ The core of complexes **2** features a [M_4_O]^6+^ entity with the central oxygen tetrahedrally coordinated by the metal atoms. Each 5‐coordinate chromium(II) engages further in two chlorido and one iodido bridge, and the coordination of a THF molecule. The single 6‐coordinate chromium(III) in **2 b** is additionally coordinated by a terminal chlorido ligand. Compounds **2** probably formed at an early stage of the reaction, most likely due to solvent water impurities. However, neither **2a** nor **2b** could be reproduced by the admittance of deliberate amounts of water to the reaction mixture.

### Formation of Half‐Sandwich Methylidyne Complexes [Cp^R^_3_Cr_3_(μ_2_‐Cl)_3_(μ_3_‐CH)(thf)_6_] via Selective Cl/Cp^R^ Exchange

**Scheme 2:**
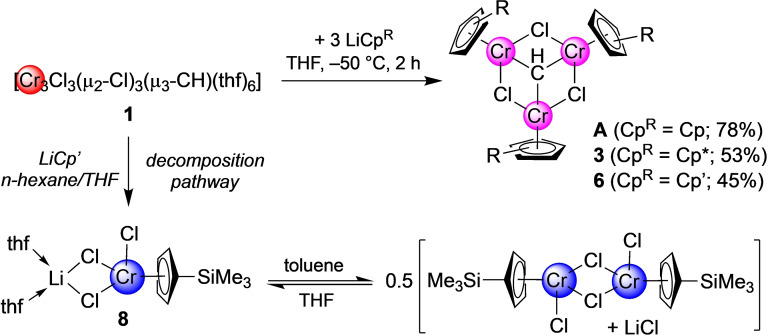
Synthesis of trimetallic half‐sandwich methylidyne complexes; representation of the suggested equilibrium of ate complex **8** in solution.

With compound **1** accessible in decent yields, we targeted the selective exchangeability of the terminal chlorido ligands. As the cyclopentadienyl ligand proved a stabilizing ligand for the M_3_(μ_3_‐CH) entity in general, and specifically for compound **A**, we probed the reactivity of **1** toward NaCp (Cp=C_5_H_5_) and the substituted cyclopentadienides LiCp* and LiCp′ (Cp*=C_5_Me_5_, Cp′=[C_5_H_4_(SiMe_3_)] (Scheme 2).

Reaction of **1** with three equivalents of NaCp in THF at −50 °C yielded a clear dark purple solution. Crystallization from *n*‐pentane gave purple needles of [Cp_3_Cr_3_(μ_2_‐Cl)_3_(μ_3_‐CH)] (**A**) in good yield (78 %). Lower yields at elevated temperatures and absence of metathesis salt most likely result from the instability of **1** dissolved in THF and its partial decomposition during the reaction, through unknown reduction pathways. Moreover, chromocene [Cp_2_Cr] could be identified as a major impurity by ^1^H NMR spectroscopy ([D_8_]THF, *δ*=319.4 ppm) being separable by sublimation at 40 °C. Other Cr^II^ and Cr^III^ species present in reaction mixtures, even at −50 °C, were not assignable by NMR spectroscopy (distinct signals in the range 10 to 50 ppm), but could be removed by crystallization. The ^1^H NMR spectrum of **A** measured in [D_8_]THF at ambient temperature shows a broad singlet at *δ*=30.27 ppm (in CDCl_3_ at *δ*=31.05 ppm), in agreement with the literature.^[7]^

The 3‐equivalent reaction of **1** with LiCp* in THF at −50 °C led to an instant color change from dark red to dark green. Crystallization from concentrated toluene/*n*‐hexane mixtures gave dark green crystals of [(η^5^‐Cp*)_3_Cr_3_(μ_2_‐Cl)_3_(μ_3_‐CH)] (**3**) featuring a structural motif similar to **1** (Figure 1) and **A**. Compound **3** crystallizes in the trigonal space group *R3* and displays a local symmetry of *C_3_* with Cr—Cr distances of 2.9103(5) Å, slightly longer than in **A**. The Cr−Cl distances of 2.3416(5) to 2.3615(5) Å as well as the Cr‐C‐Cr angles involving the central μ_3_‐CH moiety (92.71(11)°) match those in **A**. The ^1^H NMR spectrum of crystalline **3** in [D_8_]THF at ambient temperature shows a slightly broadened singlet at *δ*=−5.8 ppm assignable to C_5_(C*H*_3_)_5_. The ambient‐temperature magnetic moment drastically changed upon Cl/Cp^R^ exchange as evidenced by the Evans method in solution (*μ*_eff_=3.63 *μ*_B_) and in the solid state by SQUID measurements (*μ*_eff_=4.32 *μ*_B_). These values are in accordance with the results obtained for dissolved **A** (Evans method: *μ*_eff_=3.55 *μ*_B_)^[7]^ and substantially below the effective magnetic moment expected in case of three uncoupled Cr^III^ centers (*μ*_eff_=6.71 *μ*_B_). A possible explanation may be the establishment of antiferromagnetic interactions causing the observed gradual decrease of *μ*_eff_ for solid **3** upon cooling (Figure 2, Figures S31/S33).^[7]^ A similar temperature‐dependent decrease of the effective magnetic moment upon cooling has been observed earlier in the related complex **A** and considered as an evidence for antiferromagnetic couplings between the chromium ions.^[7]^ A further analogy to **A** is that reaction mixtures of **3** show a multitude of paramagnetically shifted proton signals, due to partial reduction and decomposition of complex **1**. Identified side products comprise Cp*_2_Cr (*δ*=−6.2 ppm, [D_8_]THF)^[25]^ and [Cp*CrCl_2_]_2_ (*δ*=−71.5 ppm, CDCl_3_).^[26]^ Overall, the synthesis of such half‐sandwich complexes is extremely sensitive toward change of reaction conditions and choice of precursor. While switching the solvent from THF to toluene led to the isolation of trivalent [Cp*CrCl_2_(thf)] (**4**), probing the direct synthesis of **3** from [Cp*Cr(μ_2_‐Cl)]_2_/CHI_3_ gave only partial halogenido exchange in [(Cp*Cr)_2_(μ_2_‐Cl)(μ_2_‐I)] (**5**) (synthesis details and crystal structures, see Supporting Information).

Salt metathesis of **1** with three equivalents of LiCp′ in THF at −50 °C gave a dark red/violet solution. After several extraction steps, crystallization from *n*‐hexane yielded dark purple needles of [(η^5^‐Cp′)_3_Cr_3_(μ_2_‐Cl)_3_(μ_3_‐CH)] (**6**). The crystal structure of **6** is isostructural to **A** and **3** (Figure 3), with similar Cr−Cl distances of 2.3243(4) Å to 2.3519(4) Å. Not unexpectedly, the Cr—Cr distances of 2.8192(3) Å to 2.8363(3) Å match those in **A**. The ^1^H NMR spectrum of **6** recorded in [D_8_]THF at ambient temperature shows two broadened signals at *δ*=35.35 and 30.39 ppm for the aromatic protons of the Cp′ ligands, and one sharp singlet at *δ*=0.49 ppm for the SiC*H*_3_ protons (*μ*_eff_=2.70 *μ*_B_). Again, the ^1^H NMR spectrum of the reaction mixture of **6** shows numerous other signals. To prove similar reaction/decomposition behavior as found for **A** and **3**, chromocene Cp′_2_Cr (**7**) was synthesized independently from CrCl_2_ and LiCp′. Crystallization of **7** from *n*‐hexane produced orange crystals suitable for an X‐ray diffraction study (Figure S24). The ^1^H NMR spectrum of **7** measured in [D_8_]THF at ambient temperature displays signals at *δ*=322.33 ppm, 249.42 ppm, and −3.32 ppm (Figure S5). Half‐sandwich ate complex [(η^5^‐Cp′)CrCl(μ_2_‐Cl)_2_Li(thf)_2_] (**8**) could be crystallized as another side product from reactions in THF. The crystal structure of deep blue **8** proved the existence of an intramolecular ate complex (Figure S25). Compound **8** provides further evidence for the equilibrium theory proposed by Rojas et al. (Scheme 2) and explains the virtually non‐existent (non‐separable) amount of metathesis salt in the reaction mixtures of **1** and compounds MCp^R^.^[27]^ Nearly identical solubilities of the side products clearly counteract the isolation of these compounds. In general, the proneness of **1** to reduction (and the formation of Cp^R^_2_Cr^II^) can be minimized by performing the reactions at low temperatures in less polar solvents. In THF, the reactions proceeded with minor impurities only at −50 °C, while in *n*‐hexane and *n*‐pentane acceptable results were obtained at −35 °C. Toluene is unsuitable as a solvent, since decomposition of **1** was significant within minutes, even at low temperatures.

### Reactivity of Methylidyne Complex 1 toward Aldehydes and Ketones

**Scheme 3:**
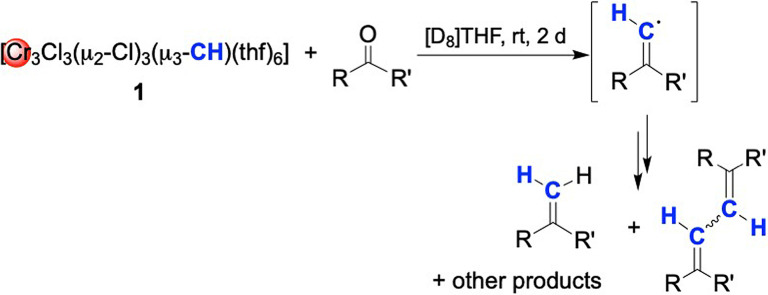
Reactions of **1** with aldehydes and ketones.

The Takai and Takai‐Utimoto olefination reagents engage in (*E*)‐selective olefinations of aldehydes, with high functional group tolerance.[^28^, ^29^] Later, reagent extensions involved the formation of (heteroatom‐)substituted cyclopropane products.^[30]^ It was of interest how the methylidyne complex **1** would affect such olefination reactions. Direct NMR‐scale reactivity studies turned out difficult to interpret because of paramagnetic shifting and broadening. However, filtration of the reaction mixtures over aluminum oxide facilitated the observation of organic products via ^1^H NMR spectroscopy. The conversions of benzaldehyde and pivaldehyde with **1** in [D_8_]THF were complete after 1 h at ambient temperature.^[31]^ During this period, the mixtures changed color from deep red to turbid green brown, leading to a multitude of products as detected by GC/MS analysis (see Figures S34 to S43). Most of these compounds are suggested to be formed by radical recombination, involving transient olefinic radical, as a result from methylidyne/oxy exchange (Scheme 3). 1D and 2D NMR spectroscopies could not resolve the observed overlapping signals of the product mixtures (Figures S6 to S12). For example, the benzaldehyde reaction revealed the formation of styrene as the only component identifiable by ^1^H NMR spectroscopy. Striking was the observation of trace amounts of (2‐iodoethenyl)‐benzene by GC/MS analysis. As the synthesis of **1** produces a substantial amount of the iodinated side product CrCl_2_I(thf)_3_ (approximate solubility of 1 mg mL^−1^ in THF at 17 °C), product contamination with iodine seems inevitable. ICP (Inductively Coupled Plasma) analysis of recrystallized samples of compound **1** indicated a persistent iodine content of roughly 3.3 %.

Other causes for the iodine contamination could be the presence of decomposition product **2** or non‐reacted Takai reagent [Cr_2_Cl_2_(μ_2_‐Cl)_2_(μ_2_‐CHI)(thf)_4_] (**B**), which are both easily soluble in THF and hence difficult to separate via crystallization.

Compound **1** did not show any reactivity toward alkynes HC≡CSiMe_3_, HC≡CPh and PhC≡CPh, neither alkyne metathesis nor insertion/addition‐type reactions. The latter investigations were carried out in [D_8_]THF and monitored by ^1^H NMR spectroscopy over several hours, also by heating to the decomposition temperature of **1**. Finally, the reactivity of [(η^5^‐Cp*)_3_Cr_3_(μ_2_‐Cl)_3_(μ_3_‐CH)] (**3**) toward benzaldehyde or benzophenone was examined under similar conditions, but the ^1^H NMR spectra were inconclusive and only indicated decomposition of the methylidyne complex. Further research is needed to elucidate the reactivity of the organometallic compounds.

## Conclusion

The chromium(III) μ_3_‐methylidyne complex [Cr_3_Cl_3_(μ_2_‐Cl)_3_(μ_3_‐CH)(thf)_6_] features the ultimate C‐X cleavage product in the dehalogenation sequence of haloforms CHX_3_(here: CrCl_2_/CHI_3_ mixture). The decent yields of the methylidyne complex enabled a series of reactivity studies. The terminal chlorido ligands can be selectively displaced via salt metathesis with alkali‐metal cyclopentadienides to afford rare examples of half‐sandwich chromium(III) methylidynes, [(η^5^‐Cp^R^)_3_Cr_3_(μ_2_‐Cl)_3_(μ_3_‐CH)]. Despite the paramagnetic nature of Cr^III^, these compounds exhibit only slightly broadened signals in the ^1^H NMR spectra, facilitating the observation of in situ derivatizations. Treatment of [Cr_3_Cl_3_(μ_2_‐Cl)_3_(μ_3_‐CH)(thf)_6_] with ketones and aldehydes led to olefination, entailing the formation of various products probably formed by radical recombination. The methylidyne complexes under study do not promote alkyne metathesis reactions or insertions/additions with acetylenes, but display exceptional magnetic behavior. Finally, our study underlines the importance of complying with correct CrCl_2_/haloform ratios for efficient olefination reactions.

## Conflict of interest

The authors declare no conflict of interest.

## Supporting information

As a service to our authors and readers, this journal provides supporting information supplied by the authors. Such materials are peer reviewed and may be re‐organized for online delivery, but are not copy‐edited or typeset. Technical support issues arising from supporting information (other than missing files) should be addressed to the authors.

Supporting Information

## References

[1] C. Elschenbroich, Organometallics, Wiley-VCH, Weinheim, 2006.

[2a] R. R. Schrock, Chem. Rev. 2002, 102, 145–179; 11782131 10.1021/cr0103726

[2b] A. Fürstner, P. W. Davies, Chem. Commun. 2005, 2307–2320; 10.1039/b419143a 15877114

[2c] A. Fürstner, Angew. Chem. Int. Ed. 2013, 52, 2794–2819; 10.1002/anie.201204513 23355479

[2d] Y. Jin, Q. Wang, P. Taynton, W. Zhang, Acc. Chem. Res. 2014, 47, 1575–1586; 24739018 10.1021/ar500037v

[2e] H. Ehrhorn, M. Tamm, Chem. Eur. J. 2019, 25, 3190–3208. 30346054 10.1002/chem.201804511

[3] For examples, see:

[3a] J. H. Wengrovius, J. Sancho, R. R. Schrock, J. Am. Chem. Soc. 1981, 103, 3932–3934;

[3b] J. C. Peters, A. L. Odom, C. C. Cummins, Chem. Commun. 1997, 118, 1995–1996;

[3c] A. Fürstner, C. Mathes, C. W. Lehmann, J. Am. Chem. Soc. 1999, 121, 9453–9454;

[3d] S. Beer, C. G. Hrib, P. G. Jones, K. Brandhorst, J. Grunenberg, M. Tamm, Angew. Chem. Int. Ed. 2007, 46, 8890–8894; 10.1002/anie.200703184 17935104

[3e] E. F. van der Eide, W. E. Piers, M. Parvez, R. McDonald, Inorg. Chem. 2007, 46, 14–21; 17198408 10.1021/ic0611342

[3f] R. R. Thompson, M. E. Rotella, P. Du, X. Zhou, F. R. Fronczek, R. Kumar, O. Gutierrez, S. Lee, Organometallics 2019, 38, 4054–4059;

[3g] J. Hillenbrand, M. Leutzsch, A. Fürstner, Angew. Chem. Int. Ed. 2019, 58, 15690–15696; 10.1002/anie.201908571 PMC6856820 31449713

[3h] A. Haack, J. Hillenbrand, M. Leutzsch, M. van Gastel, F. Neese, A. Fürstner, J. Am. Chem. Soc. 2021, 143, 5643–5648. 33826335 10.1021/jacs.1c01404 PMC8154524

[4] For a terminally bonded niobium(V) methylidyne, see: T. Kurogi, P. J. Carroll, D. J. Mindiola, J. Am. Chem. Soc. 2016, 138, 4306–4307. 26977892 10.1021/jacs.6b00830

[5] J. T. Lyon, H.-G. Cho, L. Andrews, Organometallics 2007, 26, 6373–6387.

[6] A. C. Filippou, B. Lungwitz, K. M. A. Wanninger, E. Herdtweck, Angew. Chem. Int. Ed. Engl. 1995, 34, 924–927;

[7] D. S. Richeson, S. W. Hsu, N. H. Fredd, G. van Duyne, K. H. Theopold, J. Am. Chem. Soc. 1986, 108, 8273–8274.

[8] Parallel to our work, the isostructural trinuclear chromium chlorocarbyne complex [Cr_3_Cl_3_(μ-Cl)_3_(μ_3_-CCl)(thf)_6_] has been obtained from carbon tetrachloride and ca. 7 equivalents of CrCl_2_, along with compound **1** as a decomposition/hydrolysis product: T. Kurogi, K. Irifune, T. Enoki, K. Takai, Chem. Commun. 2021, 57, 5199–5202. 10.1039/d1cc01514a 33908491

[9a] A. G. Orpen, T. F. Koetzle, Acta Crystallogr. Sect. B 1984, 40, 606–612;

[9b] T. Kakigano, H. Suzuki, M. Igarashi, Y. Morooka, Organometallics 1990, 9, 2192–2194;

[9c] D. Lentz, H. Michael, Chem. Ber. 1990, 123, 1481–1483;

[9d] U. Flörke, H.-J. Haupt, Z. Kristallogr. Cryst. Mater. 1993, 204, 292–294;

[9e] V. Moberg, M. A. Mottalib, D. Sauer, Y. Poplavskaya, D. C. Craig, S. B. Colbran, A. J. Deeming, E. Nordlander, Dalton Trans. 2008, 2442–2453; 18461200 10.1039/b717698h

[9f] M. González-Moreiras, M. Mena, A. Pérez-Redondo, C. Yélamos, Chem. Eur. J. 2017, 23, 3558–3561; 28152207 10.1002/chem.201700152

[9g] Y. Takahashi, Y. Nakajima, H. Suzuki, T. Takao, Organometallics 2017, 36, 3539–3552.

[10] D. Seyferth, J. E. Hallgren, P. L. K. Hun, J. Organomet. Chem. 1973, 50, 265–275.

[11] M. Gómez-Pantoja, P. Gómez-Sal, A. Hernán-Gómez, A. Martín, M. Mena, C. Santamaría, Inorg. Chem. 2012, 51, 8964–8972. 22845551 10.1021/ic301057u

[12] O. I. Guzyr, J. Prust, H. W. Roesky, C. Lehmann, M. Teichert, F. Cimpoesu, Organometallics 2000, 19, 1549–1555.

[13] Thermal degradation of [Cp*TiMe_3_] gave [Cp*Ti(μ_3_-CH)]_4_: R. Andrés, P. Gómez-Sal, E. de Jesús, A. Martin, M. Mena, C. Yélamos, Angew. Chem. Int. Ed. Engl. 1997, 36, 115–117;

[14a] W. T. Dent, L. A. Duncanson, R. G. Guy, W. H. B. Reed, B. L. Shaw, Proc. Chem. Soc. 1961, 169;

[14b] G. Bor, L. Markó, B. Markó, Chem. Ber. 1962, 95, 333–340;

[14c] D. Seyferth, R. J. Spohn, M. R. Churchill, K. Gold, F. R. Scholer, J. Organomet. Chem. 1970, 23, 237–255;

[14d] R. Bejot, A. He, J. R. Falck, C. Mioskowski, Angew. Chem. Int. Ed. 2007, 46, 1719–1722; 10.1002/anie.200604015 17226884

[15a] S. K. Noh, R. A. Heintz, C. Janiak, S. C. Sendlinger, K. H. Theopold, Angew. Chem. Int. Ed. Engl. 1990, 29, 775–777;

[15b] S. Hao, J.-I. Song, P. Berno, S. Gambarotta, Organometallics 1994, 13, 1326–1335;

[15c] R. A. Heintz, S. Leelasubcharoen, L. M. Liable-Sands, A. L. Rheingold, K. H. Theopold, Organometallics 1998, 17, 5477–5485;

[15d] P. Wei, D. W. Stephan, Organometallics 2003, 22, 1712–1717;

[15e] P. Wei, D. W. Stephan, Organometallics 2003, 22, 1992–1994;

[15f] S. Licciulli, K. Albahily, V. Fomitcheva, I. Korobkov, S. Gambarotta, R. Duchateau, Angew. Chem. Int. Ed. 2011, 50, 2346–2349; 10.1002/anie.201006953 21351351

[15g] P. Wu, G. P. A. Yap, K. H. Theopold, J. Am. Chem. Soc. 2018, 140, 7088–7091; 29847922 10.1021/jacs.8b04882

[15h] P. Wu, G. P. A. Yap, K. H. Theopold, Organometallics 2019, 38, 4593–4600;

[15i] P. K. R. Panyam, L. Stöhr, D. Wang, W. Frey, M. R. Buchmeiser, Eur. J. Inorg. Chem. 2020, 3673–3681;

[15j] N. Wei, D. Yang, J. Zhao, T. Mei, Y. Zhang, B. Wang, J. Qu, Organometallics 2021, 40, 1434–1442.

[16a] M. Murai, R. Taniguchi, N. Hosokawa, Y. Nishida, H. Mimachi, T. Oshiki, K. Takai, J. Am. Chem. Soc. 2017, 139, 13184–13192; 28814078 10.1021/jacs.7b07487

[16b] M. Murai, R. Taniguchi, T. Kurogi, M. Shunsuke, K. Takai, Chem. Commun. 2020, 56, 9711–9714. 10.1039/d0cc03986a 32700694

[17] K. Takai, K. Nitta, K. Utimoto, J. Am. Chem. Soc. 1986, 108, 7408–7410. 10.1021/ja00279a068 22175376

[18] D. Werner, R. Anwander, J. Am. Chem. Soc. 2018, 140, 14334–14341. 30213182 10.1021/jacs.8b08739

[19] For further comparison, a mixed methyl/iodido chromium complex was obtained by oxidative addition of CH_3_I to a Cr−Cr quintuple bond: A. Noor, S. Schwarz, R. Kempe, Organometallics 2015, 34, 2122–2125.

[20a] K. H. Theopold, Acc. Chem. Res. 1990, 23, 263–270;

[20b] F. H. Köhler, C. Krüger, H. J. Zeh, Organomet. Chem. 1990, 386, C13–C15;

[20c] M. Enders, Macromol. Symp. 2006, 236, 38–47.

[21] D. F. Evans, J. Chem. Soc. 1959, 2003–2005.

[22] R. A. Heintz, T. F. Koetzle, R. L. Ostrander, A. L. Rheingold, K. H. Theopold, P. Wu, Nature 1995, 378, 359–362.

[23] G. A. Bain, J. F. Berry, J. Chem. Educ. 2008, 85, 532–536.

[24] F. A. Cotton, C. A. Murillo, I. Pascual, Inorg. Chem. 1999, 38, 2746–2749. 10.1021/ic990007l 11671004

[25] F. H. Koehler, B. Metz, W. Strauss, Inorg. Chem. 1995, 34, 4402–4413.

[26] B. Bräunlein, F. H. Köhler, W. Strauß, H. Zeh, Z. Naturforsch. B 1995, 50, 1739–1747.

[27] R. Rojas, M. Valderrama, J. Organomet. Chem. 2004, 689, 2268–2272.

[28a] L. A. Wessjohann, G. Scheid, Synthesis 1999, 1–36;

[28b] “Olefination of Carbonyl Compounds by Zinc and Chromium Reagents”: S. Matsubara, K. Oshima, in Modern Carbonyl Olefination (Ed.: T. Takeda), Wiley-VCH, Weinheim, 2003, pp. 200–222.

[29] T. Okazoe, K. Takai, K. Utimoto, J. Am. Chem. Soc. 1987, 109, 951–953.

[30a] K. Takai, S. Toshikawa, A. Inoue, R. Kokumai, J. Am. Chem. Soc. 2003, 125, 12990–12991; 14570448 10.1021/ja0373061

[30b] K. Takai, S. Toshikawa, A. Inoue, R. Kokumai, M. Hirano, J. Organomet. Chem. 2007, 692, 520–529;

[30c] M. Murai, C. Mizuta, R. Taniguchi, K. Takai, Org Lett. 2017, 19, 6104–6107; 29083928 10.1021/acs.orglett.7b02956

[30d] M. Murai, R. Taniguchi, C. Mizuta, K. Takai, Org. Lett. 2019, 21, 2668–2672. 30946593 10.1021/acs.orglett.9b00658

[31] Compound **1** proved comparatively less reactive toward ketones resulting in predominant recovery of the unreacted educts after 2 to 3 days (e.g., 88 % for benzophenone, see Figure S7; quantifications for reactions with 9-fluorenone and cyclohexanone were infeasible). For the reactions of **1** with benzophenone and 9-fluorenone, the alkylidene exchange products 1,1-diphenyl-ethylene (Figure S45) and 9-methylene-9*H*-fluorene (Figure S50) were found as the only product species identifiable by ^1^H NMR spectroscopy (Figures S7 to S10). GC/MS analysis revealed trace amounts of numerous other compounds in the product solutions, among them iodinated olefination products as well as radical recombination products.

[32] Deposition Numbers 2084204, 2084205, 2084206, 2084207, 2084208, 2084209, 2084210, 2084211 and 2084212 contain the supplementary crystallographic data for this paper. These data are provided free of charge by the joint Cambridge Crystallographic Data Centre and Fachinformationszentrum Karlsruhe Access Structures service www.ccdc.cam.ac.uk/structures.

