# Supplementary material for: Beyond Takai's Olefination Reagent: Persistent Dehalogenation Emerges in a Chromium(III)‐μ3‐Methylidyne Complex
Source: Angew Chem Int Ed Engl. 2021 Aug 1;60(36):20049–54. doi: 10.1002/anie.202106608 (PMC8456800; doi:10.1002/anie.202106608)
Supplement: Supplementary file 1 — Supporting Information [file ANIE-60-20049-s001.pdf]

Angewandte Chemie
Eine Zeitschrift der Gesellschaft Deutscher Chemiker

Supporting Information

Beyond Takai's Olefination Reagent: Persistent Dehalogenation Emerges in a Chromium(III)-μ_3-Methylidyne Complex

Simon Trzmiel, Jan Langmann, Daniel Werner, Cäcilia Maichle-Mössmer, Wolfgang Scherer, and Reiner Anwander**

anie_202106608_sm_miscellaneous_information.pdf

Table of Contents

Experimental Procedures	S3
Syntheses	S3
Reactivity Studies	S6
NMR Spectra	S7
UV/Vis Spectra	S13
Crystallographic Details	S15
Infrared Spectra	S22
SQUID Measurements	S24
Gas Chromatography – Mass Spectrometry	S28
References	S44

Experimental Procedures

General Procedures. All manipulations were performed using a glovebox (MBraun 200B; <0.1 ppm O_2, <0.1 ppm H_2O) or Schlenk line techniques under an atmosphere of purified argon in oven dried glassware. Solvents (THF, *n*-hexane, *n*-pentane and toluene) were purified over Grubbs-type columns (MBraun SPS, solvent purification system) and stored in a glovebox. THF and THP were further stored over 3 Å molecular sieve. Chromium(II) chloride was purchased from Abcr (99.99% pure, trace metal basis) and used as received. Iodoform was purchased from Sigma-Aldrich and sublimed before use. Benzaldehyde was purchased from Sigma-Aldrich, purified by distillation, and stored over pre-dried 3 Å molecular sieve. Pivalaldehyde, benzophenone, 9-fluorenone, trimethylsilylacetylene, phenylacetylene, 1,2-diphenylacetylene, *n*-butyllithium (1.6 M in *n*-hexane), 1,2,3,4,5-pentamethylcylopentadiene, and cyclohexanone were purchased from Sigma-Aldrich and used as received. LiCp* was synthesized from *n*-BuLi and HCp*. $[Cp^*CrCl]_2$ was synthesized according to a literature procedure.[1] The NMR spectra of air and moisture sensitive compounds were performed in pre-dried (over NaK alloy) benzene-d_6, chloroform-d_3, or THF-d_8, with J. Young valved NMR-spectroscopy tubes. NMR spectra were recorded on a Bruker AVII+400 (^{1}H: 400.13 MHz, ^{13}C: 100.16 MHz) at 26 °C. ^{1}H shifts are referenced to a solvent resonance and reported in parts per million (ppm) relative to tetramethylsilane.[2] ^{1}H NMR measurements were performed with a scan range of 1000 ppm (–500 – 500 ppm). Analyses of the NMR spectra were performed with Bruker TOPSPIN (version 3.6.1). The Evans method has been carried out on a Bruker AVII+400 at 298 K in THF/THF-d_8, with hexamethyldisiloxane as reference.[3] Concentrations of the complexes in THF solution ranged from 7-10 mg/mL. Additional measurements of the DC magnetic moment in solid samples were performed using the SQUID magnetometer Quantum Design MPMS-XL. The temperature dependence of the magnetic moment was determined between 2 K and 300 K in applied magnetic fields of 3 kOe or 10 kOe. Additional field-dependent data were collected between -50 kOe and 50 kOe at a temperature of 2 K. The samples were supplied in powdered crystalline form and held by gelatin capsules packed into surrounding plastic straws. All sample containers showed a minor magnetic moment in the range of 10^{-5} emu in the temperature range between 2 K and 300 K at an applied field of 10 kOe. Continuous inert conditions were ensured by sample preparation in a glovebox under argon atmosphere and subsequent transfer to the magnetometer in an air-tight transport vessel. UV-Vis measurements were performed on a PG Instruments T60 UV-Vis spectrophotometer as dilute THF solutions. Infrared spectra were recorded on a Thermo Fisher Scientific NICOLET 6700 FTIR ($\tilde{\nu}$= 4000 – 400 cm^{-1}), using a DRIFT chamber with dry KBr/sample mixtures and KBr windows. Elemental analyses (C, H, N) were performed on an Elementar vario MICRO cube. GC/MS was performed on an Agilent Q 5973 with electron impact ionization, and the obtained mass spectral data analyzed with Agilent MassHunter (version 10.0.368)[4] and compared to Main EI MS Library (mainlib)[5].

Syntheses

Synthesis of $[Cr_3Cl_3(\mu\text{-}Cl)_3(\mu_3\text{-}CH)(thf)_6]$ (1). $CrCl_2$ (500.0 mg, 4.068 mmol) was stirred in THF (5 mL) at −35 °C, giving a grey slurry. A solution of CHI_3 (267.0 mg, 0.678 mmol) was slowly added dropwise at −35 °C. Upon warming to ambient temperature over half an hour, the solution turned deep red, and orange $CrCl_2I(thf)_3$ precipitated from the solution. The mixture was stirred at ambient temperature for 4 h and then cooled to −35 °C prior to centrifugation. After filtration the solution was concentrated and stored at −35 °C, giving a red microcrystalline solid and a dark red supernatant solution. Repeating this process gave **1** as a red microcrystalline solid (387 mg, 70%). ^{1}H NMR (26 °C, 400.00 MHz, THFd_8): did not show any signals in the range of −500 to 500 ppm due to the paramagnetic behavior of the compound. χ^{mol} = 3.58x10^{-3} cm^3 mol^{-1}, μ_{eff} = 9.24 μ_B. IR (DRIFT): $\tilde{\nu}$ = 2970 (s), 2900 (s), 1455 (m), 13635 (vw), 1340 (w), 1315 (w), 1295 (w), 1245 (w), 1175 (w), 1025 (s), 1015 (s), 9201 (s), 860 (s), 680 (w) cm^{-1}. UV-vis (THF solution, λ_{max}, nm): 344, 527. Elemental analysis calcd. (%) for $C_{25}H_{49}Cl_6Cr_3O_6$ (814,35 g mol^{-1}, dry solid **1**): C 36.87, H 6.07; found: C 36.56, H 5.98.

When the supernatant reddish brown solution from the synthesis of **1** was re-crystallized, small amounts of **2a** were separable as emerald green crystal. These crystals only formed in the presence of THF which was not thoroughly dried prior to use. The same crystals could be isolated from reactions of **1** with benzaldehyde in THF. After stirring the reaction mixture 3 to 18 h at rt, the orange

solution was filtered and the precipitate discarded. The resulting green solution was concentrated and crystalline **2a** could be harvested as emerald green blocks.

In an attempt to synthesize the tetrahydropyrane (THP) derivative [$Cr_3Cl_3(\mu$-$Cl)_3(\mu_3$-CH)(thp)$_6$], mixed-valent complex **2b** could be isolated: $CrCl_2$ (200 mg, 1.627 mmol) was suspended in THP at –35 °C and CHI_3 (106.8 mg, 0.271 mmol) suspended in THP at –35 °C added. The color slowly changed from grey to orange over 1 h. The solution was warmed to ambient temperature and stirred for additional 18 h. The formed dark red suspension was filtered and the orange precipitate was discarded. The resulting dark red solution was concentrated and crystallized giving red to brown blocks of [$Cr_4Cl(\mu$-$Cl)_4(\mu$-$I)_2(\mu_4$-O)(thp)$_4$] (**2b**) after one night at –35 °C.

Synthesis of [η^5-$Cp_3Cr_3(\mu$-$Cl)_3(\mu_3$-CH)] (A). [$Cr_3Cl_3(\mu$-$Cl)_3(\mu_3$-CH)(thf)$_6$] (**1**) (184 mg, 0.226 mmol) was dissolved in THF and cooled to−50 °C. Addition of solid Na(C_5H_5) (60.0 mg, 0.681 mmol) and stirring the reaction mixture at −50 °C for 2 h gave a deep red/purple solution. The solvent was removed under reduced pressure and the solid residue extracted with *n*-pentane to separate Cp_2Cr. The dark violet residue of this extraction was redissolved in toluene. Filtration and drying of this solution gave a dark purple residue. Heating of this residue on a magnetic stirrer to 45 °C led to the sublimation of remaining Cp_2Cr. Crystallization of the dark purple solid residue from *n*-pentane through slow-evaporation at 17 °C yielded purple needles of **A** (83 mg, 78%). ^{1}H NMR (26 °C, 400.00 MHz, thf-*d$_8$*): δ = 30.25 (s, 5 H; Ar-H); elemental analysis calcd (%) for $C_{16}H_{16}Cl_3Cr_3$: C 40.83, H 3.43; found: C 39.89, H 3.63.

Synthesis of [(η^5-$C_5Me_5)_3Cr_3(\mu$-$Cl)_3(\mu_3$-CH)] (3). [$Cr_3Cl_3(\mu$-$Cl)_3(\mu_3$-CH)(thf)$_6$] (**1**) (126.5 mg, 0.155 mmol) was dissolved in THF at – 50 °C and solid Li(C_5Me_5) (66.3 mg, 0.466 mmol) was added. The mixture turned from dark red to dark green within seconds. Centrifugation, filtration, and drying of the filtrate under reduced pressure gave a dark green residue. The dark green solid was then redissolved in toluene, concentrated, and filtered. Crystallization from a toluene/*n*-hexane mixture yielded dark green crystals of **3** (56 mg, 53%). ^{1}H NMR (26 °C, 400.00 MHz, THF-*d$_8$*): δ = −5.8 (s, 15 H; Ar-CH_3). χ^{mol} = 5.53x10^{-4} cm^3 mol^{-1}, μ_{eff} = 3.63 μ_B. IR (DRIFT): $\tilde{\nu}$ = 2909 (vs), 1456 (m), 1375 (s), 1023 (m), 729 (vw), 494 (w), 436 (w) cm^{-1}. UV-vis (THF solution, λ_{max}, nm): 600. Elemental analysis calcd (%) for $C_{31}H_{46}Cl_3Cr_3$: C 54.67, H 6.81; found: C 53.80, H 6.82.

Attempt to synthesize **3** in toluene solution: [$Cr_3Cl_3(\mu$-$Cl)_3(\mu_3$-CH)(thf)$_6$] (**1**) (50.0 mg, 61.6 μmol) was suspended in toluene at –35 °C. LiCp* (26.2 mg, 184.2 μmol) was added as a solid. The suspension was stirred at –35 °C for 30 minutes, centrifuged, and filtered. The resulting dark green solution was dried in *vacuo* and the black residue extracted with Et_2O. The resulting dark blue-green solution was concentrated and filtered again. The resulting clear dark blue-green solution was dried in *vacuo* and recrystallized from THF at –35 °C over several days, giving several dark blue blocks handpicked and identified as [Cp*$CrCl_2$(thf)] (**4**) by XRD analysis. The yield was not calculated due to other impurities.

Attempt to synthesize **3** directly from [Cp*Cr(μ-Cl)]$_2$ and CHI_3: [Cp*Cr(μ-Cl)]$_2$ (29.1 mg, 65.3 μmol) was suspended in *n*-hexane and CHI_3 (4.3 mg, 10.9 μmol) was added as a suspension in *n*-hexane. The color of the solution changed to turbid green. After stirring at ambient temperature for 2 h, the solution was filtered, and the resulting dark green nearly black solution was concentrated. Crystallization of this solution at –35 °C over several days resulted in dark blue blocks of [(Cp*Cr)$_2$(μ-Cl)(μ-I)] (**5**), identified by XRD analysis. The yield could not be calculated.

Synthesis of [(η^5-$C_5H_4SiMe_3$)]$_3Cr_3(\mu$-$Cl)_3(\mu_3$-CH)] (6). [$Cr_3Cl_3(\mu$-$Cl)_3(\mu_3$-CH)(thf)$_6$] (**1**) (64.7 mg, 79 μmol) was dissolved in THF, cooled to –50 °C, and Li($C_5H_4SiMe_3$) (21.0 mg, 0.238 mmol) added as a solid. After stirring the mixture at –50 °C for 2.5 h the solvent was removed under reduced pressure giving a purple slurry. Addition of toluene and filtration gave a clear purple solution. Drying of this solution under reduced pressure and crystallization from *n*-hexane yielded dark violet needles of **4** (25 mg, 45%). ^{1}H NMR (26 °C, 400.00 MHz, thf-*d$_8$*): δ = 35.35 (s, 2 H; Ar-H), 30.39 (s, 2 H; Ar-H), 0.49 ppm (s, 9 H; $SiMe_3$). χ^{mol} = 3.06x10^{-4} cm^3 mol^{-1}, μ_{eff} = 2.70 μ_B. IR (DRIFT): $\tilde{\nu}$ = 2949 (s), 2894 (w), 1365 (w), 12456 (s), 1168 (s), 1043 (s), 902 (s), 838 (vs), 756 (m), 694 (vw), 632 (w) cm^{-1}. UV-vis (THF solution, λ_{max}, nm): 445 (sh), 564, 655 (sh). Elemental analysis calcd (%) for $C_{25}H_{40}Cl_3Cr_3Si_3$: C 43.70, H 5.87; found: C 43.92, H 5.84.

Synthesis of [(η^5-$C_5H_4SiMe_3)_2$Cr] (7). $CrCl_2$ (53.0 mg, 0.431 mmol) was suspended in THF and Li($C_5H_4SiMe_3$) (61.0 mg, 0.423 mmol, 0.98 equiv.) added as a THF solution. The grey slurry turned red over 10 minutes. After stirring at ambient temperature for 4 h, the turbid red solution was filtered, and the solvent removed under reduced pressure. The residue was extracted several times with *n*-

pentane. Crystallization from concentrated *n*-pentane yielded orange needles of **5** (58 mg, 89%). ^1H NMR (26 °C, 400.00 MHz, THF-d_8): δ = 322.32 (s, 2 H; Ar-H), 249.42 (s, 2 H; Ar-H), -3.23 ppm (s, 9 H; $SiMe_3$). IR (DRIFT): $\tilde{\nu}$ = 3087 (w), 2953 (s), 2895 (m), 1413 (w), 1246 (s), 1169 (s), 1150 (s), 1029 (m), 892 (m), 838 (s), 799 (s), 752 (s), 691 (m), 628 (m), 574 (m) cm^{-1}. UV-vis (THF solution, λ_{max}, nm): 348, 452. Elemental analysis calcd (%) for $C_{16}H_{26}CrSi_2$: C 58.85, H 8.03; found: C 58.81, H 7.71.

Attempt to synthesize **6** in *n*-hexane solution: $[Cr_3Cl_3(\mu\text{-}Cl)_3(\mu_3\text{-}CH)(thf)_6]$ (**1**) (128 mg, 0.157 mmol) was suspended in *n*-hexane and cooled to – 35 °C. LiCp' (41.5 mg, 0.472 mmol) was added as a suspension in *n*-hexane at –35 °C. The color of the suspension changed from pale red to brown. After stirring at –35 °C for 1.5 h, the suspension was centrifugated and the precipitate was discarded. The supernatant brown solution was dried in vacuo and the residue extracted with THF, giving a green solution. This green solution was crystallized yielding colorless $CrCl_2(thf)_2$ (identified by XRD analysis). The crystals were discarded, and the residue redissolved in thf yielding a dark blue solution. Crystallization of this blue solution yielded several blue crystals identified as $[(\eta^5\text{-}C_5H_4SiMe_3)CrCl(\mu\text{-}Cl)_2Li(thf)_2]$ (**8**) by XRD analysis. The yield could not be calculated due to co-crystallization of several other unidentified compounds and impurities.

Reactivity Studies

General procedure for reactions of [Cr_3Cl_3(μ-Cl)$_3$(μ$_3$-CH)(thf)$_6$] (1) with Ketones and Aldehydes.

[Cr_3Cl_3(μ-Cl)$_3$(μ$_3$-CH)(thf)$_6$] (**1**, 20.0 mg, 24.6 μmol) was dissolved in thf-*d*$_8$. Benzophenone (4.4 mg, 24.1 μmol, 1 equivalent) was added and the mixture stirred at ambient temperature for 3 days. After complete reaction, the solution was filtered through a small Al_2O_3 column and the colorless product mixture analyzed by ^{1}H NMR spectroscopy. The product mixture was then used for further product identification via GCMS analysis.

General procedure for reactions of [Cr_3Cl_3(μ-Cl)$_3$(μ$_3$-CH)(thf)$_6$] (1) with Acetylenes.

[Cr_3Cl_3(μ-Cl)$_3$(μ$_3$-CH)(thf)$_6$] (**1**, 10.0 mg, 12.3 μmol) was dissolved in thf-*d*$_8$ and transferred to a J.Young-valved NMR tube. Between 1 and 2 equivalents (12.3 to 25 μmol) of acetylene were added and the tube shaken to ensure even distribution of reactants. The NMR tube was then sealed, the mixture heated to 50 °C and the reaction monitored *via* ^{1}H NMR spectroscopy over several h.

NMR Spectra

Solvent signals are marked with *.

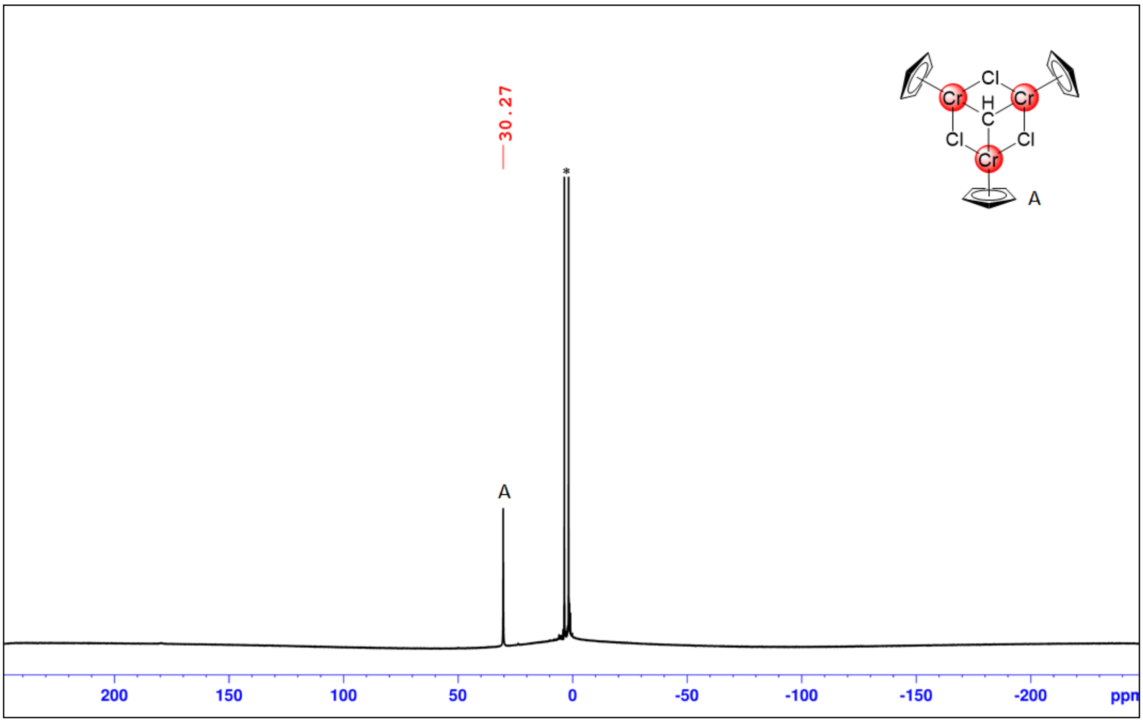

Figure S1. ^{1}H NMR spectrum (26 °C, 400.13 MHz, thf-d_8) of [(η^5-C_5H_5)Cr_3(μ_2-Cl)$_3$(μ_3-CH)] (**A**).

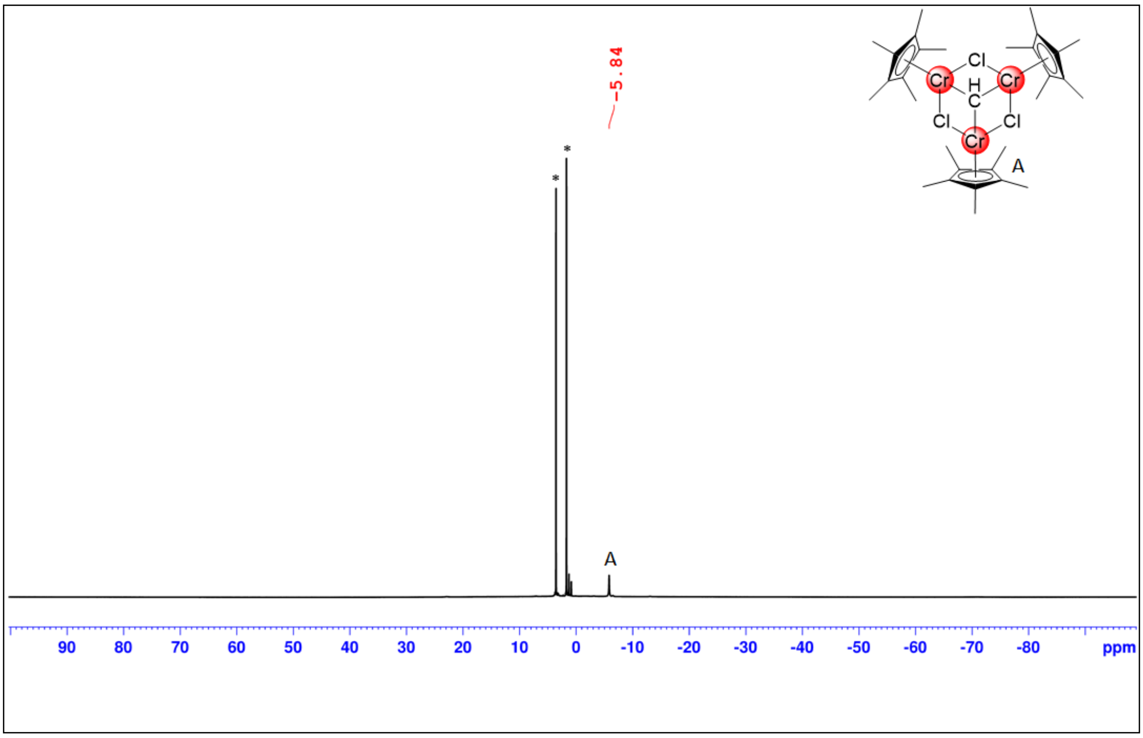

Figure S2. ^{1}H NMR spectrum (26 °C, 400.13 MHz, thf-d_8) of [(η^5-C_5Me_5)Cr_3(μ_2-Cl)$_3$(μ_3-CH)] (**3**).

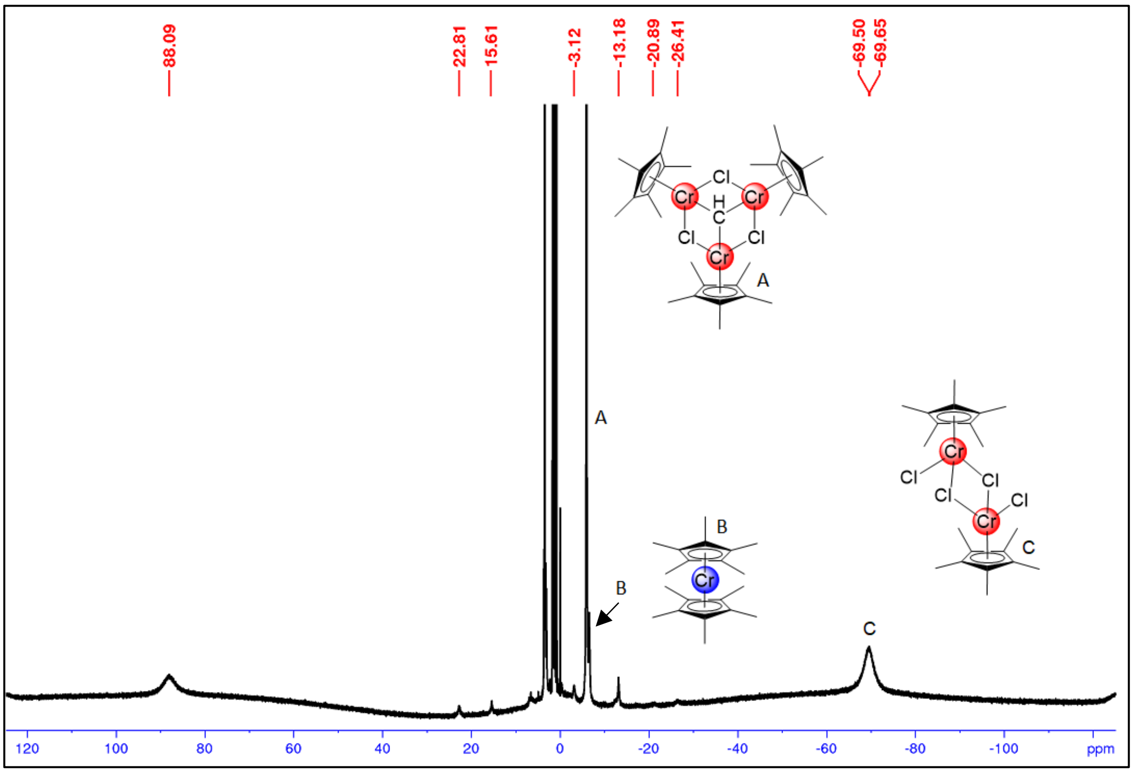

Figure S3. ^{1}H NMR spectrum (26 °C, 400.13 MHz, thf-d_8) of the reaction of **1** with 3 equivalents of LiCp* in THF at –35 °C after 1.5 h without workup. Displayed are the proposed compounds found in the mixture.

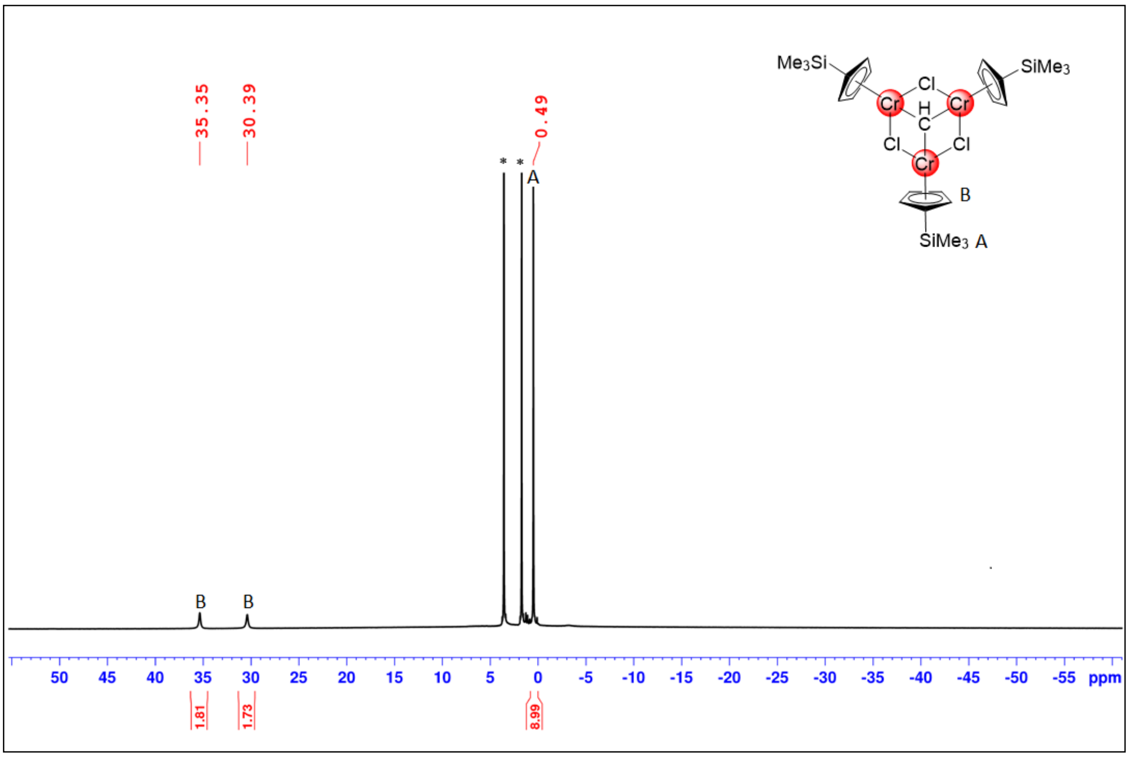

Figure S4. ^{1}H NMR spectrum (26 °C, 400.13 MHz, thf-d_8) of [(η[5]-$C_5H_4SiMe_3$)Cr_3(μ_2-Cl)$_3$(μ_3-CH)] (**6**).

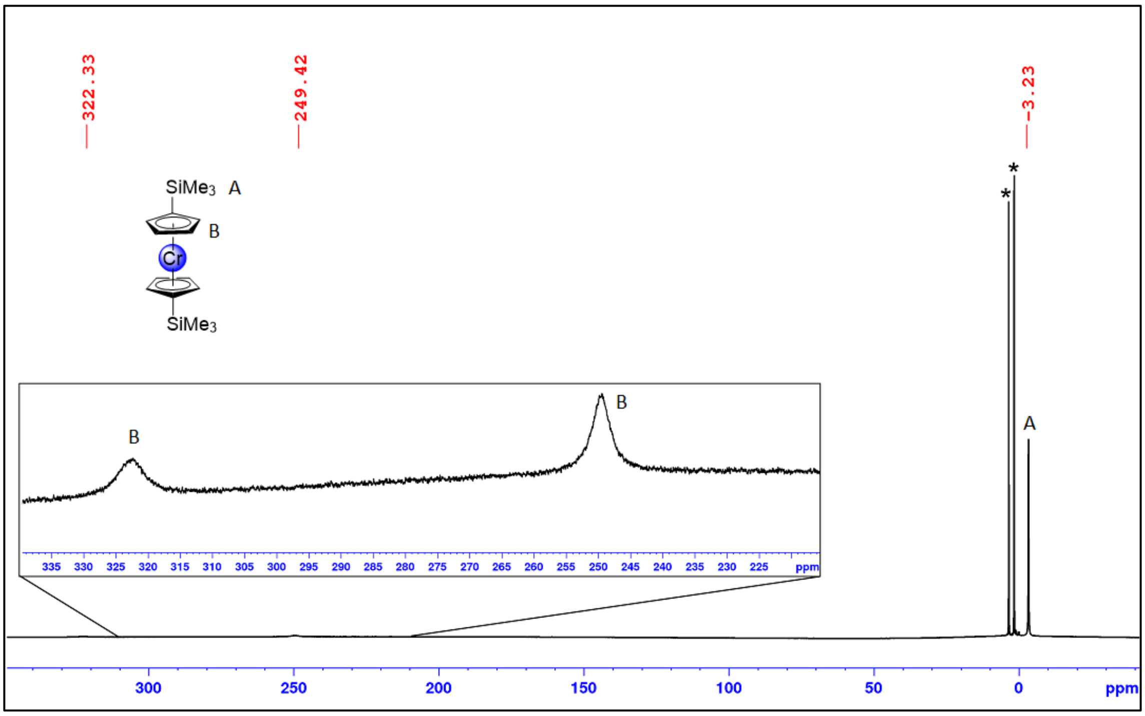

Figure S5. ^{1}H NMR spectrum (26 °C, 400.13 MHz, thf-d_8) of [(η[5]-$C_5H_4SiMe_3)_2Cr$] (**7**).

Reactions of 1 with Aldehydes and Ketones.

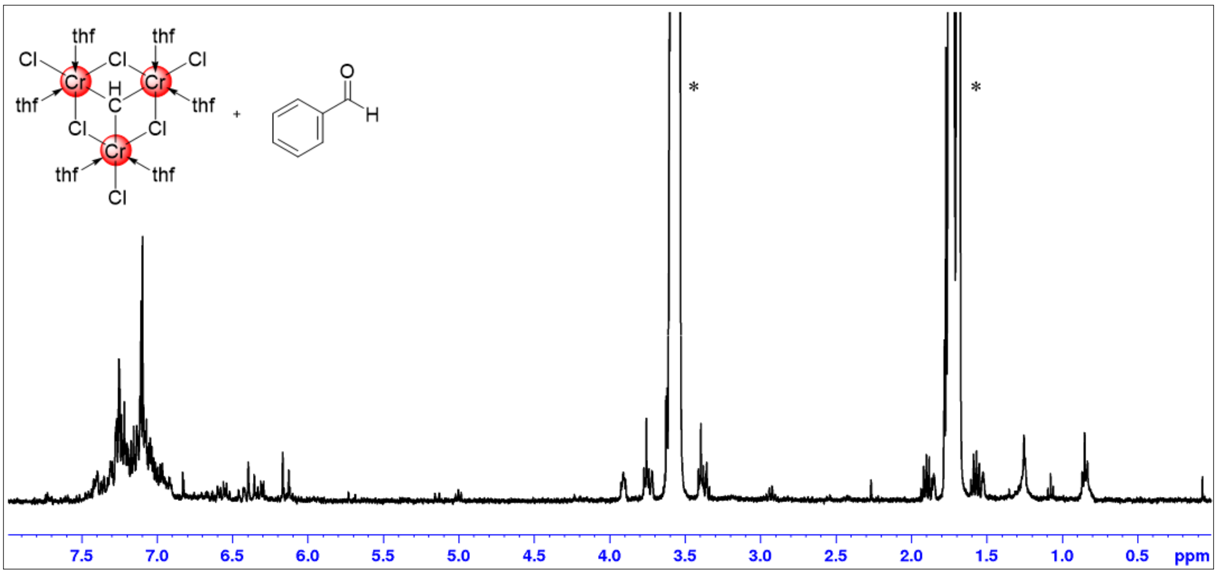

Figure S6. ^{1}H NMR spectrum (26 °C, 400.13 MHz, thf-d_8) of the product mixture of the reaction of **1** and benzaldehyde (1:1) in THF-d_8 at ambient temperature for 6 d, filtered over Al_2O_3.

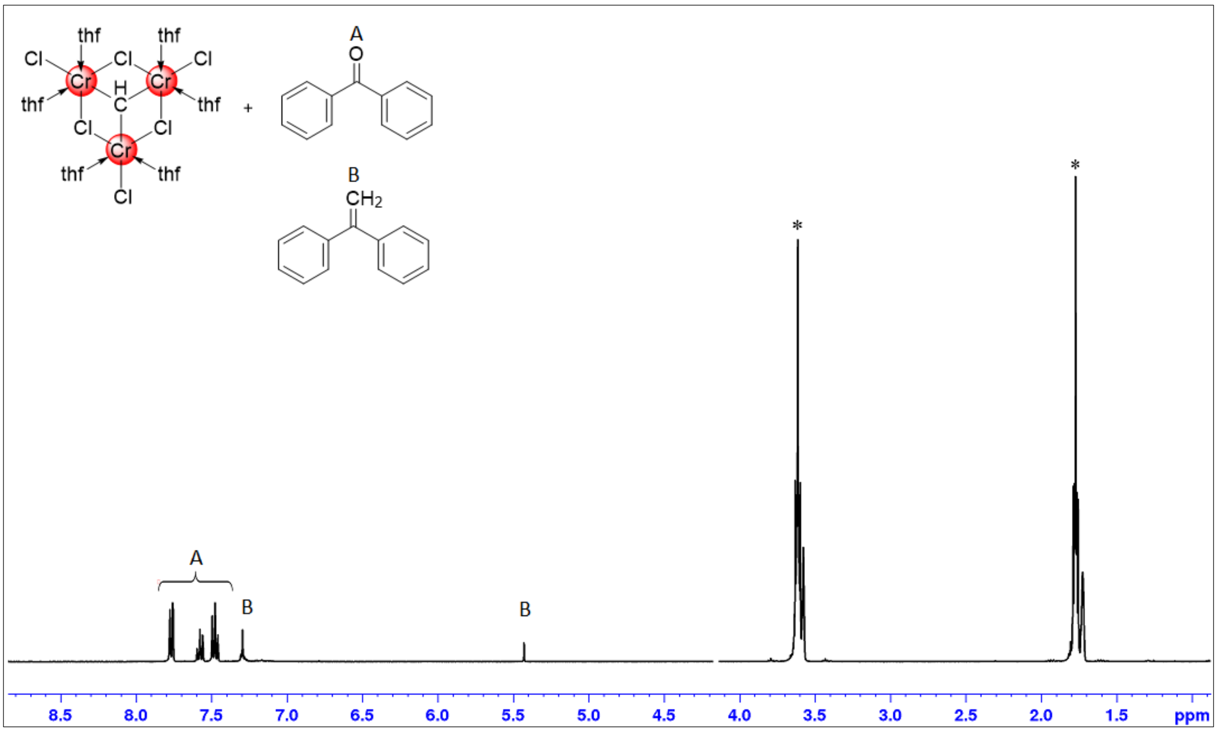

Figure S7. ^{1}H NMR spectrum (26 °C, 400.13 MHz, thf-*d₈*) of the product mixture of the reaction of **1** and benzophenone (1:1) in THF-d_8 at ambient temperature for 3 d, filtered over Al_2O_3.

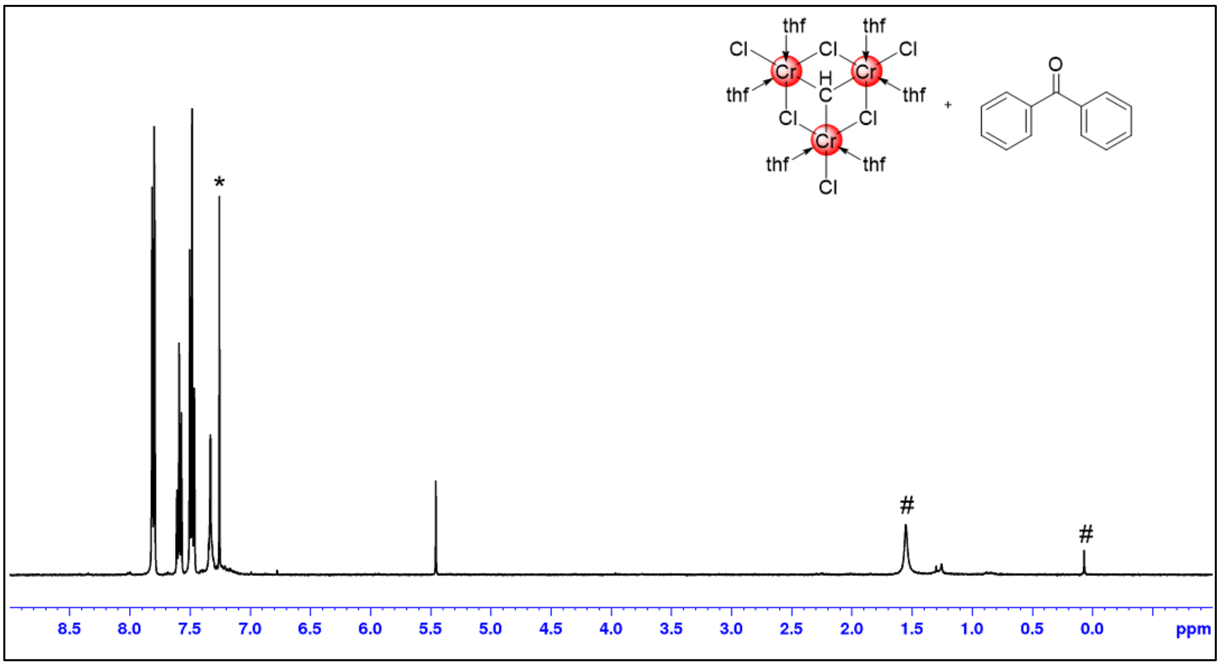

Figure S8. ^{1}H NMR spectrum (26 °C, 400.13 MHz, chloroform-*d_3*) of the product mixture of the reaction of **1** and benzophenone (1:1) in THF-d_8 at ambient temperature for 3 d, filtered over Al_2O_3. (Solvent impurities marked by #).

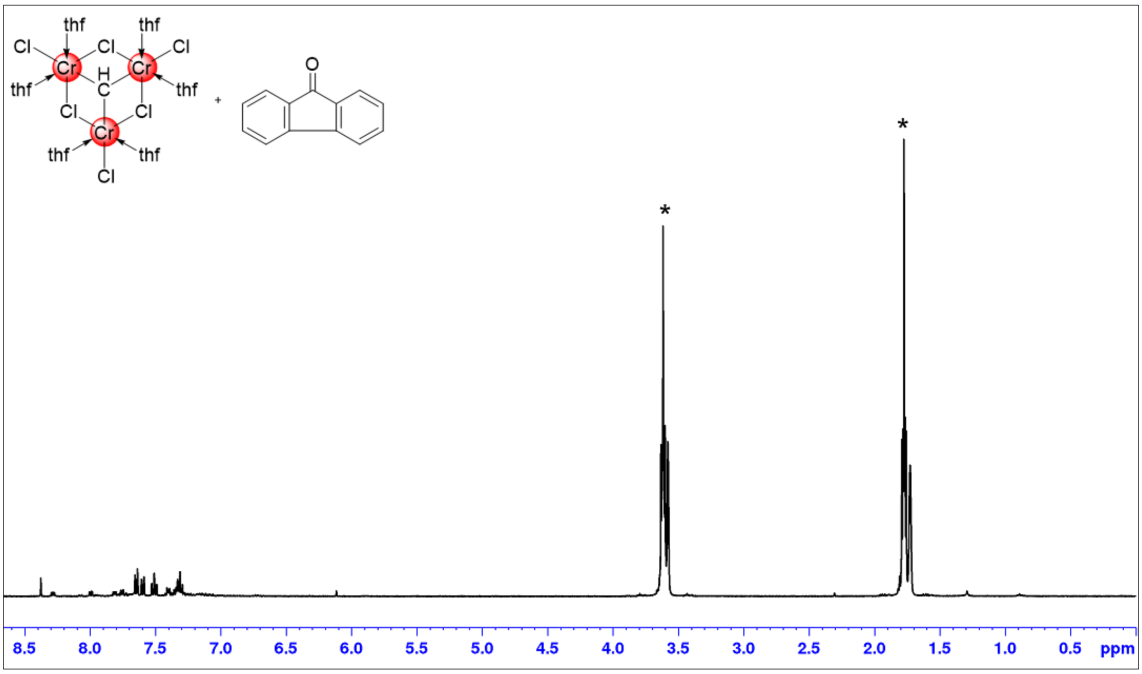

Figure S9.^{1}H NMR spectrum (26 °C, 400.13 MHz, THF-d_8) of the product mixture of the reaction of **1** and 9-fluorenone (1:1) in THF-d_8 at ambient temperature for 3 d, filtered over Al_2O_3.

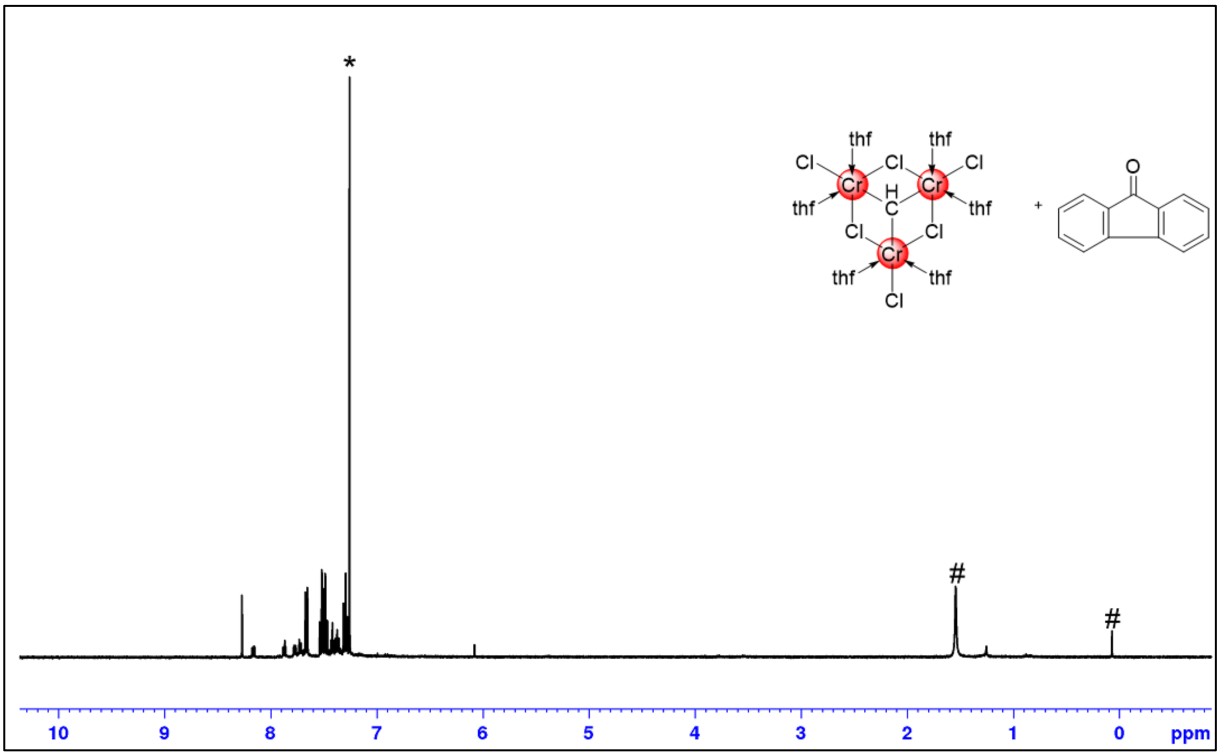

Figure S10.^{1}H NMR spectrum (26 °C, 400.13 MHz, chloroform-d_3) of the product mixture of the reaction of **1** and 9-fluorenone (1:1) in THF-d_8 at ambient temperature for 3 d, filtered over Al_2O_3.(Solvent impurities marked by #)

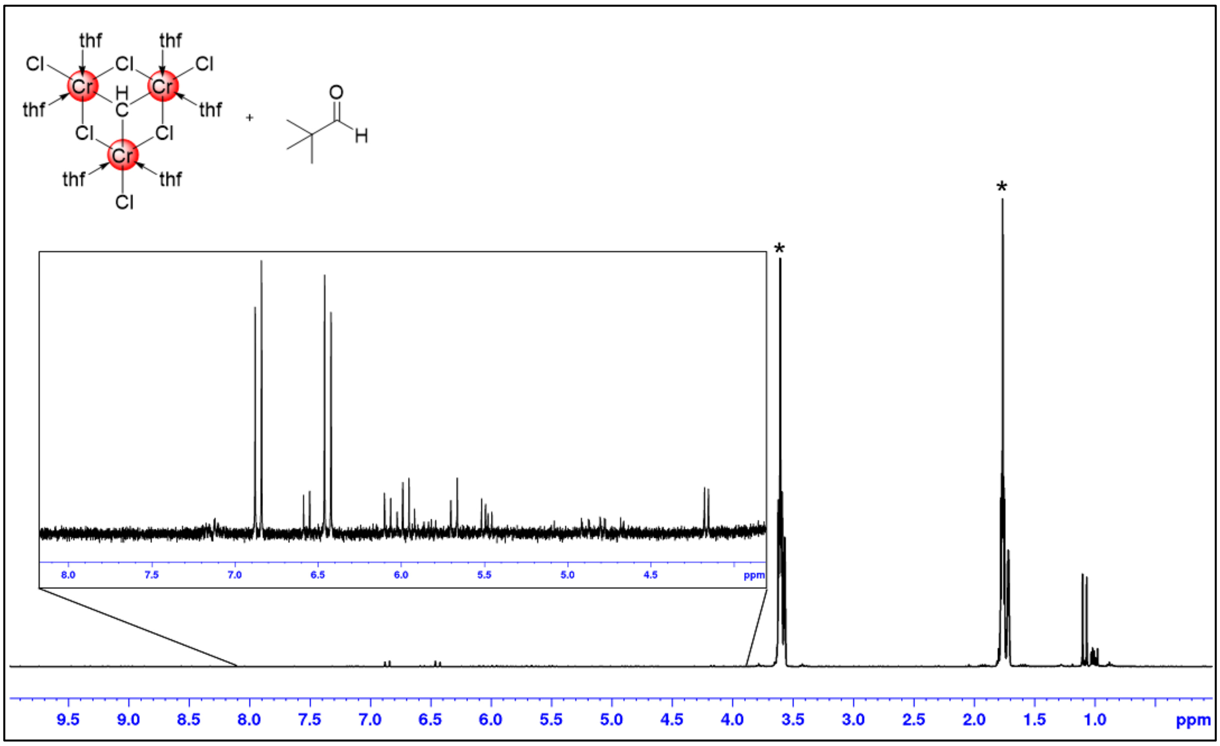

Figure S11. ^{1}H NMR spectrum (26 °C, 400.13 MHz, THF-d_8) of the product mixture of the reaction of **1** and pivalaldehyde (1:1) in THF-d_8 at ambient temperature for 3 d, filtered over Al_2O_3.

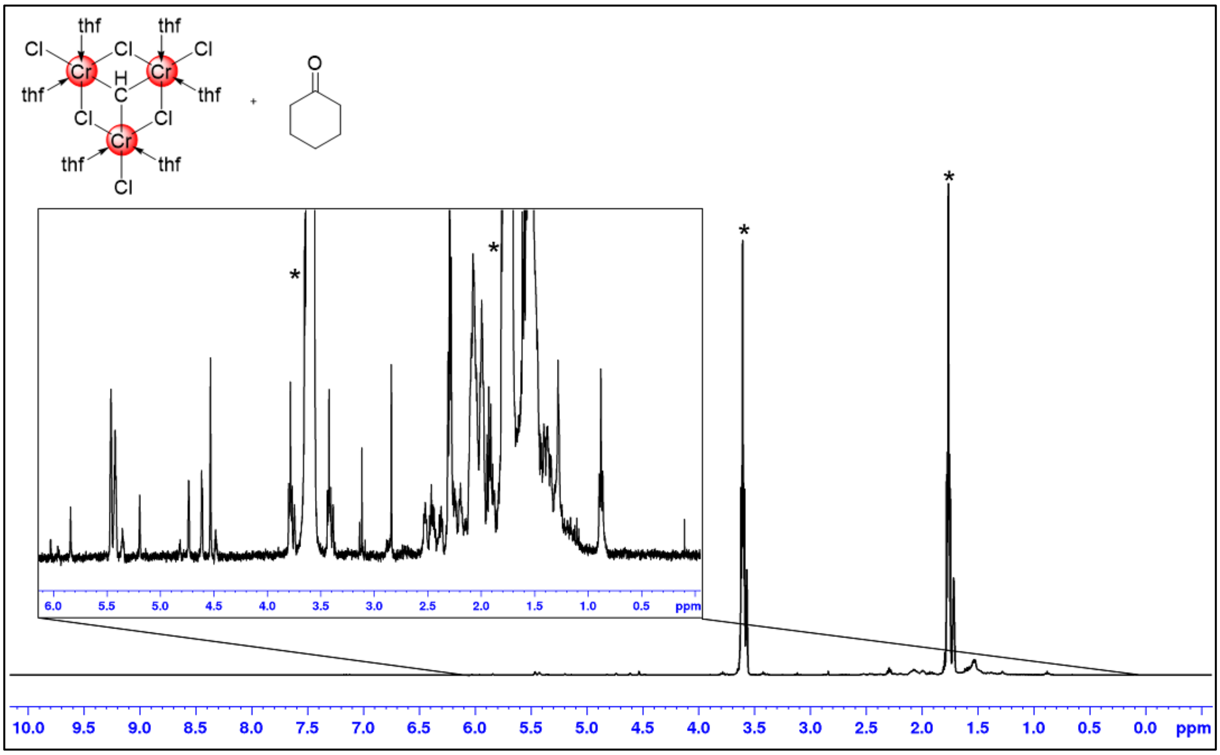

Figure S12. ^{1}H NMR spectrum (26 °C, 400.13 MHz, THF-d_8) of the product mixture of the reaction of **1** and cyclohexanone (1:1) in THF-d_8 at ambient for 3 d, filtered over Al_2O_3.

UV/Vis Spectra

Steps/artifacts at 360 nm are due to a switch of light sources of the spectrometer.

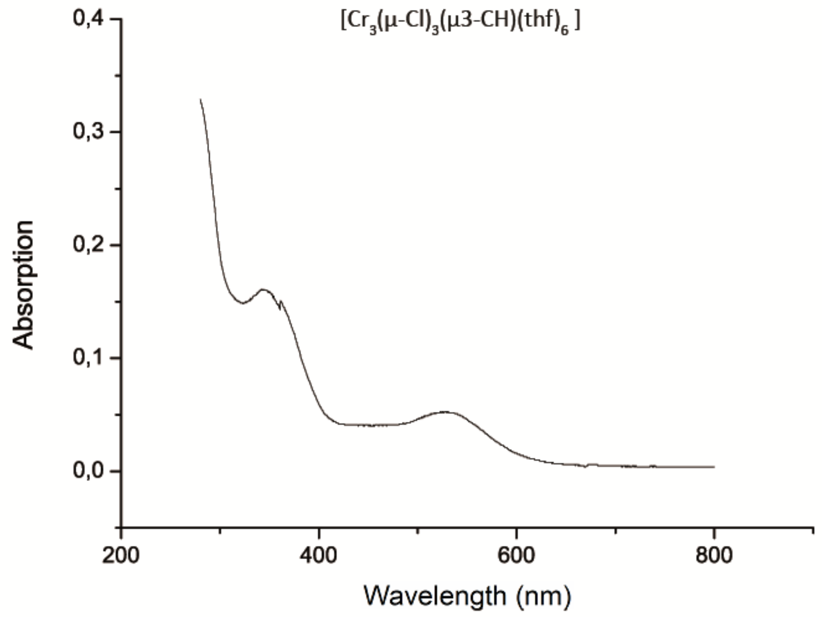

Figure S13. UV/Vis spectrum of **1** in THF at 20 °C.

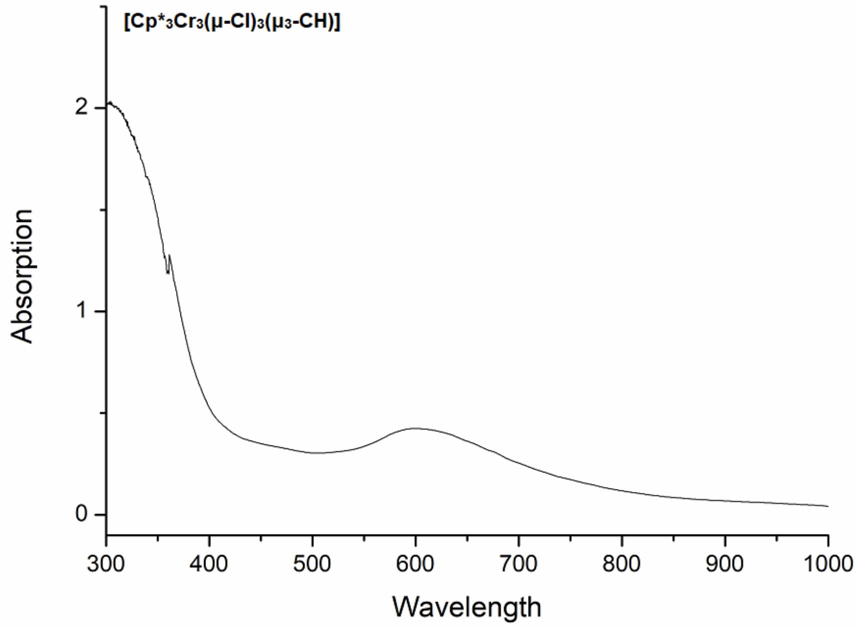

Figure S14. UV/Vis spectrum of **3** in THF at 20 °C.

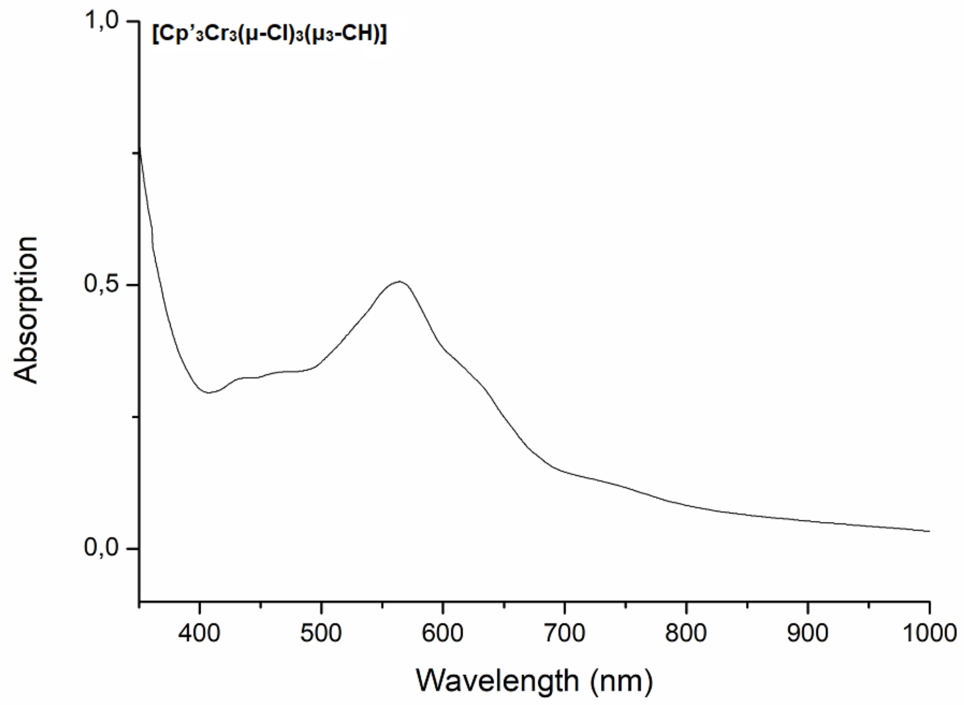

Figure S15. UV/Vis spectrum of **6** in THF at 20 °C.

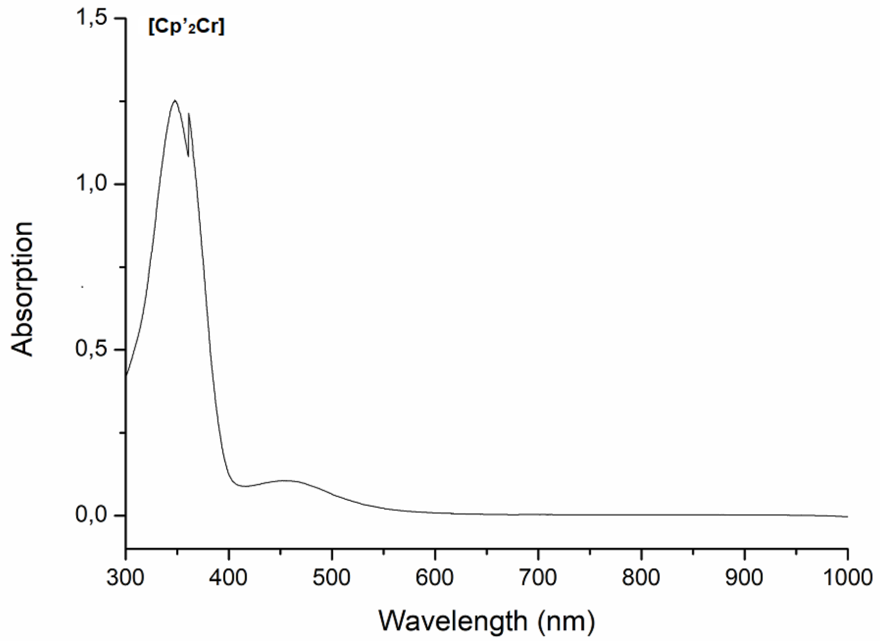

Figure S16. UV/Vis spectrum of **7** in THF at 20 °C.

Crystallographic Details

X-Ray Crystallography and Crystal Structure Determinations. Crystals for XRD analysis were grown from saturated solutions of toluene, thf, *n*-hexane or *n*-pentane. Suitable crystals were handpicked in a glovebox, coated with Parabar 10312, and stored on microscope slides before mounting outside the glovebox onto a micro loop. Data collection were done on a Bruker APEX II Duo diffractometer by using QUAZAR optics and Mo K_α (λ = 0.71073 Å). The data collection strategy was determined using COSMO[6] employing ω scans. Raw data were processed by APEX 3[7] and SAINT,[8] corrections for absorption effects were applied using SADABS.[9] Structure **2b** was refined as a twin and for absorption correction TWINABS[10] has been applied. The structures were solved by direct methods and refined against all data by full-matrix least-squares methods on F^2 using SHELXTL[11] and SHELXLE.[12] All atoms except hydrogen atoms were refined anisotropically. Disorders were modelled using DSR,[13] a program for refinement of disordered structures with SHELXL. Plots were generated by using CCDC Mercury 3.19.1.[14] Further details regarding the refinement and crystallographic data are listed in Tables S1 and S2, and in the CIF files.

For compound **2a**, **2b**, and **5** complete refinement of the structure was not possible, due to bad crystal quality. Only a connectivity is given for all three crystal structures. For compound **1** and **6**, the methylidyne hydrogen atoms were found in the Fourier map. The one in compound **3** is located on a three-fold axis and therefore was calculated using HFIX13.

Table S1. Crystallographic data for compound **1**, **2a**, **2b, 3** and **4**

	$[Cr_3Cl_3(\mu\text{-}Cl)_3(\mu_3\text{-}CH)(thf)_6]$ (1)	$[Cr_4(\mu\text{-}Cl)_4(\mu\text{-}I)_2(\mu_4\text{-}O)(thf)_4]$ (2a)	$[Cr_4(\mu\text{-}Cl)_{4.4}(\mu\text{-}I)_{2.6}(\mu_4\text{-}O)(thp)_4]$ (2b)	$[Cp^*{}_3Cr_3(\mu\text{-}Cl)_3(\mu_3\text{-}CH)]$ (3)	$[Cp^*CrCl_2(thf)]$ (4)
**Formula**	$C_{29}H_{57}Cl_6Cr_3O_7$	$C_{20}H_{40}Cl_4Cr_4I_2O_6$	$C_{25}H_{50}Cl_{4.4}Cr_4I_{2.6}O_6$	$C_{31}H_{46}Cl_3Cr_3$	$C_{14}H_{23}Cl_2CrO_1$
**CCDC**	2084207	2084206	2084205	2084204	2084210
**M_r [g mol^{-1}]**	886.44	980.12	1140.57	681.03	330.22
**color**	red/ plates	green/ block	green/ blocks	green/	blue/block
**crystal system**	triclinic	monoclinic	monoclinic	trigonal	monoclinic
**space group**	$P\bar{1}$	Cc	$P2_1/c$	R3	Pc
**a [Å]**	11.5732(3)	21.388(4)	15.8809(10)	18.1645(6)	12.2585(14)
**b [Å]**	12.3378(3)	13.308(3)	12.2956(7)	18.1645(6)	17.595(2)
**c [Å]**	14.2492(3)	14.340(3)	20.7392(12)	7.8855(3)	15.0197(17)
**α [°]**	106.3760(10)	90	90	90	90
**β [°]**	101.9940(10)	109.833(2)	104.5370(10)	90	103.544(2)
**γ [°]**	93.0250(10)	90	90	120	90
**V [Å^3]**	1895.97(8)	3839.52	3920.0(4)	2253.24(17)	3149.5(6)
**Z**	2	8	4	3	8
**T [K]**	100(2)	170(2)	100(2)	173(2)	100(2)
**ρ_{calcd} [g cm^{-3}]**	1.553			1.506	1.393
**μ [mm^{-1}]**	1.309			1.353	1.053
**F (000)**	922			1065	1384
**total reflns**	68227			17191	41526
**unique reflns**	8347			2813	12856
**Data/restrains/parameter**	8347 / 194 / 400			2813 / 1 / 118	12856 / 192 / 716
**R_1(I>2σ)[a]**	0.0396			0.0189	0.0630
**ωR_2 (I>2σ)[a]**	0.0963			0.0483	0.1674
**R_1 (all data)**	00570			0.0193	0.0846
**ωR_2 (all data)**	0.1064			0.0486	0.1867
**GOF**	1.038			1.091	1.062

[a] $R_1 = \Sigma(||F_0|-|F_c||)/\Sigma|F_0|, F_0 > 4\sigma(F_0)$. $wR_2 = \{\Sigma[w(F_0^2-F_c^2)^2/\Sigma[w(F_0^2)^2]\}^{1/2}$.

Table S2. Crystallographic data for compound **5**, **6**, **7** and **8**

	**[Cp*Cr(μ-Cl)(μ-I)]₂ (5)**	**[Cp'₃Cr₃(μ-Cl)₃(μ₃-CH)] (6)**	**[Cp'₂Cr] (7)**	**{(Cp'CrCl₃)[Li(thf)₂]} (8)**
**Formula**	$C_{20}H_{30}Cl_{10.83}Cr_2I_{1.17}$	$C_{25}H_{40}Cl_3Cr_3Si_3$	$C_{16}H_{26}CrSi_2$	$C_{16}H_{29}Cl_3CrLiO_2Si$
**CCDC**	2084212	2084208	2084209	2084211
**M_r [g mol^{-1}]**	552.79	687.19	326.55	446.77
**color**	blue/ block	violet/ needles	orange	blue
**crystal system**	monoclinic	monoclinic	monoclinic	orthorhombic
**space group**	$P2_1/n$	$P2_1/c$	$P2_1/c$	$Pna2_1$
**a [Å]**	11.145(4)	13.2665(6)	6.1193(11)	29.821(2)
**b [Å]**	13.403(6)	18.3676(8)	8.0476(14)	10.1555(10)
**c [Å]**	14.649	13.6325(6)	17.290(3)	20.468(2)
**α [°]**	90	90	90	90
**β [°]**	101.542(6)	107.6150(10)	94.932(2)	90
**γ [°]**	90	90	90	90
**V [Å^3]**	2143.9(15)	3166.1(2)	848.(3)	4328.0(8)
**Z**	4	4	2	8
**T [K]**	173(2)	100(2)	173(2)	100(2)
**ρ_{calcd} [g cm^{-3}]**		1.442	1.278	1.371
**μ [mm^{-1}]**		1.392	0.802	0.960
**F (000)**		1420	348	1864
**total reflns**		84588	9415	45447
**unique reflns**		8510	2225	10157
**Data/restrains/parameter**		8510 / 0 / 320	2225 / 0 / 91	10157 / 1 / 439
**R_1(I>2σ)[a]**		R_1 = 0.0261	R1 = 0.0376	R1 = 0.0399
**ωR_2 (I>2σ)[a]**		ωR_2 = 0.0670	ωR_2 = 0.0933	ωR_2 = 0.0863
**R_1 (all data)**		R_1 = 0.0307	R1 = 0.0477	R_1 = 0.0531
**ωR_2 (all data)**		ωR_2 = 0.0702	ωR_2 = 0.1009	ωR_2 = 0.0940
**GOF**		1.051	1.053	1.027

[a] $R_1 = \Sigma(||F_0|-|F_c||)/\Sigma|F_0|, F_0 > 4\sigma(F_0)$. $wR_2 = \{\Sigma[w(F_0^2-F_c^2)^2/\Sigma[w(F_0^2)^2]\}^{1/2}$.

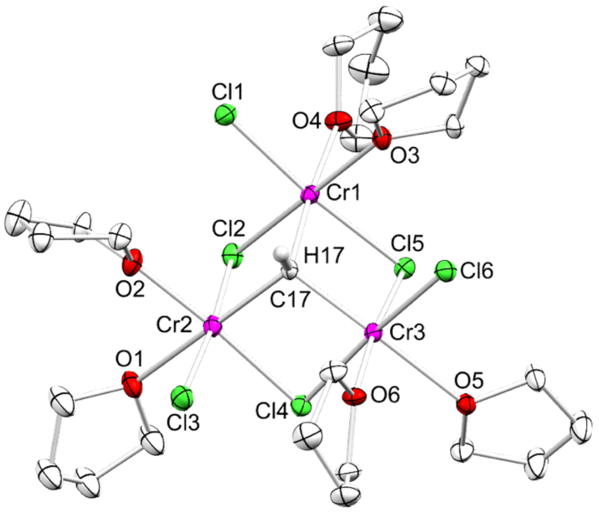

Figure S17. MERCURY representation (50% probability ellipsoids) of [$Cr_3Cl_3(\mu\text{-}Cl)_3(\mu_3\text{-}CH)(thf)_6$] (**1**). Hydrogen atoms (except for H17) and co-crystallized solvent molecules (thf) are omitted for clarity. Selected interatomic distances (Å) and angles (°): Cr1–C17 2.022(3), Cr2–C17 2.019(3), Cr3–C17 2.018(3), C17–H17 0.84(3), Cr1–O3 2.0433(18), Cr1–O4 2.2409(19), Cr1–Cl1 2.3328(7), Cr1–Cl2 2.3876(7),Cr1–Cl5 2.4186(7), Cr2–Cl2 2.4159(8), Cr2–Cl3 2.3291(8), Cr2–Cl4 2.3769(7), Cr3–Cl4 2.4071(7),Cr3–Cl5 2.3975(7), Cr3–Cl6 2.3141(7), C17–H17 0.84(3); Cr1–C17–Cr2 103.66(12), Cr1–C17–Cr3 102.72(12), Cr2–C17–Cr3 103.43(12), Cr1–Cl2–Cr2 82.83(2), Cr2–Cl4–Cr3 82.96(2), Cr3–Cl5–Cr1 81.88(2).

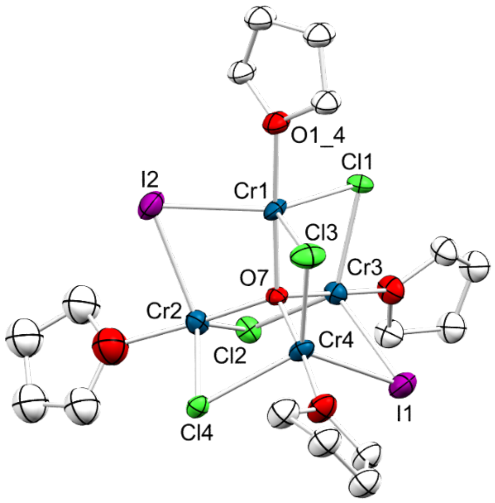

Figure S18. MERCURY representation of the connectivity (30% probability ellipsoids) of [$Cr_4(\mu\text{-}Cl)_4(\mu\text{-}I)_2(\mu_4\text{-}O)(thf)_4$]*thf (**2a**). Hydrogen atoms and lattice solvent (thf) are omitted for clarity.

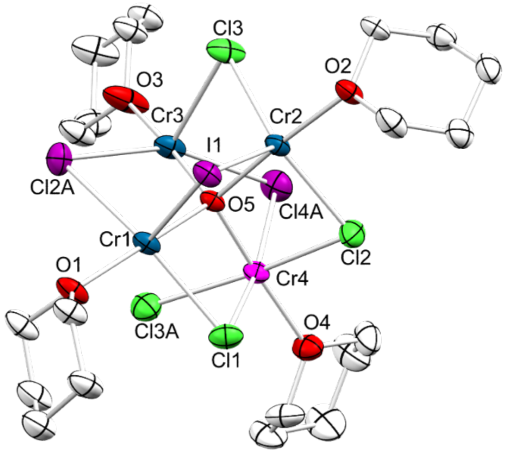

Figure S19. MERCURY representation (50% probability ellipsoids) of [$Cr_4Cl(\mu\text{-}Cl)_4(\mu\text{-}I)_2(\mu_4\text{-}O)(thp)_4$] (**2b**). Hydrogen atoms and co-crystallized solvent molecules (THP) are omitted for clarity.

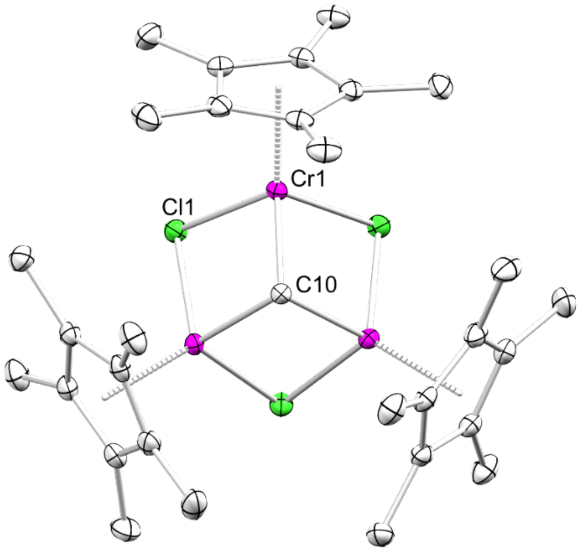

Figure S20. MERCURY representation (50% probability ellipsoids) of [$(\eta_5\text{-}C_5Me_5)_3Cr_3(\mu\text{-}Cl)_3(\mu_3\text{-}CH)$] (**3**). Hydrogen atoms are omitted for clarity. Selected interatomic distances (Å) and angles (°): Cr1–C10 2.0109(19), Cr1–Cl1 2.3416(5), Cr1–Cr1' 2.9103(5), Cr1–Cr1'' 2.9103(5), Cr1''–Cl1 2.3615(5), Cr1–$Cp_{centroid}$ 1.903 (calculated with Mercury); C10–Cr1–Cl1 95.73(6), C10–Cr1–Cl1' 95.11(6), C10–Cr1–Cr1' 43.65(5), Cl1–Cr1–Cl1' 92.15(2), Cl1'–Cr1–Cr1' 96.046(13), Cl1'–Cr1–Cr1' 51.46(13), C10–Cr1–Cr1'' 43.65(5), Cr1'–Cr1–Cr1'' 60.002(1), Cr1–Cl1–Cr1'' 76.456(18).

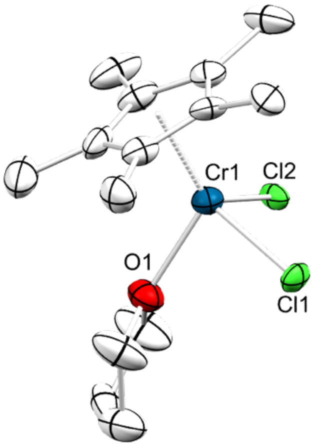

Figure S21. MERCURY representation (50% probability ellipsoids) of [Cp*$CrCl_2$(thf)] (**4**). Hydrogen atoms and the three additional molecules in the unit cell are omitted for clarity. Selected interatomic distances (Å) and angles (°): Cr1–Cl1 2.388(3), Cr1–Cl2 2.349(3), Cr1–O1 2.060(8), Cr1–$Cp_{centroid}$ 1.878 (calculated with Mercury); Cl1–Cr1–Cl2 98.44(11).

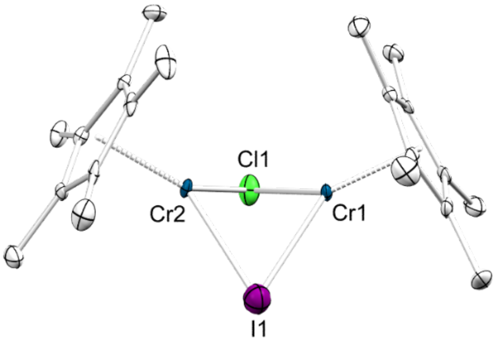

Figure S22. MERCURY representation (50% probability ellipsoids) of [$(Cp^*Cr)_2$(μ-Cl)(μ-I)] (**5**). Hydrogen atoms and the second molecule in the unit cell are omitted for clarity. Selected interatomic distances (Å) and angles (°): Cr1–I1 2.929(1), Cr2–I1 2.627(1), Cr1–Cl1 2.32(1), Cr2–Cl1 2.33(2),Cr1–$Cp_{centroid}$ 1.909, Cr2–$Cp_{centroid}$ 1.920 (calculated with Mercury); Cr1–I1–Cr2 58.60(3), Cr1–Cl1–Cr2 67.2(4), I1–Cr2–Cl1 95.4(4).

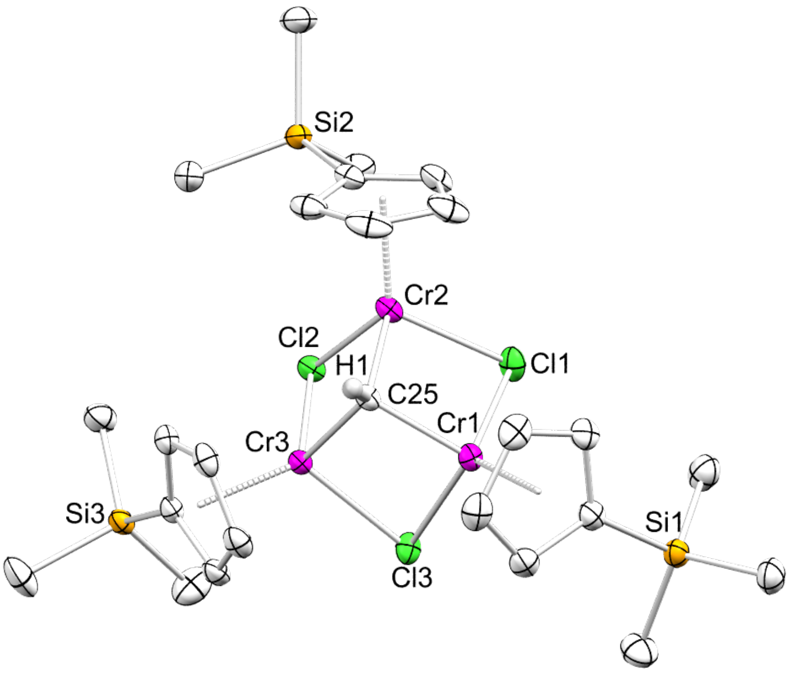

Figure S23. MERCURY representation (50% probability ellipsoids) of [(η5-$C_5H_4SiMe_3)_3Cr_3$(μ-Cl)$_3$(μ$_3$-CH)] (**6**). Hydrogen atoms (except for H1) are omitted for clarity. Selected interatomic distances (Å) and angles (°): Cr1–C25 1.9846(15), Cr2–C25 1.9806(14), Cr3–C25 1.9822(14), Cr1–Cr2 2.8193(3), Cr1–Cr3 2.8318(3), Cr2–Cr3 2.8363(3), Cr3–Cl2 2.3316(4), Cr3–Cl3 2.3519(4), Cr1–Cl1 2.3243(4), Cr2–Cl1 2-3270(4), Cr2–Cl2 2.3355(4), Cr1–Cl3 2.3479(4), C25–H1 0.965(19), Cr1–Cp$_{centroid}$ 1.873, Cr2–Cp$_{centroid}$ 1.871, Cr3–Cp$_{centroid}$ 1.868 (calculated with Mercury); Cr(1)–Cl(1)–Cr(2) 74.621(13), Cr(1)–Cl(3)–Cr(3) 74.102(13), Cr(3)–Cl(2)–Cr(2) 74.850(12), Cl(1)–Cr(1)–Cl(3) 93.396(16), Cl(2)–Cr(3)–Cl(3) 93.783(15), Cl(1)–Cr(2)–Cl(2) 91.652(15), C(25)–Cr(1)–Cl(1) 97.36(4), C(25)–Cr(2)–Cl(2) 96.82(4), C(25)–Cr(3)–Cl(3) 97.31(4), C(25)–Cr(1)–Cl(3) 97.37(4), C(25)–Cr(2)–Cl(1) 97.38(4), C(25)–Cr(3)–Cl(2) 96.90(4), Cr(2)–C(25)–Cr(3) 91.41(6), Cr(2)–C(25)–Cr(1) 90.64(6), Cr(3)–C(25)–Cr(1) 91.10(6).

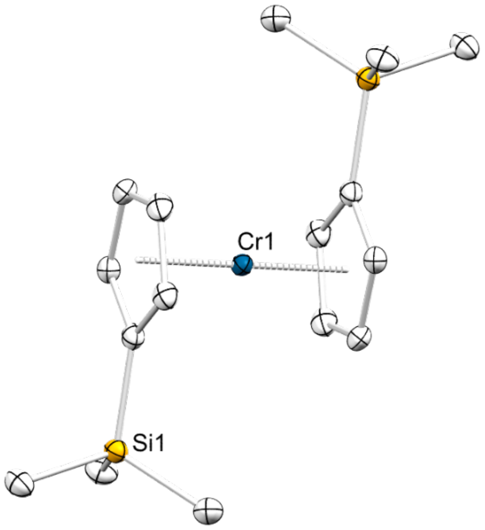

Figure S24. MERCURY representation (50% probability ellipsoids) of [(η^5-$C_5H_4SiMe_3)_2$Cr] (**7**). Hydrogen atoms are omitted for clarity. Selected interatomic distances (Å) and angles (°): Cr1–C1 2.1343(18), Cr1–C2 2.1331(17), Cr1–C3 2.1905(18), Cr1–C4 2.2106(18), Cr1–C5 2.159(2), Cr1–Cp$_{centroid}$ 1.798 (calculated with Mercury), Si1–C1 1.8602(19); Cr1–C1–Si1 127.56(9).

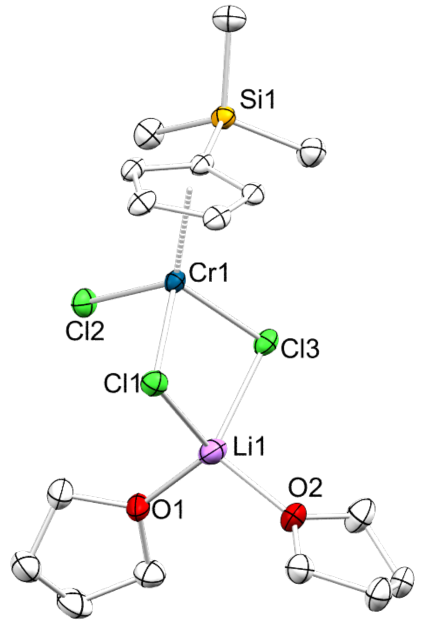

Figure S25. MERCURY representation (50% probability ellipsoids) of [(η^5-$C_5H_4SiMe_3$)CrCl(μ-Cl)$_2$Li(thf)$_2$] (**8**). Hydrogen atoms and the second molecule in the unit cell are omitted for clarity. Selected interatomic distances (Å) and angles (°): Cr1–Cl1 2.3524(12), Cr1–Cl2 2.2928(13), Cr1–Cl3 2.3387(12), Cr1–Cp$_{centroid}$ mean1.794 (calculated with Mercury), Li1–O1 1.899(9), Li1–O2 1.939(9), Li1–Cl3 2.401(8), Li1–Cl1 2.362(8), Li1–Cl3 2.401(8); Cr1–Cl1–Li1 85.8(2), Cr1–Cl3–Li1 85.3(2), Cl2–Cr1–Cl3 95.36(5), Cl2–Cr1–Cl1 97.92(5), Cl3–Cr1–Cl1 92.18(5).

Infrared Spectra

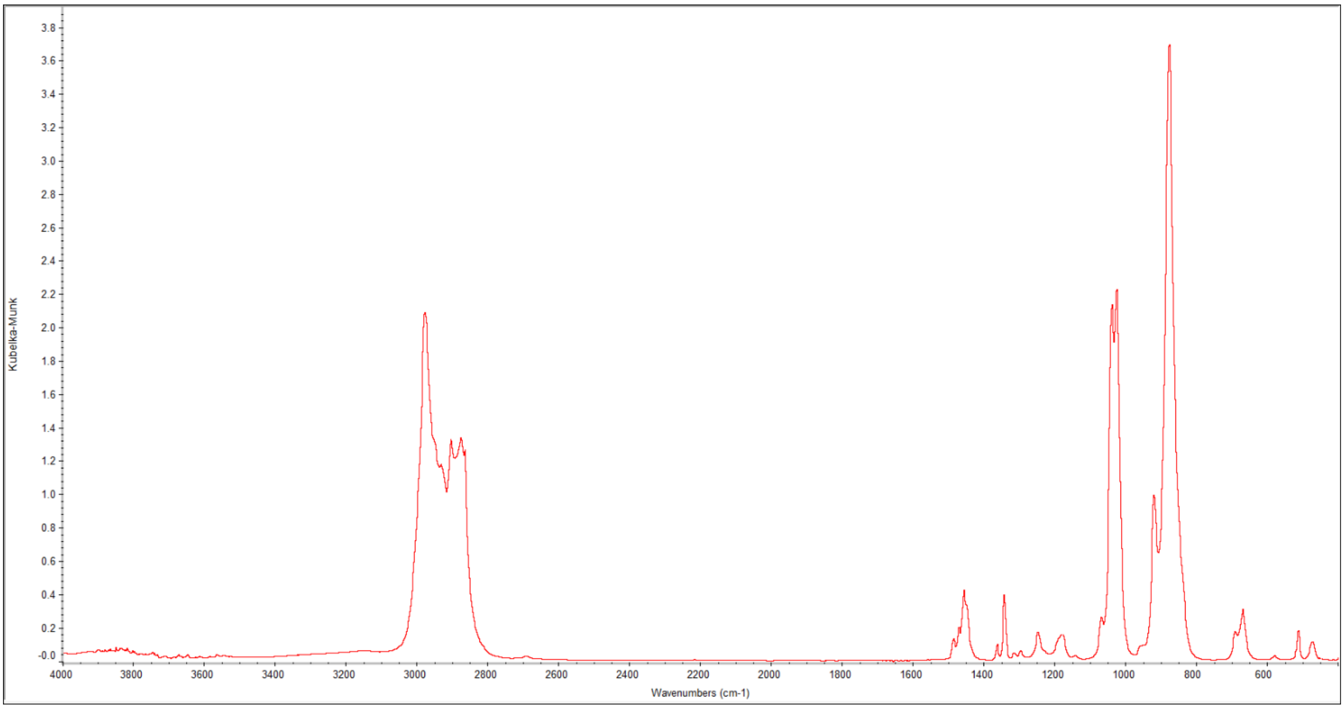

Figure S26. DRIFT spectrum of [$Cr_3(\mu_2\text{-}Cl)_3Cl_3(\mu_3\text{-}CH)(thf)_6$] (**1**) at 25 °C.

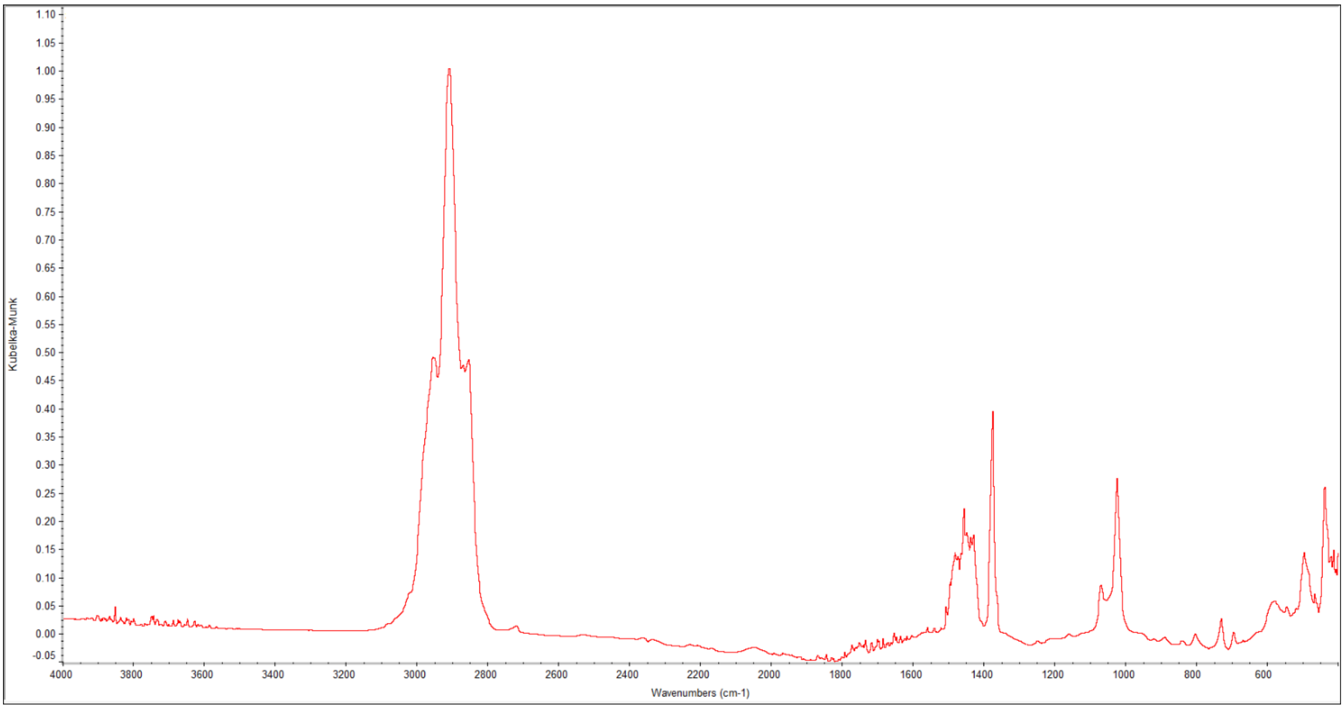

Figure S27. DRIFT spectrum of [$(\eta^5\text{-}C_5Me_5)_3Cr_3(\mu_2\text{-}Cl)_3(\mu_3\text{-}CH)$] (**3**) at 25 °C.

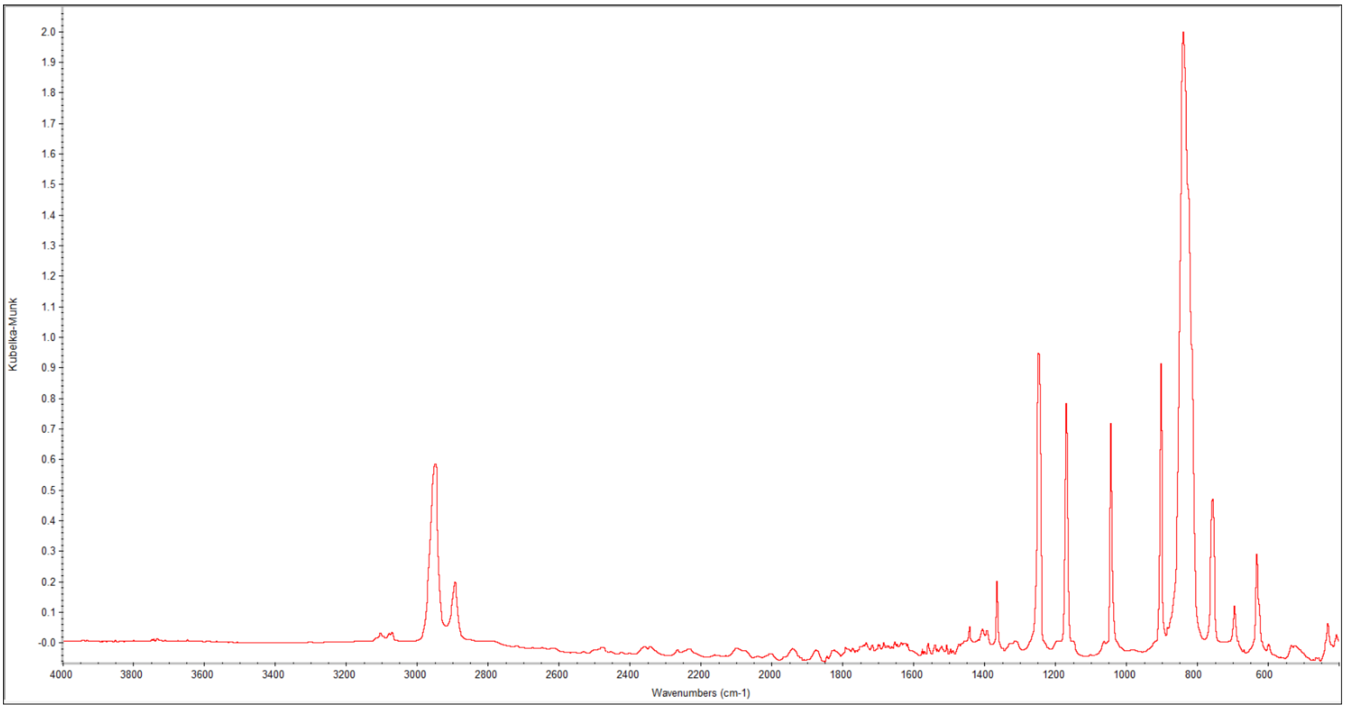

Figure S28. DRIFT spectrum of [(η[5]-$C_5H_4SiMe_3$)Cr_3(μ_2-Cl)$_3$(μ_3-CH)] (**6**) at 25 °C.

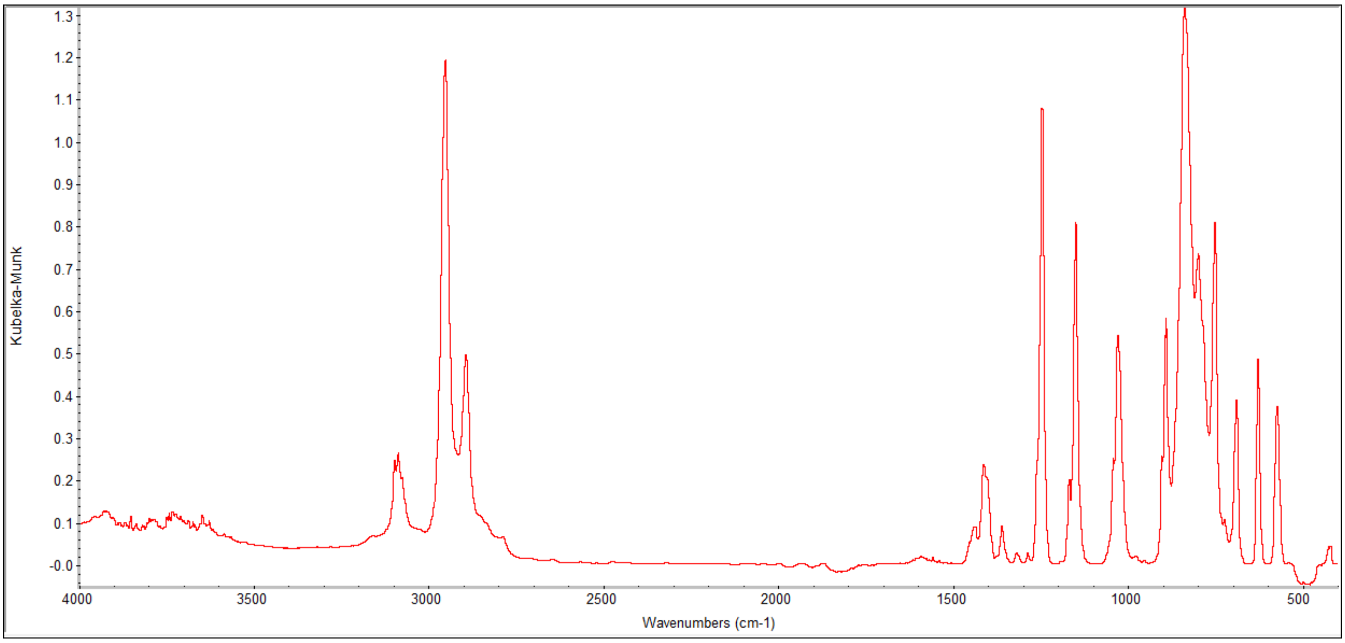

Figure S29. DRIFT spectrum of [(η[5]-$C_5H_4SiMe_3$)$_2$Cr] (**7**) at 25 °C.

SQUID Measurements

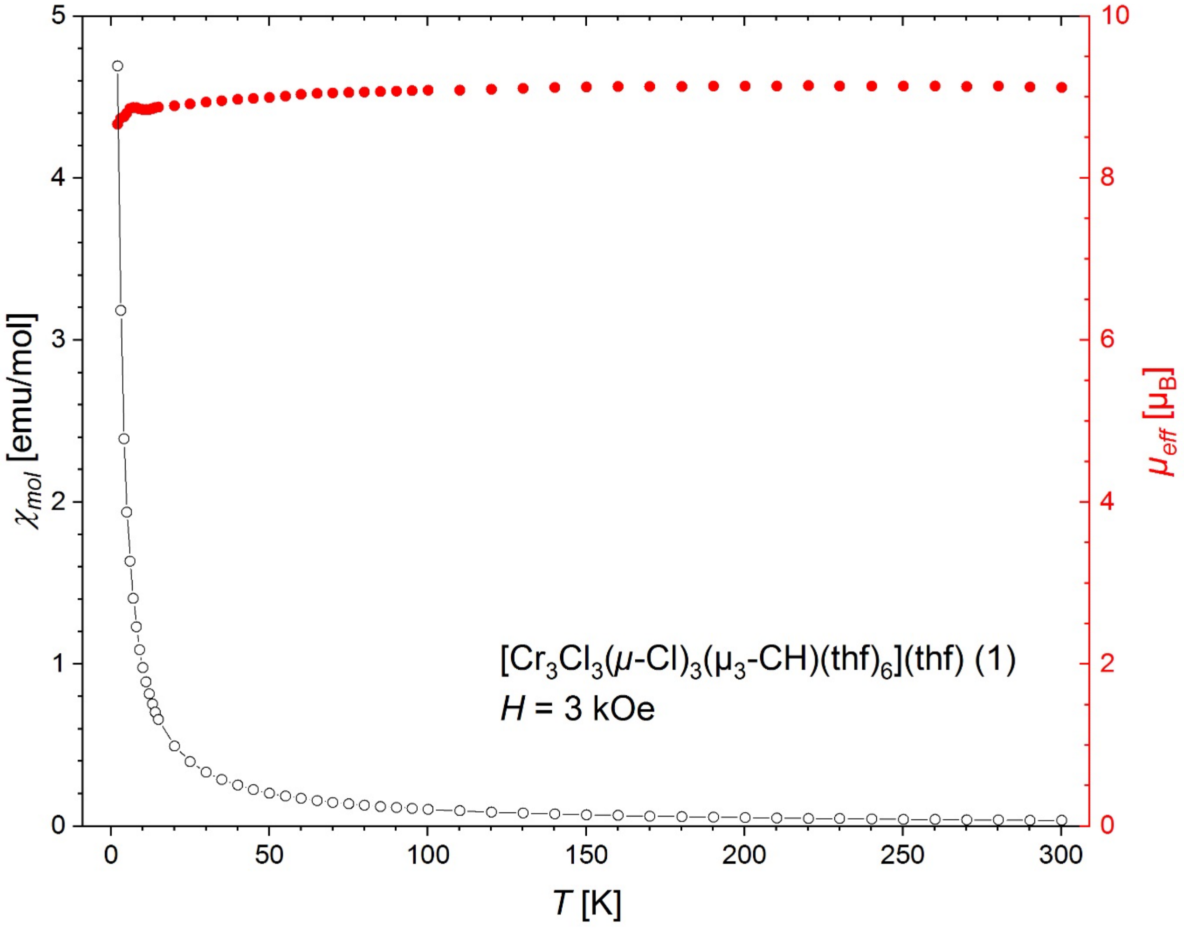

Figure 30. Temperature-dependent molar magnetic susceptibility $\chi_{mol}(T)$ (black open circles; left ordinate) and effective magnetic moment $\mu_{eff}(T)$ (red filled circles; right ordinate) as obtained by SQUID magnetic measurements on crystalline powder of **1** in an applied field H = 3 kOe. The $\chi_{mol}(T)$ data was corrected for diamagnetic contributions ($-7.243\cdot10^{-4}$ emu/mol; calculated from Pascal's constants),[15] and a spin-only g factor of 2.0 was assumed in the calculation of $\mu_{eff}(T)$. Note, that **1** contains an additional THF solvent molecule per formula unit in the crystal packing.

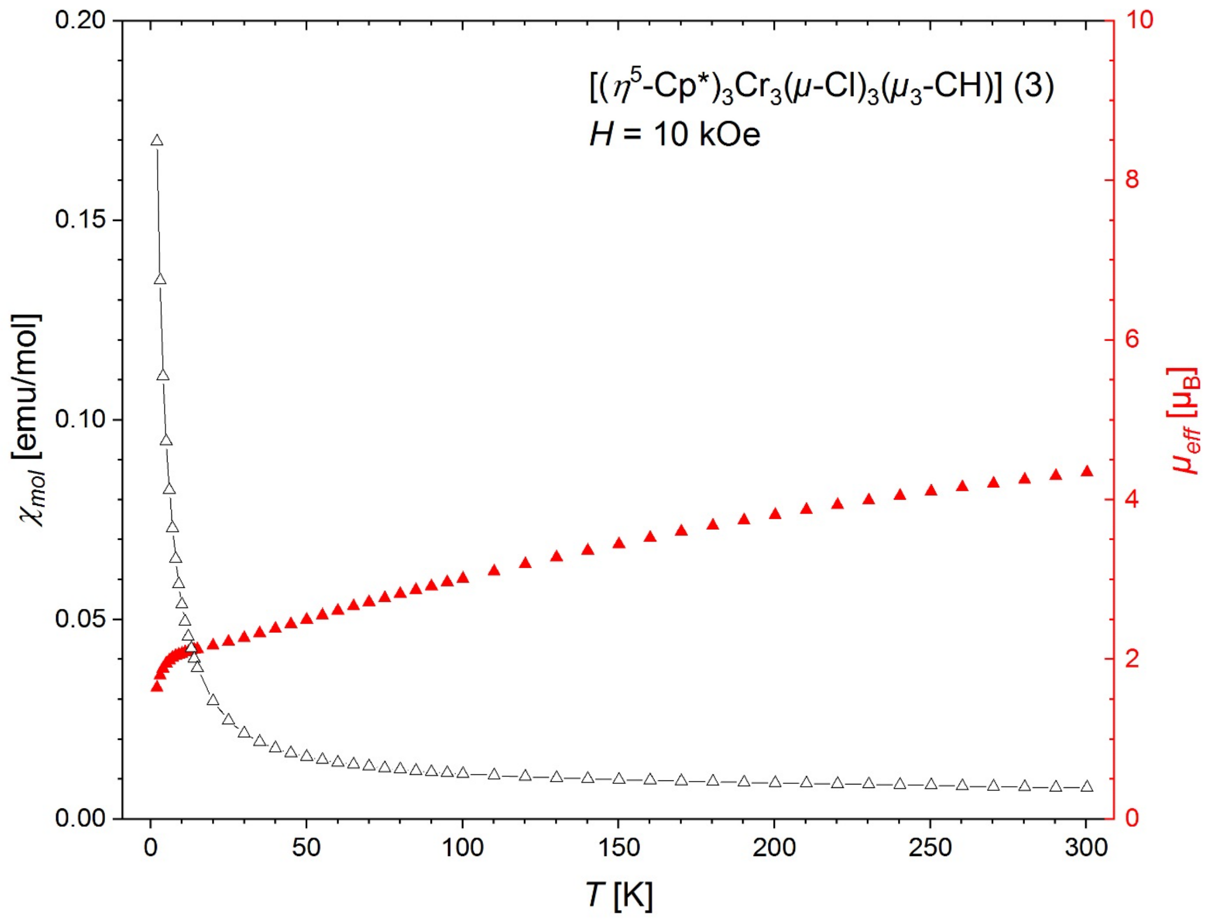

Figure 31. Temperature-dependent molar magnetic susceptibility $\chi_{mol}(T)$ (black open triangles; left ordinate) and effective magnetic moment $\mu_{eff}(T)$ (red filled triangles; right ordinate) as obtained by SQUID magnetic measurements on crystalline powder of **3** in an applied field H = 10 kOe. The $\chi_{mol}(T)$ data was corrected for diamagnetic contributions (-3.071·10^{-4} emu/mol; calculated from Pascal's constants),[15] and a spin-only g factor of 2.0 was assumed in the calculation of $\mu_{eff}(T)$.

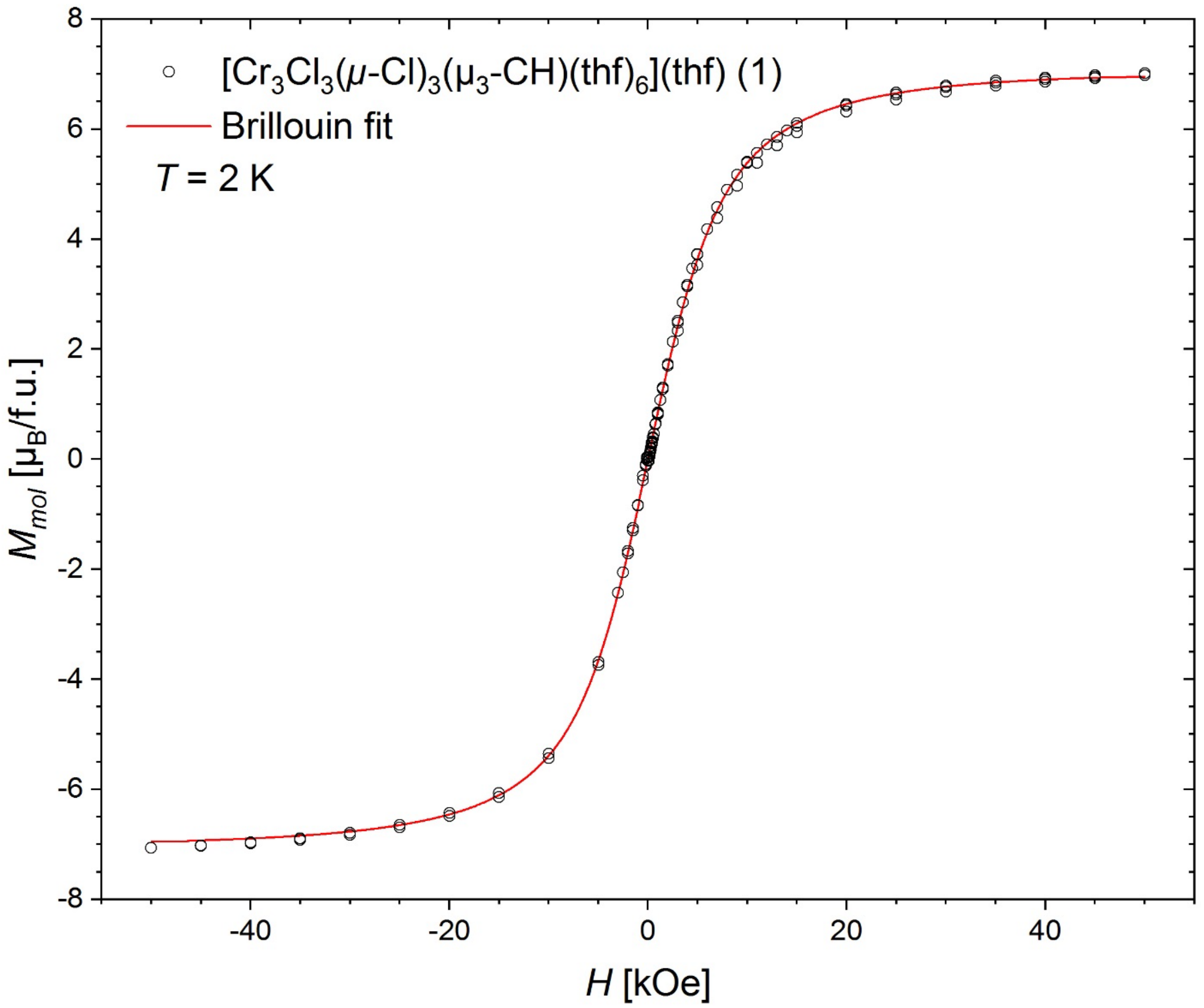

Figure 32. Field-dependent molar magnetization $M_{mol}(H)$ (black open circles) as obtained by SQUID magnetic measurements on crystalline powder of **1** at a temperature T = 2 K. A fit of the data with a Brillouin function (red line; assuming a spin-only g factor of 2.0) yields a total spin quantum number S = 4.45(4). Note, that **1** contains an additional THF solvent molecule per formula unit in the crystal packing.

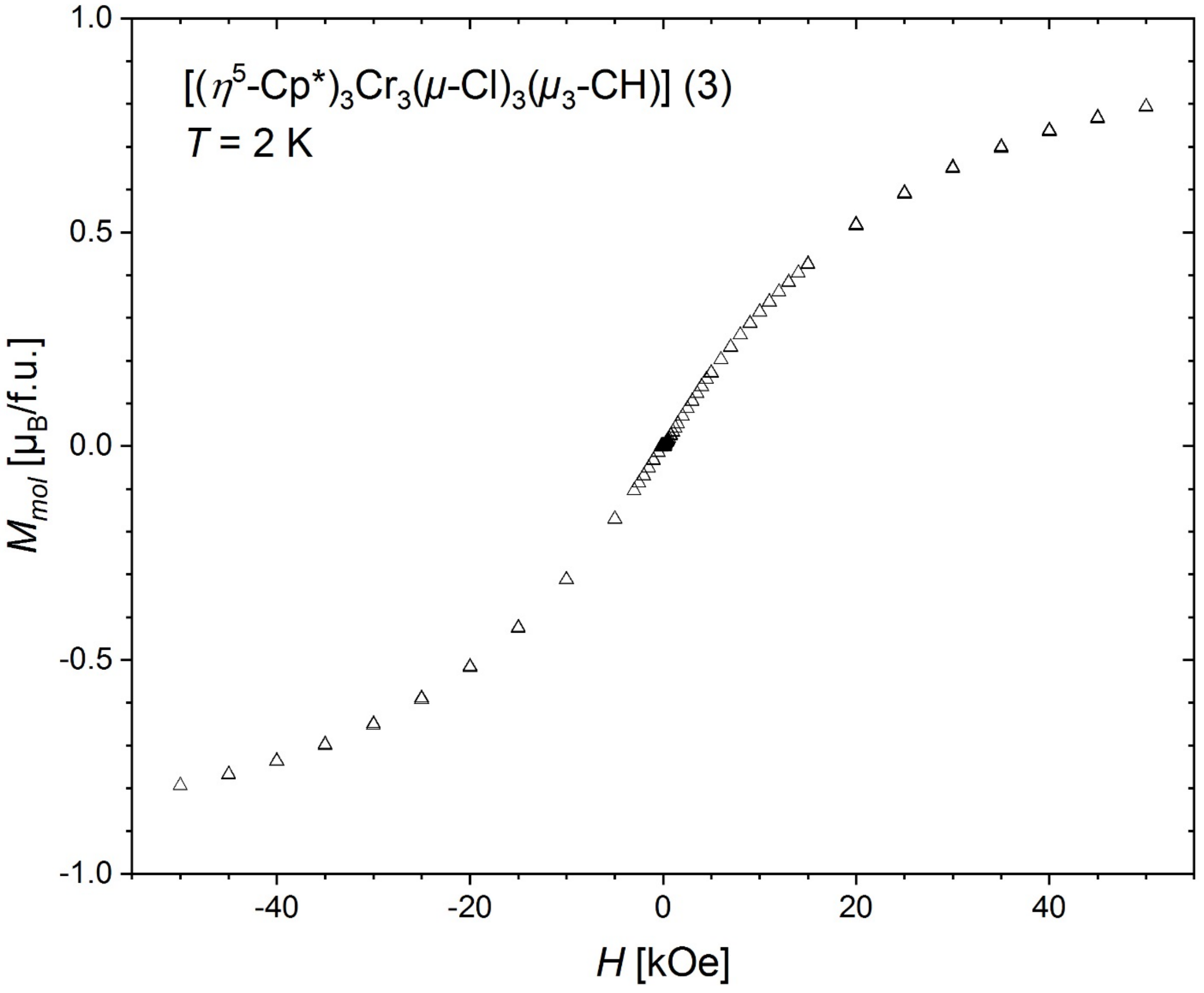

Figure S33. Field-dependent molar magnetization $M_{mol}(H)$ (black open circles) as obtained by SQUID magnetic measurements on crystalline powder of **3** at a temperature T = 2 K.

Gas Chromatography – Mass Spectrometry

GC/MS was performed on an *Agilent* Q 5973 with electron impact ionization.The obtained mass spectral data were analyzed with *Agilent* MassHunter (version 10.0.368) and compared to Main EI MS Library (mainlib). Further product suggestions for found compounds are based on plausible isostructural known compounds, that are not listed in the databases used by *Agilent* MassHunter and are marked by square brackets.

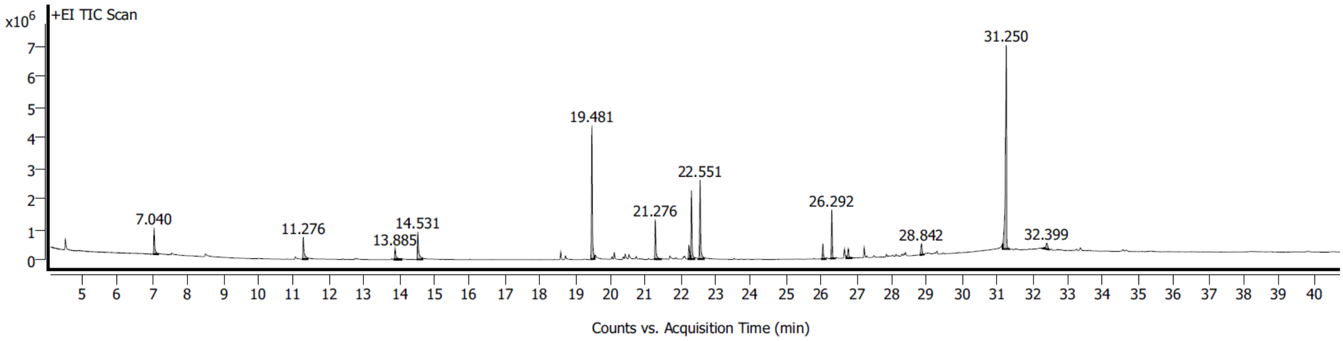

Figure S34. Gas chromatogram of the filtered reaction mixture of **1** with 1 equivalent of benzaldehyde in THF-d_8 at ambient temperature for 6 d.

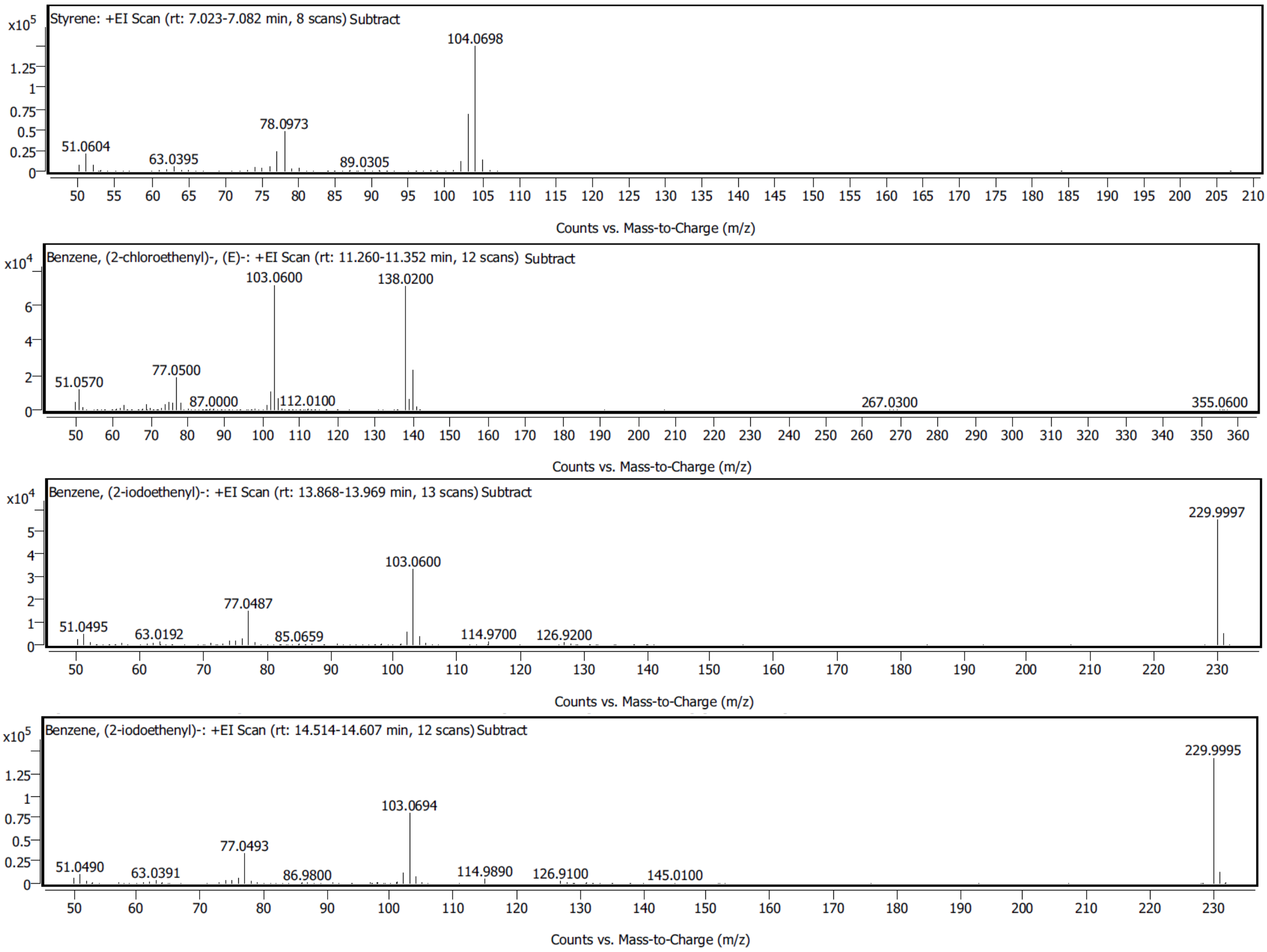

Figure S35. EI-mass spectra of the GC peaks of the filtered reaction mixture of **1** with 1 equivalent of benzaldehyde in THF-d_8 at ambient temperature for 6 d from 7.023 min to 14.607 min, evaluated by *Agilent* MassHunter.

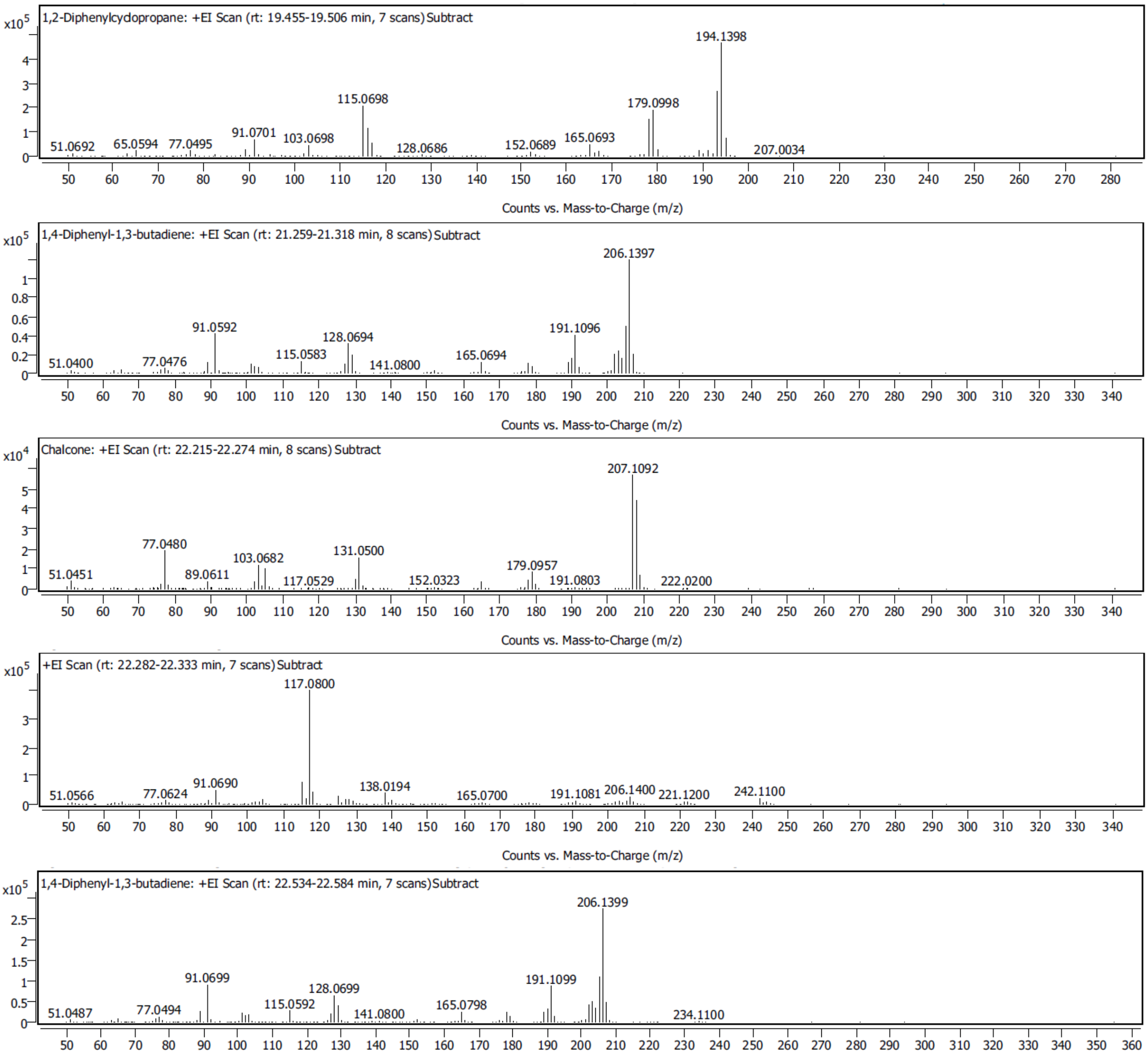

Figure S36. EI-mass spectra of the GC peaks of the filtered reaction mixture of **1** with 1 equivalent of benzaldehyde in THFd$_8$ at ambient temperature for 6 d from 19.455 min to 22.584 min, evaluated by *Agilent* MassHunter.

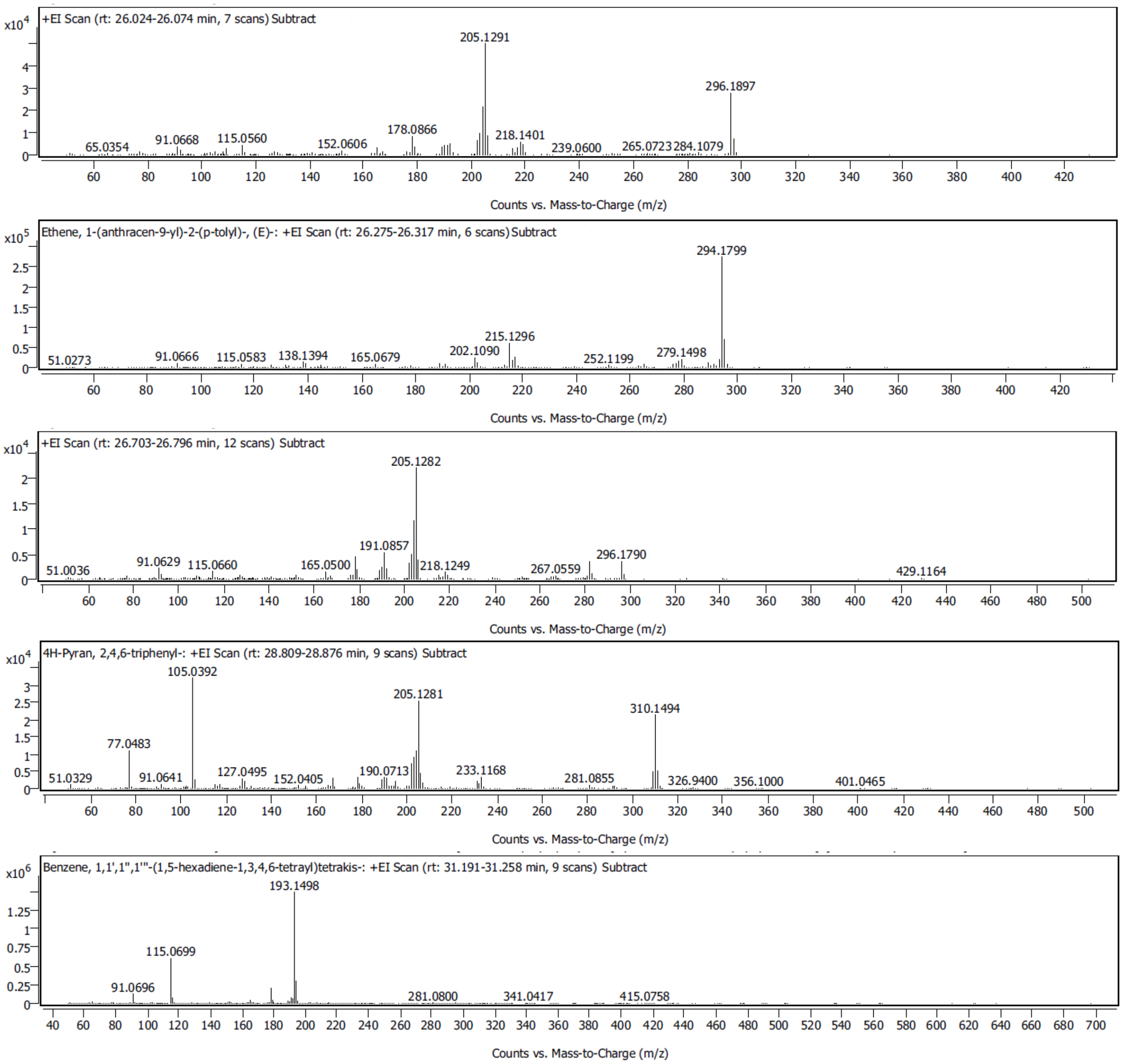

Figure S37. EI-mass spectra of the GC peaks of the filtered reaction mixture of **1** with 1 equivalent of benzaldehyde in THF-d_8 at ambient temperature for 6 d from 26.024 min to 31.258 min, evaluated by *Agilent* MassHunter.

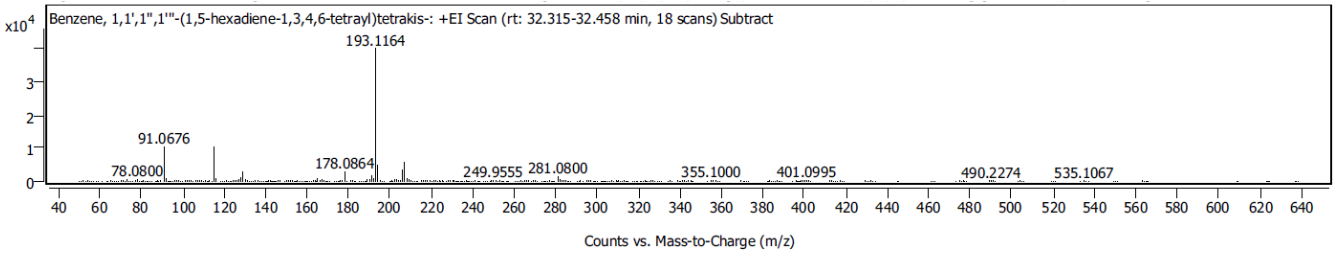

Figure S38. EI-mass spectra of the GC peaks of the filtered reaction mixture of **1** with 1 equivalent of benzaldehyde in THF-d_8 at ambient temperature for 6 d at 32.315-32.458 min, evaluated by *Agilent* MassHunter.

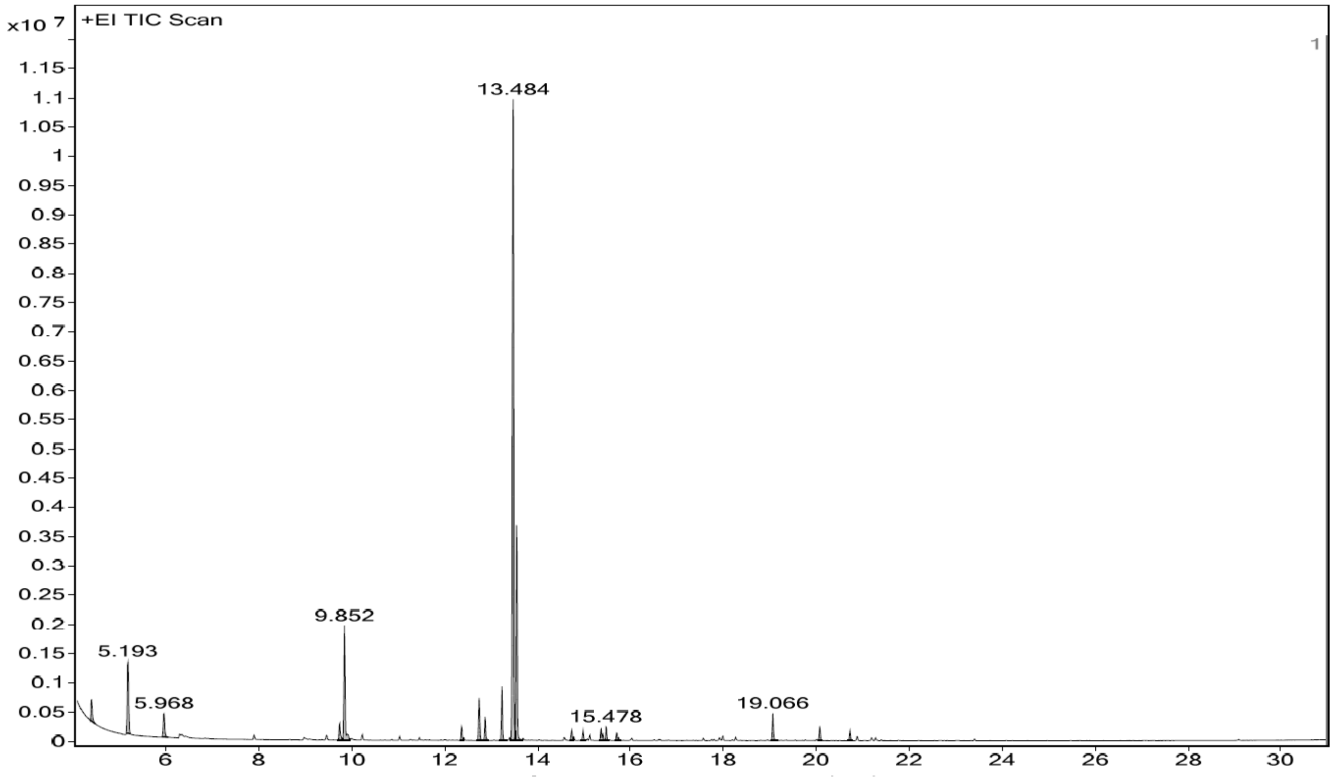

Figure S39. Gas chromatogram of the filtered reaction mixture of **1** with 1 equivalent of pivalaldehyde in THF-d_8 at ambient temperature for 3 d.

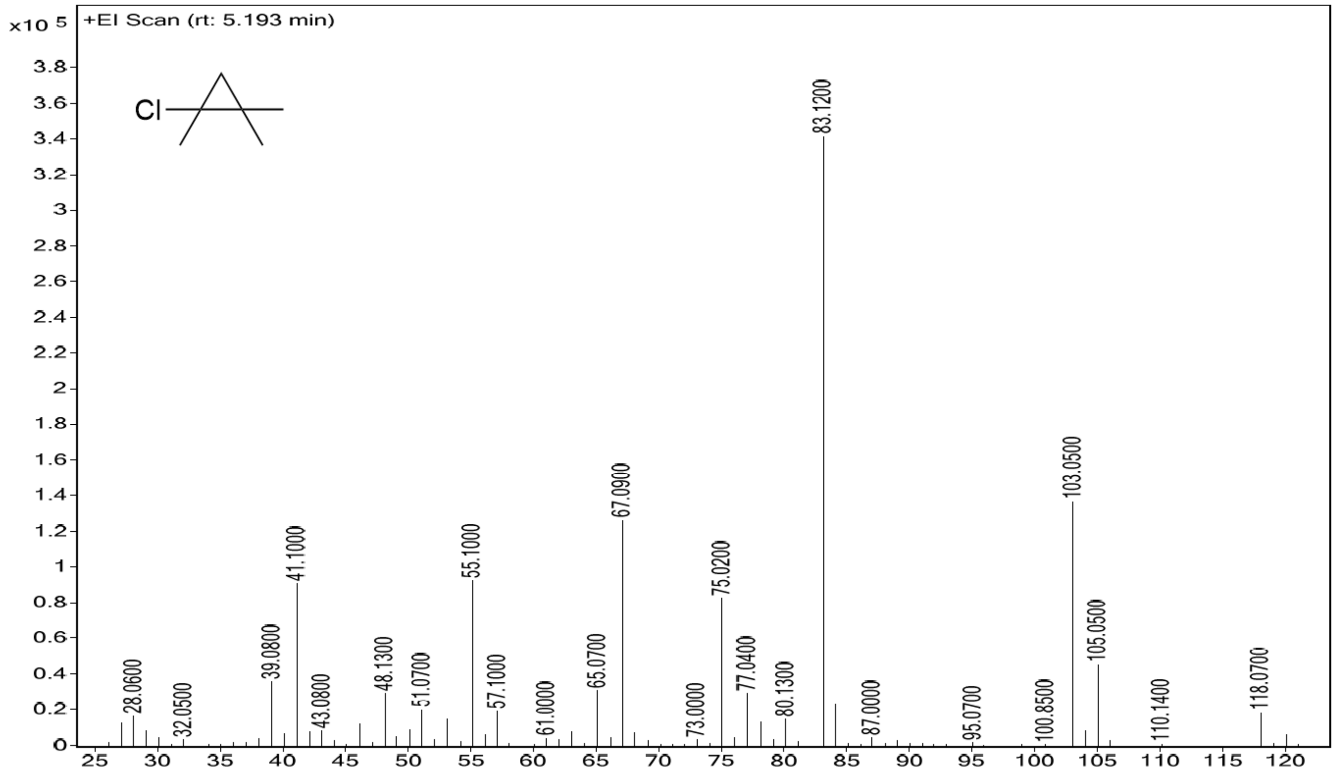

Figure S40. Mass spectrum (EI) of the GC peak of the filtered reaction mixture of **1** with 1 equivalent of pivalaldehyde in THF-d_8 at ambient temperature for 3 d at 5.193 min, suggested compound (mainlib), 1-chloro-1,2,2-trimethylcyclopropane.

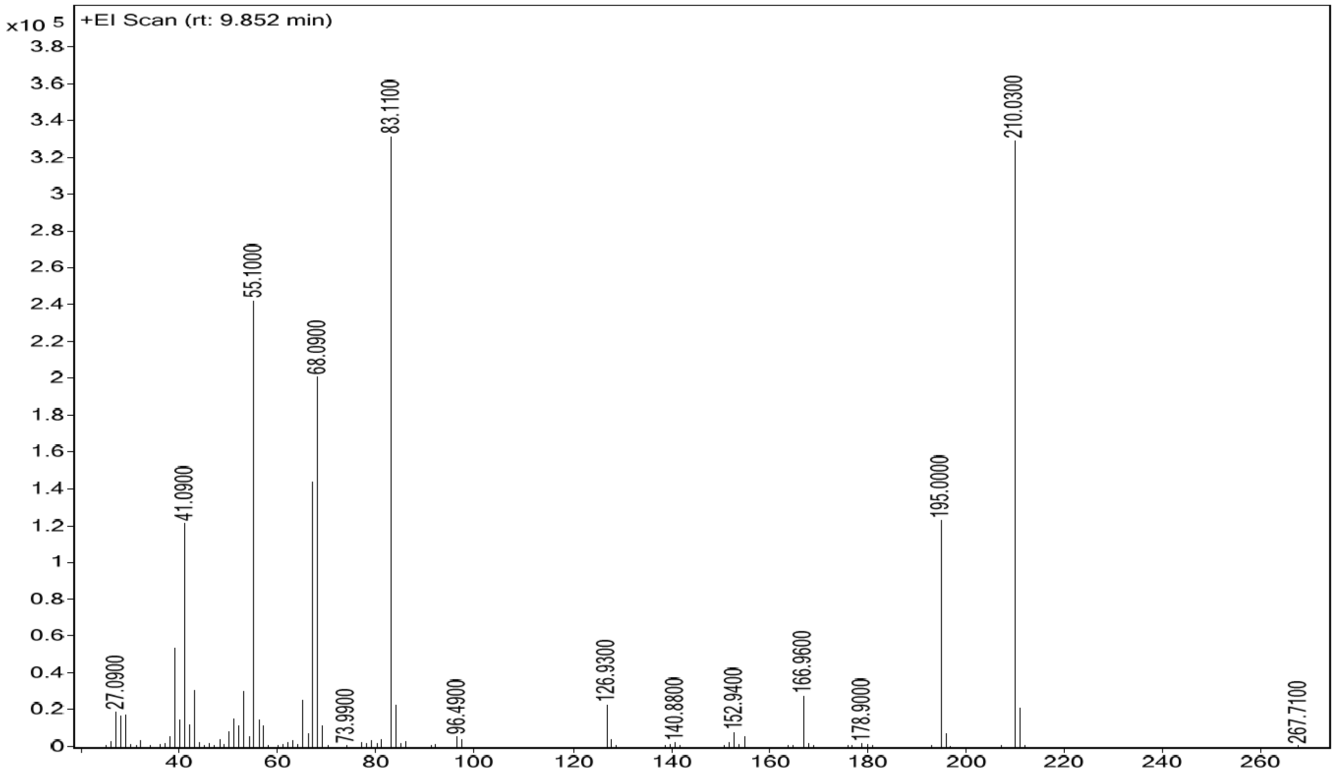

Figure S41. Mass spectrum (EI) of the GC peak of the filtered reaction mixture of **1** with 1 equivalent of pivalaldehyde in THF-d_8 at ambient temperature for 3 d at 9.852 min.

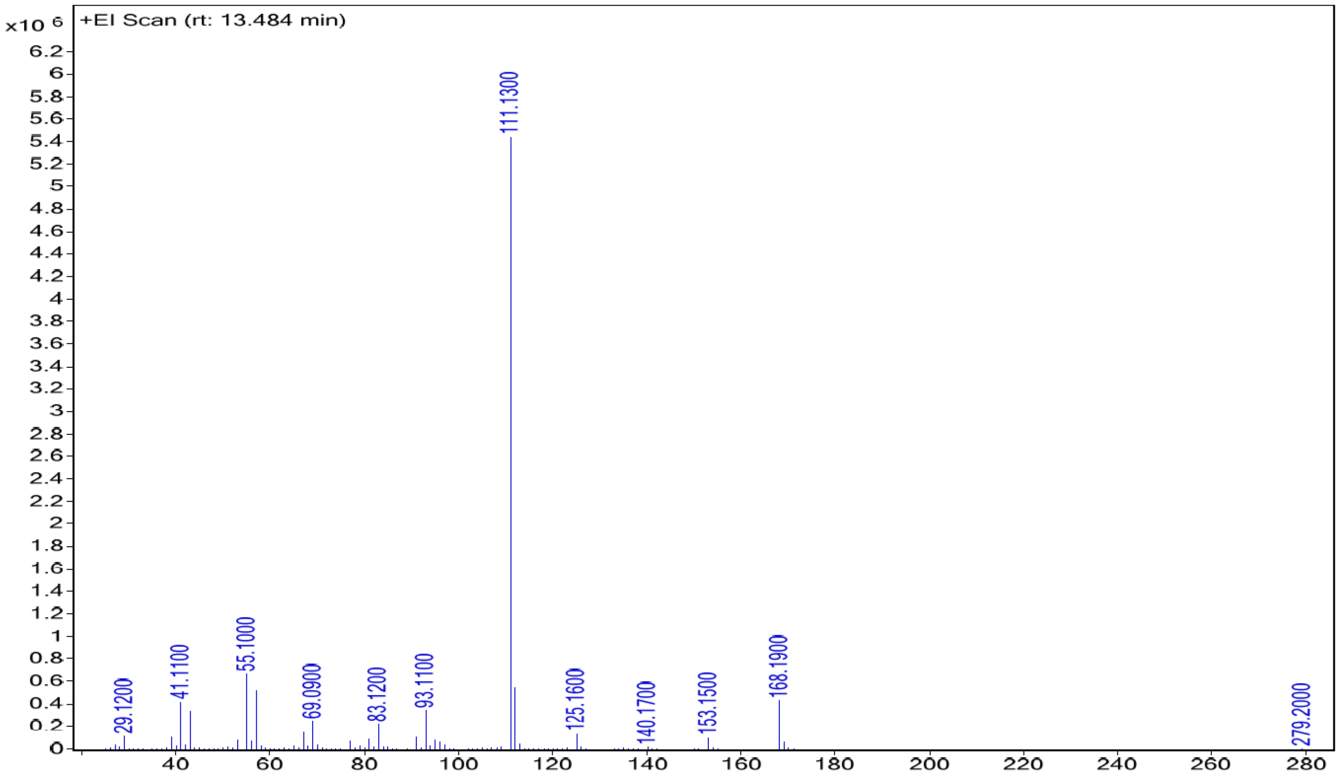

Figure S42. Mass spectrum (EI) of the GC peak of the filtered reaction mixture of **1** with 1 equivalent of pivalaldehyde in THF-d_8 at ambient temperature for 3 d at 13.484 min.

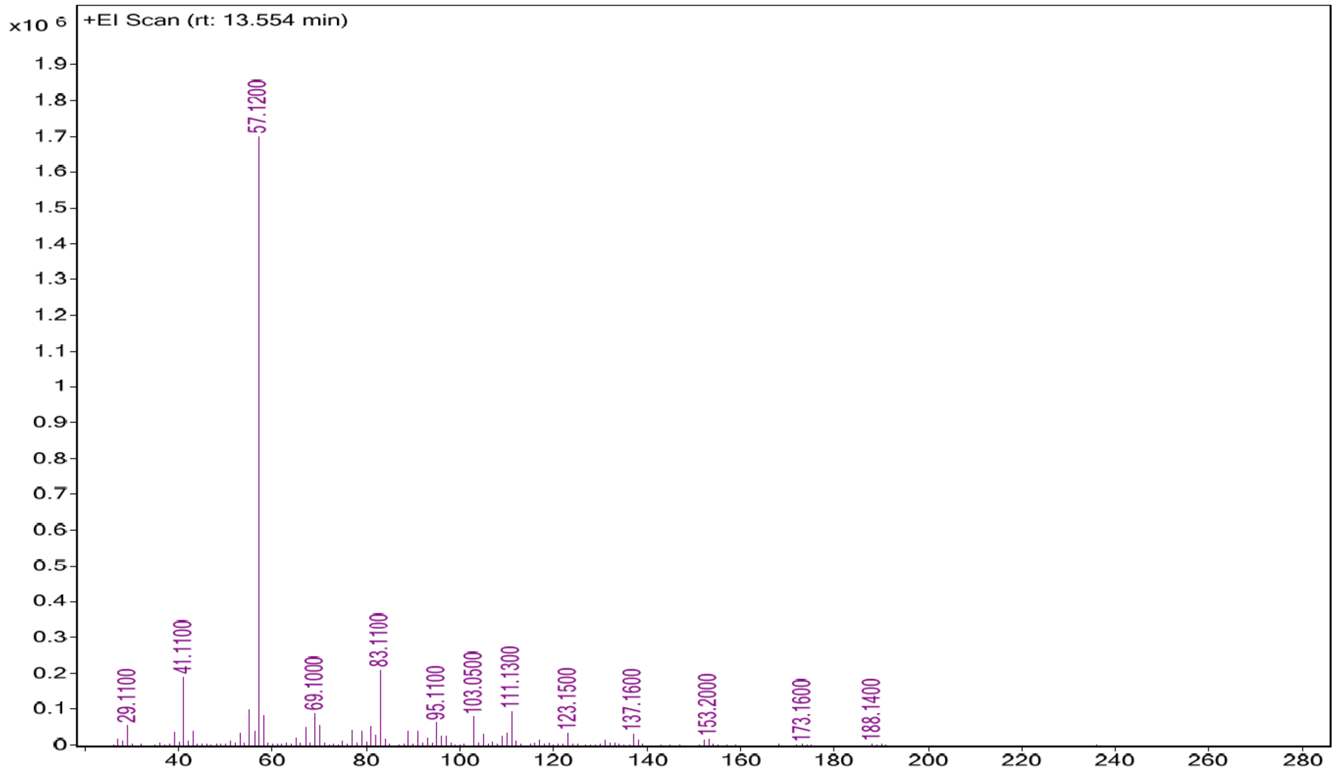

Figure S43. Mass spectrum (EI) of the GC peak of the filtered reaction mixture of **1** with 1 equivalent of pivalaldehyde in THF-d_8 at ambient temperature for 3 d at 13.554 min.

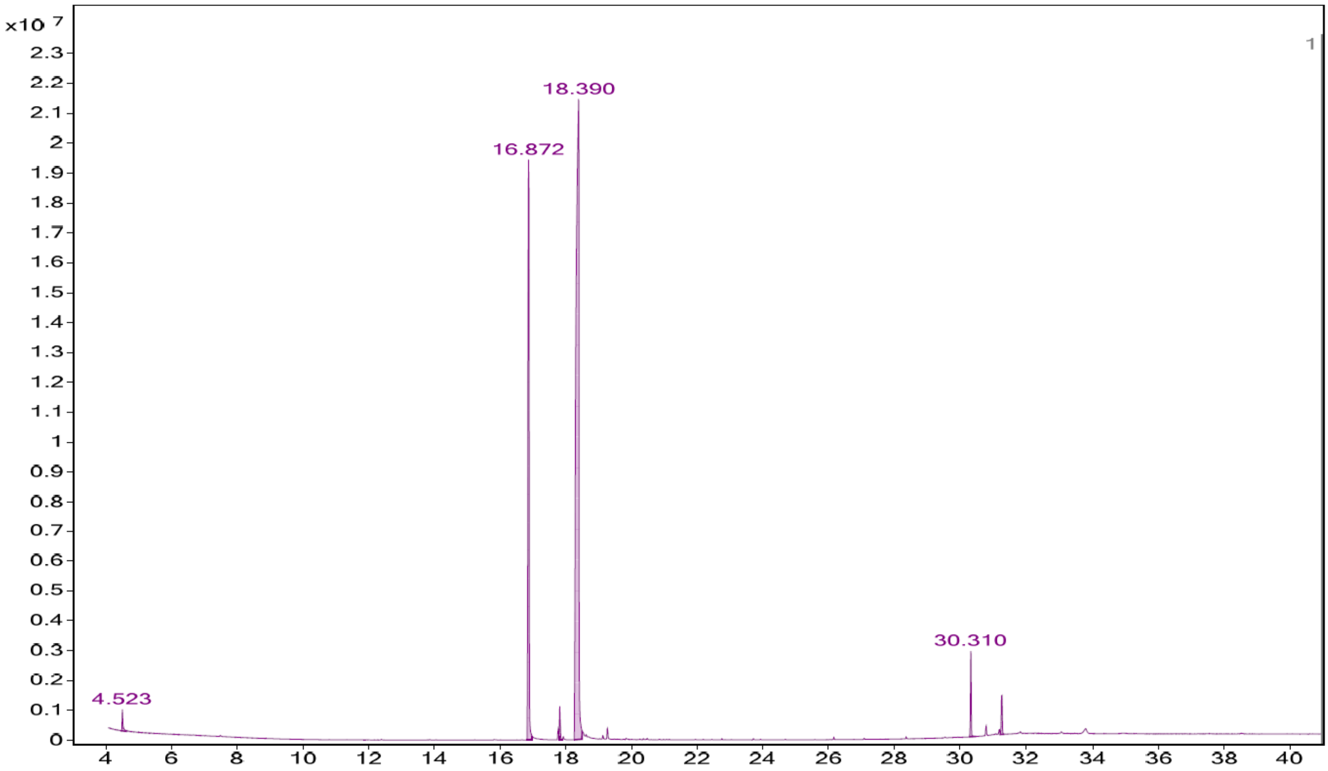

Figure S44. Gas chromatogram of the filtered reaction mixture of **1** with 1 equivalent of benzophenone in THF-d_8 at ambient temperature for 3 d.

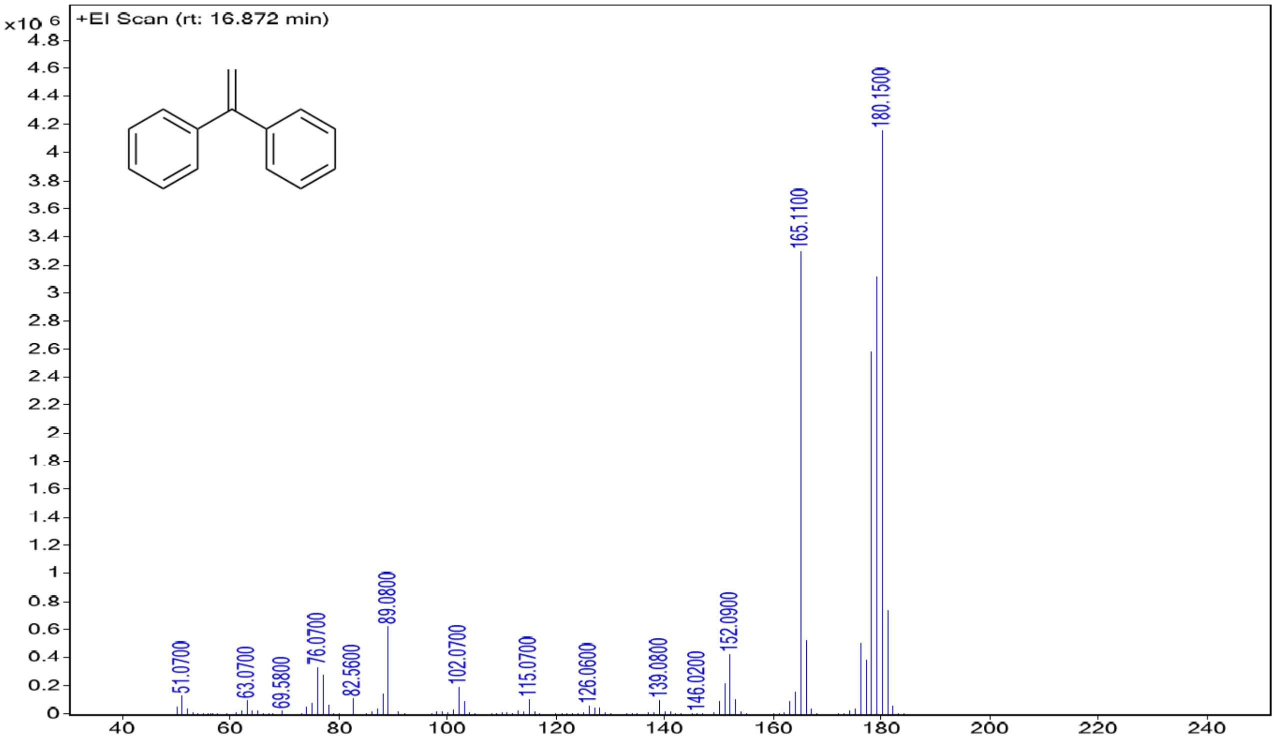

Figure S45. Mass spectrum of the GC peak of the filtered reaction mixture of **1** with 1 equivalent of benzophenone in THF-d_8 at ambient temperature for 3 d at 16.872 min, suggested compound (mainlib) 1,1-diphenylethylene.

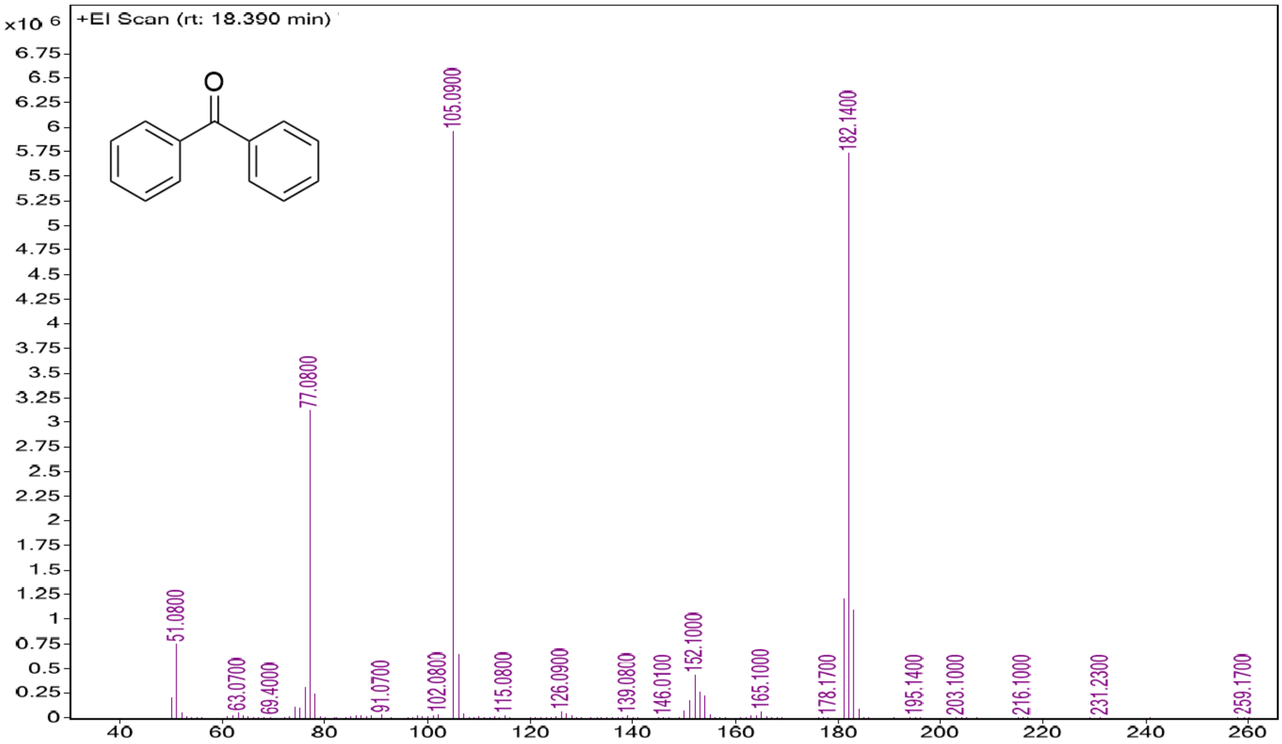

Figure S46. Mass spectrum of the GC peak of the filtered reaction mixture of **1** with 1 equivalent of benzophenone in THF-d_8 at ambient temperature for 3 d at 18.390 min, suggested compound (mainlib) benzophenone.

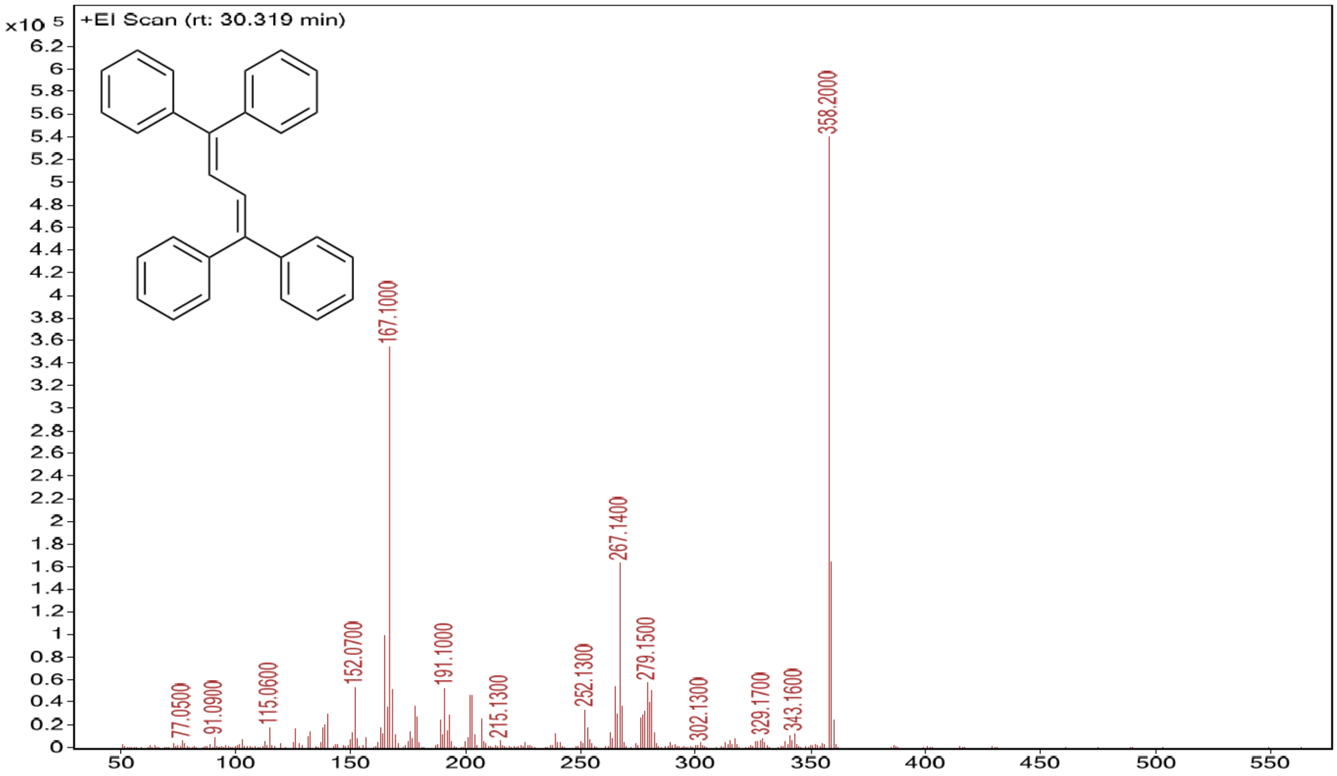

Figure S47. Mass spectrum of the GC peak of the filtered reaction mixture of (**1**) with 1 equivalent of benzophenone inTHF-d_8 at ambient temperature for 3 d at 30.319 min, suggested compound (mainlib) 1,1',1'',1'''-(1,3-butadiene-1,4-diylidene)tetrakis(benzene).

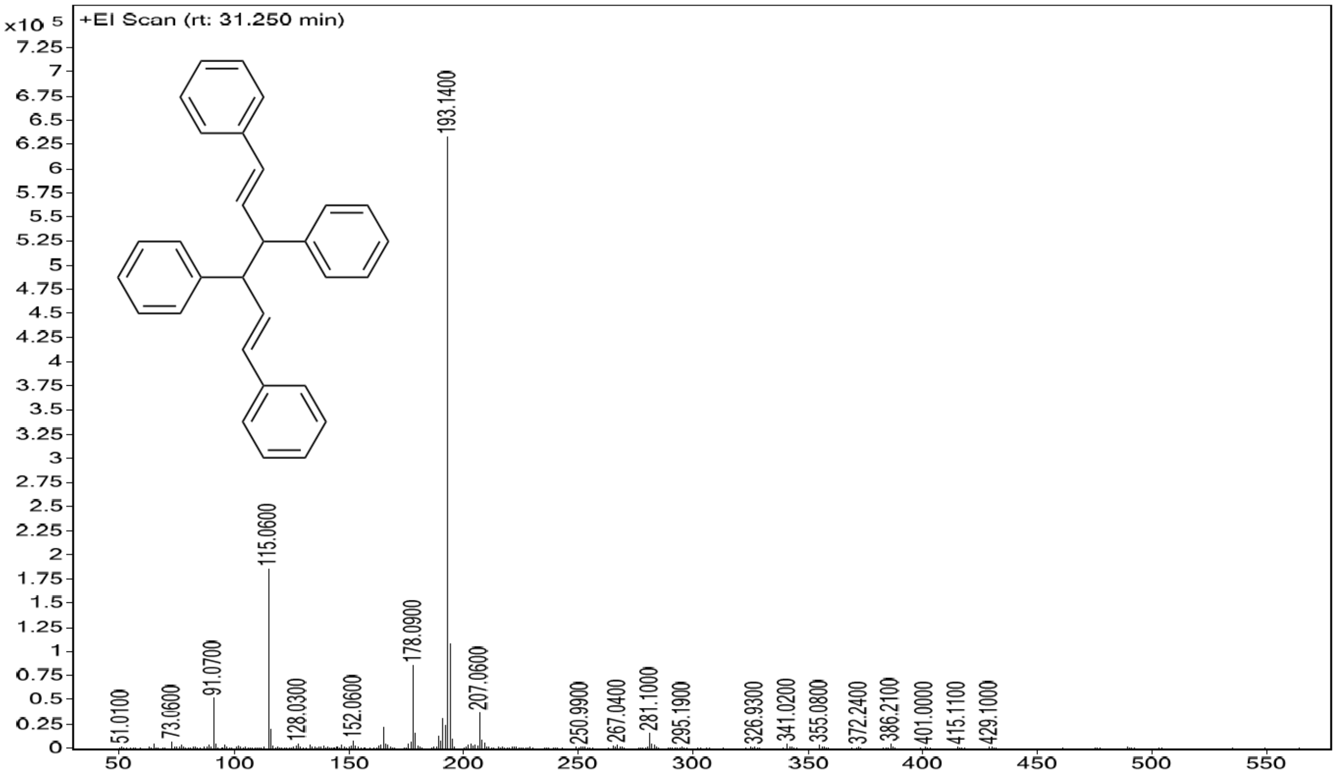

Figure S48. Mass spectrum of the GC peak of the filtered reaction mixture of (**1**) with 1 equivalent of benzophenone in THF-d_8 at ambient temperature for 3 d at 31.250 min, suggested compound (mainlib) 1,1'1'',1'''-(1,5-hexadiene-1,3,4,6-tetrayl)tetrakis(benzene).

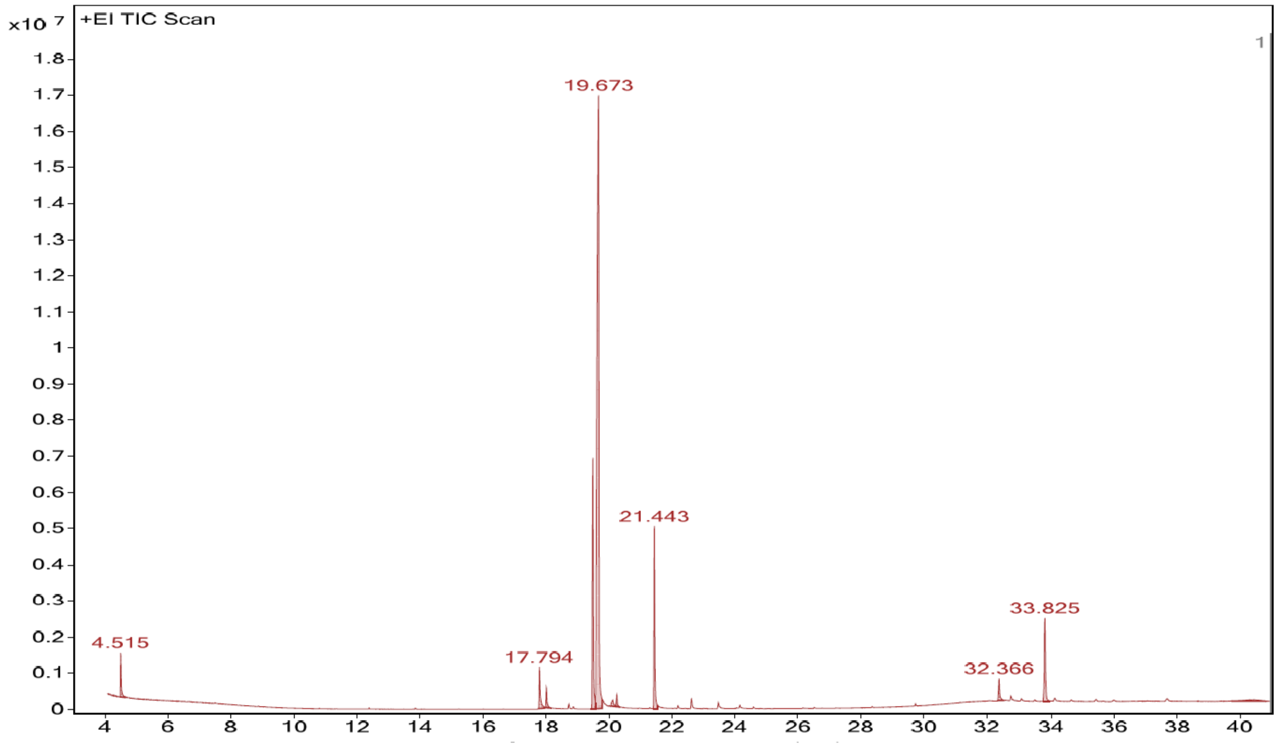

Figure S49. Gas chromatogram of the filtered reaction mixture of **1** with 1 equivalent of 9-fluorenone in THF-d_8 at ambient temperature for 3 d.

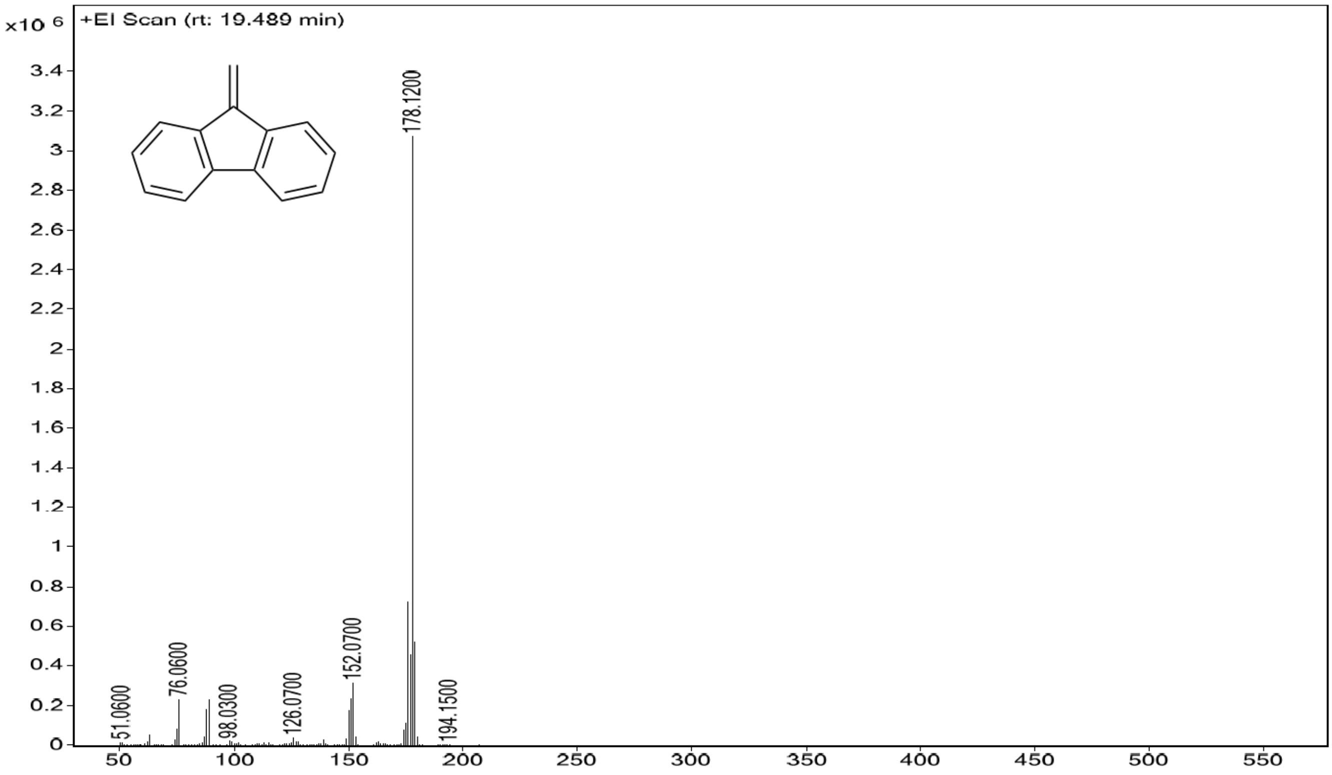

Figure S50. Mass spectrum (EI) of the GC peak of the filtered reaction mixture of **1** with 1 equivalent of 9-fluorenone in THF-d_8 at ambient temperature for 3 d at 19.489 min, suggested compound (mainlib) 9-methylene-9H-fluorene.

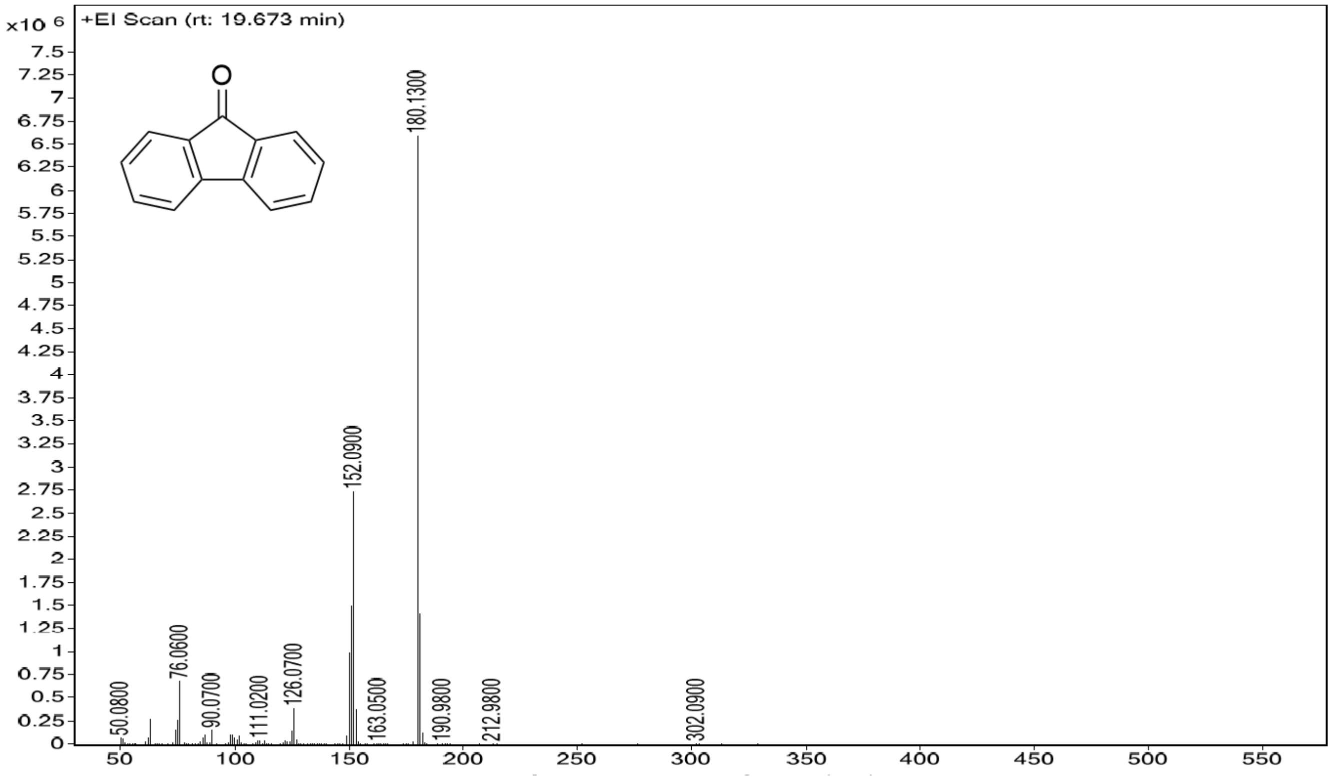

Figure S51. Mass spectrum (EI) of the GC peak of the filtered reaction mixture of **1** with 1 equivalent of 9-fluorenone in THF-d_8 at ambient temperature for 3 d at 19.673 min, suggested compound (mainlib) 9H-fluoren-9-one.

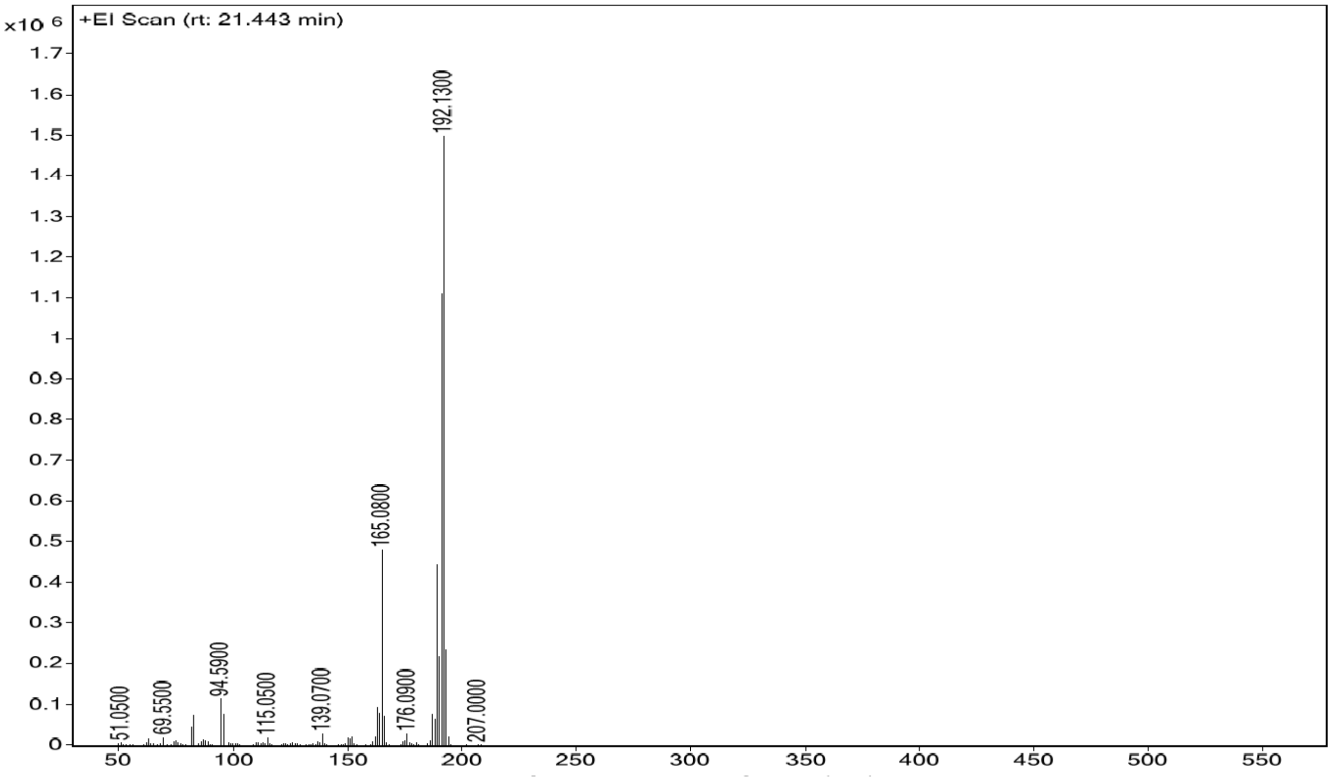

Figure S52. Mass spectrum (EI) of the GC peak of the filtered reaction mixture of **1** with 1 equivalent of 9-fluorenone in THF-d_8 at ambient temperature for 3 d at 21.443 min.

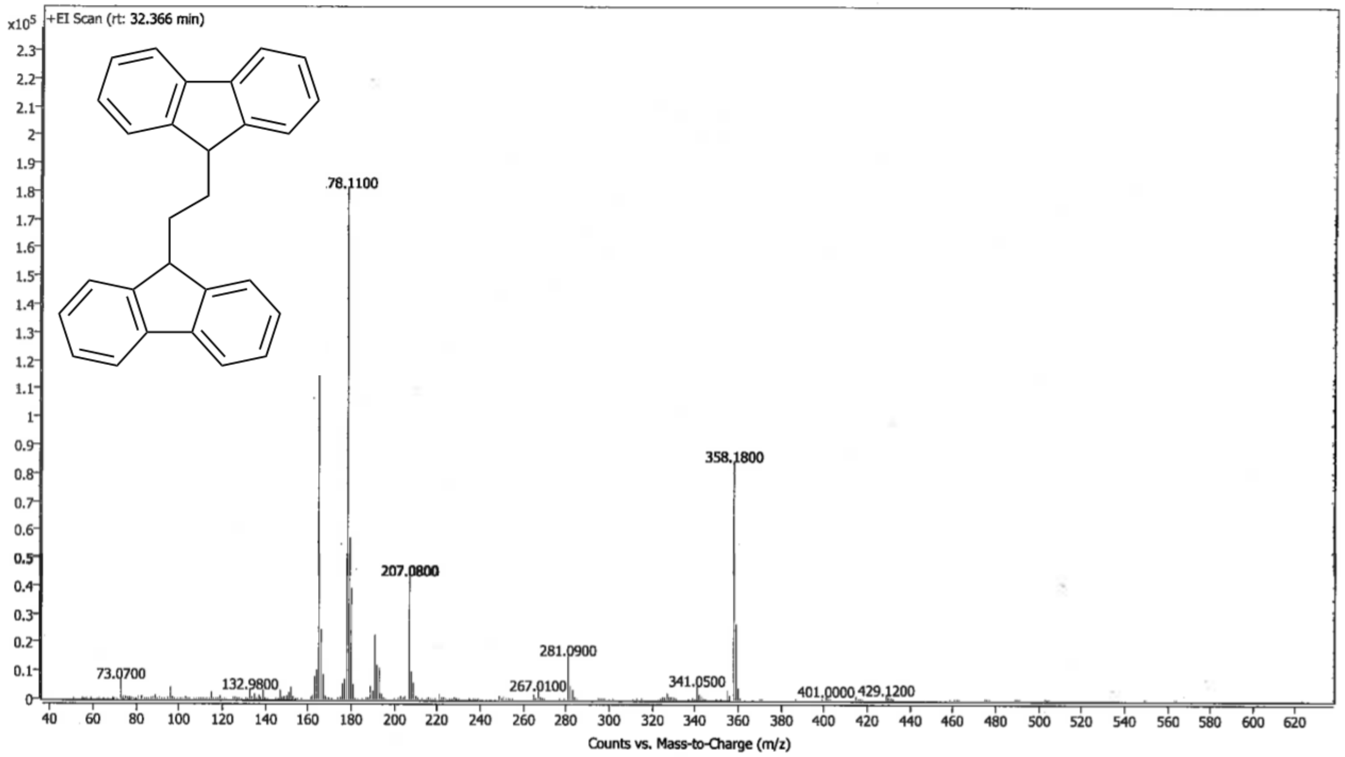

Figure S53. Mass spectrum (EI) of the GC peak of the filtered reaction mixture of **1** with 1 equivalent of 9-fluorenone in THF-d_8 at ambient temperature for 3 d at 32.366 min, suggested compound (mainlib) 1,2-di(9-fluorenyl)ethane.

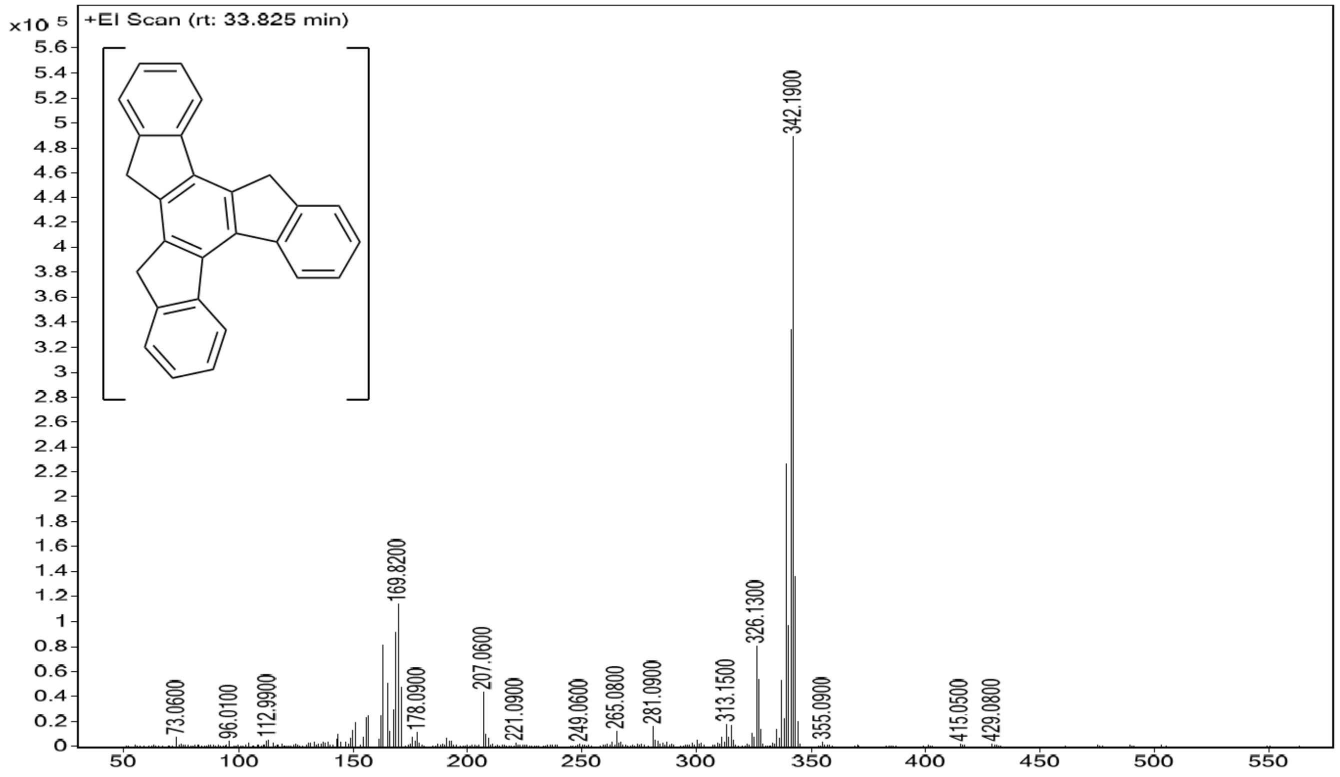

Figure S54. Mass spectrum (EI) of the GC peak of the filtered reaction mixture of **1** with 1 equivalent of 9-fluorenone in THF-d_8 at ambient temperature for 3 d at 33.825 min, suggested compound (mainlib) 3H,3'H,3''H-trisindeno[1,2-a:2',1'-c:1'',2''-e]benzene.

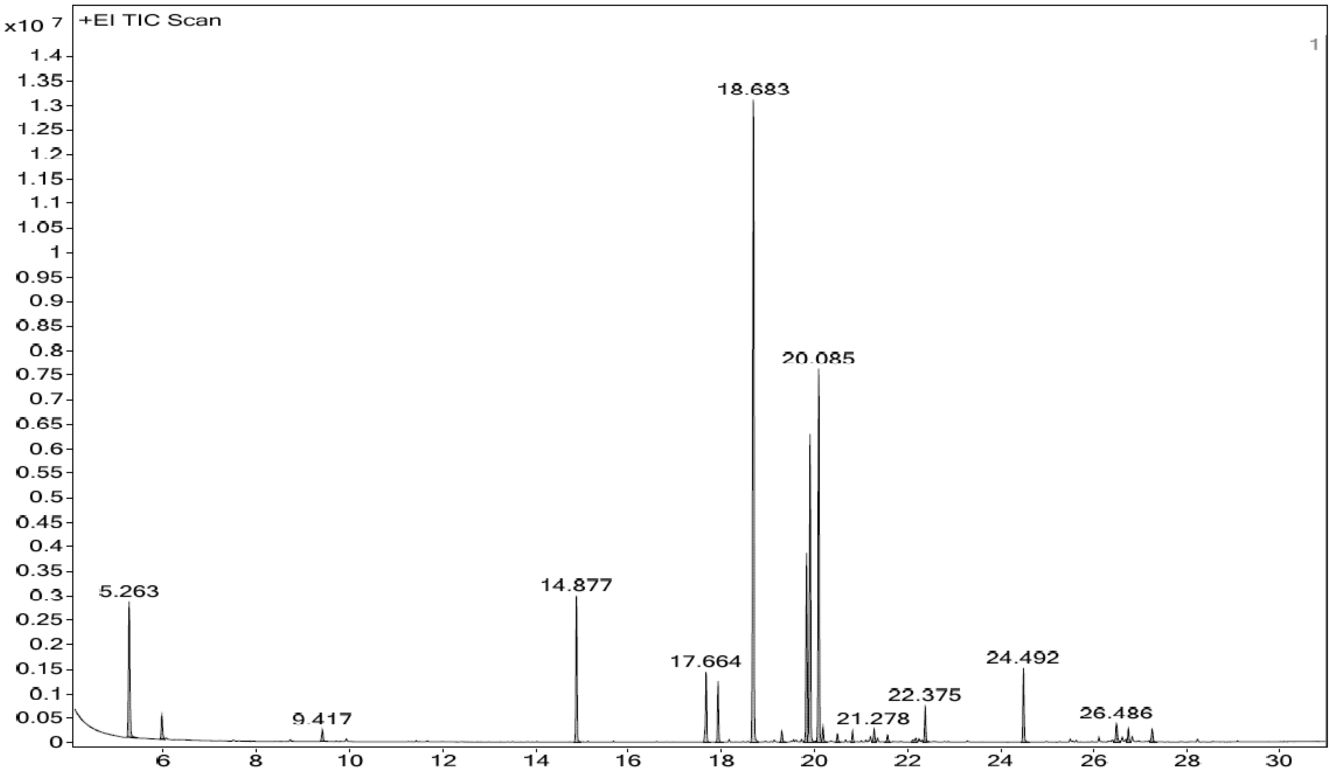

Figure S55. Gas chromatogram of the reaction of **1** with 1 equivalent of cyclohexanone in THF-d_8 at ambient temperature for 3 d.

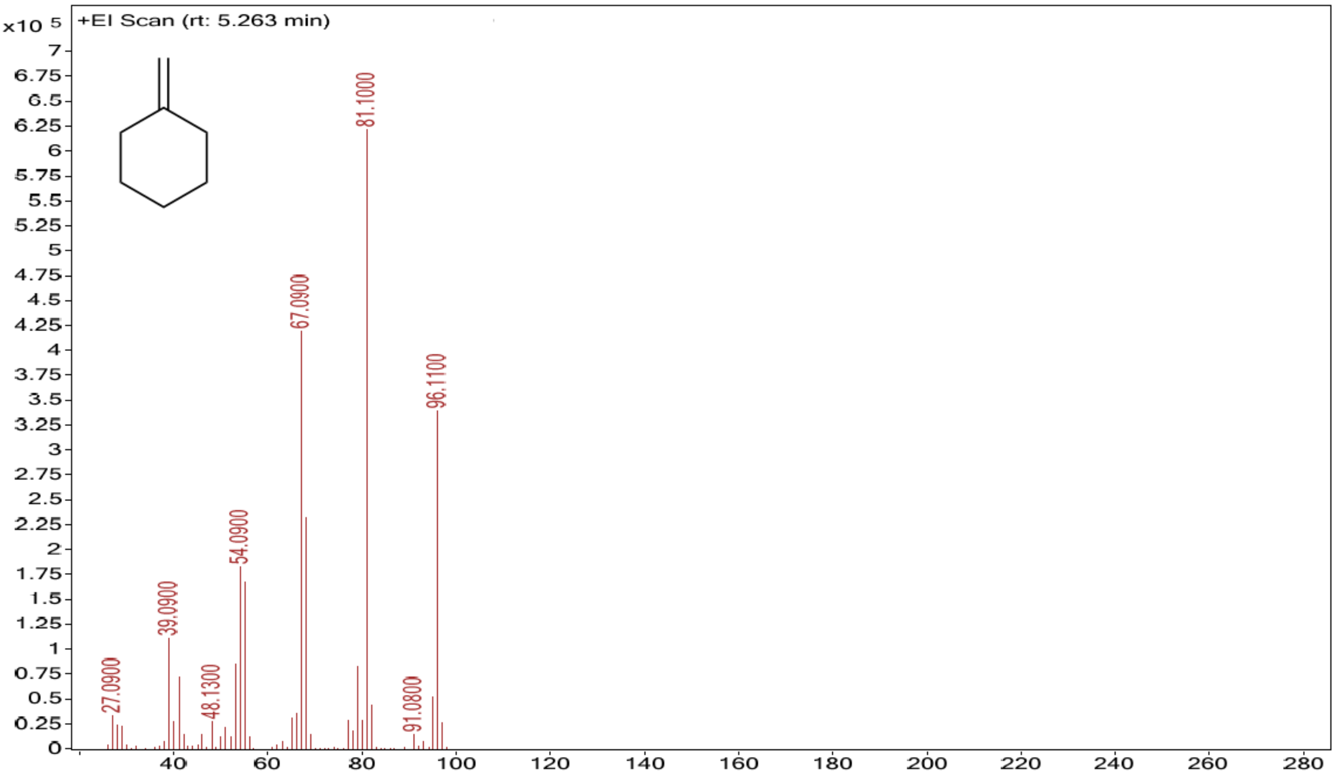

Figure S56. Mass spectrum (EI) of the GC peak of the reaction of **1** with 1 equivalent of cyclohexanone in THF-d_8 at ambient temperature for 3 d at 5.263 min, suggested compound (mainlib) methylene-cyclohexane.

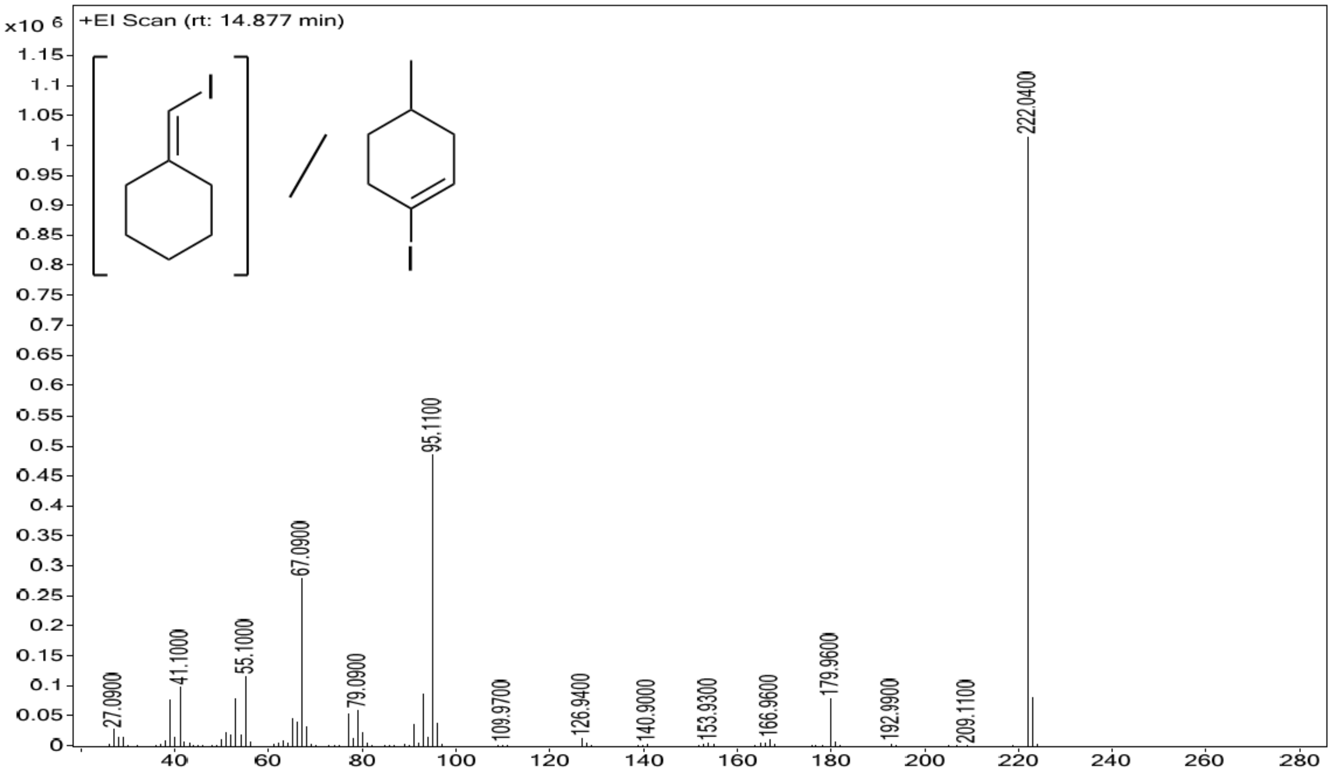

Figure S57. Mass spectrum (EI) of the GC peak of teh reaction of **1** with 1 equivalent of cyclohexanone in THF-d_8 at ambient temperature for 3 d at 14.877 min, suggested compound Iodomethylene-cyclohexane / (mainlib) 1-iodo-4-methyl-cyclohex-1-ene.

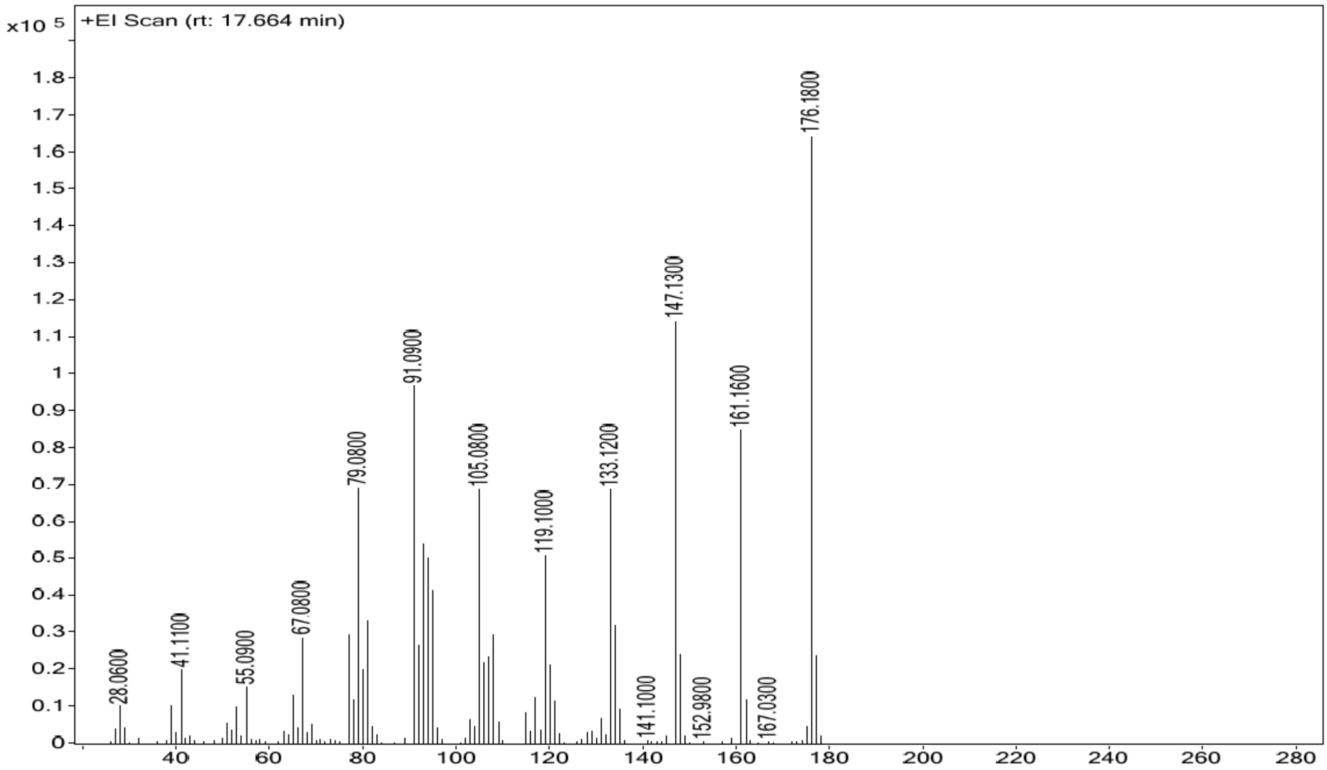

Figure S58. Mass spectrum (EI) of the GC peak of the reaction of **1** with 1 equivalent of cyclohexanone in THF-d_8 at ambient temperature for 3 d at 17.664 min.

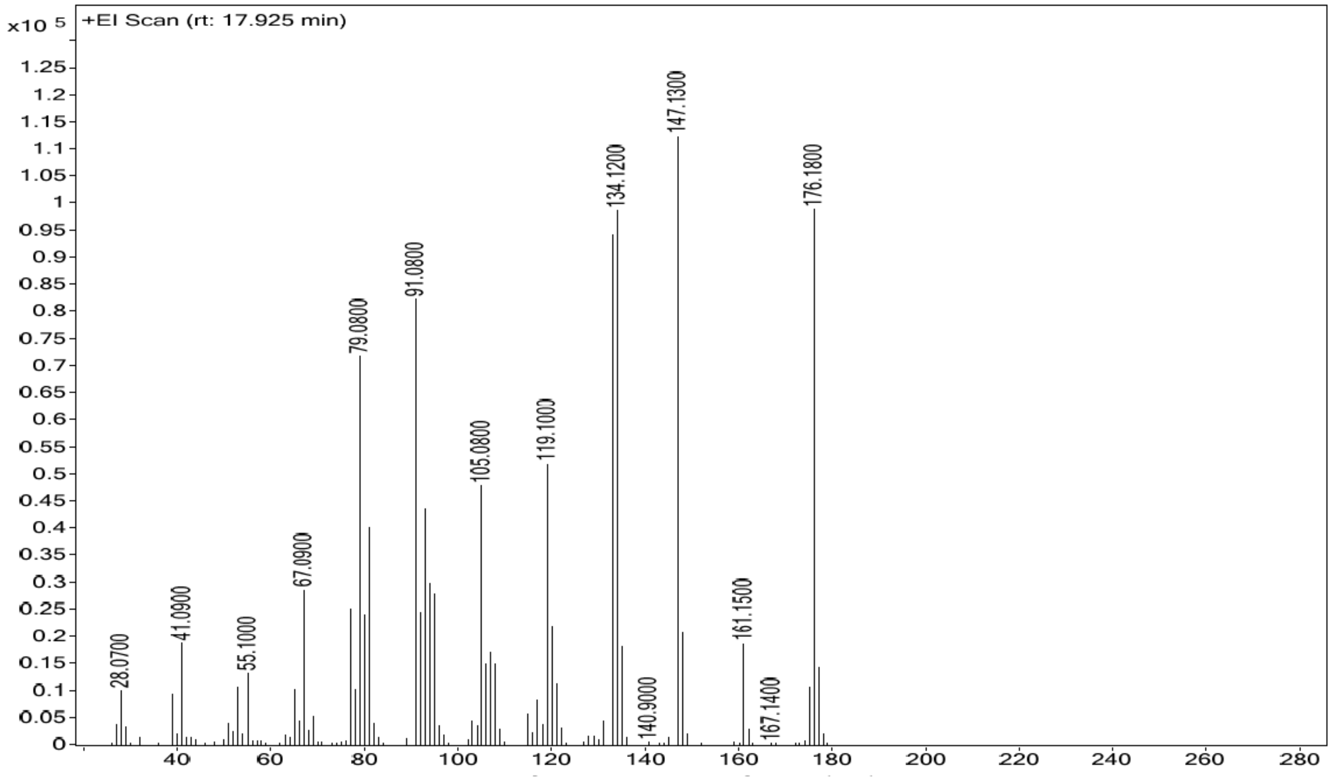

Figure S59. Mass spectrum (EI) of the GC peak of the reaction of **1** with 1 equivalent of cyclohexanone in THF-d_8 at ambient temperature for 3 d at 17.925 min.

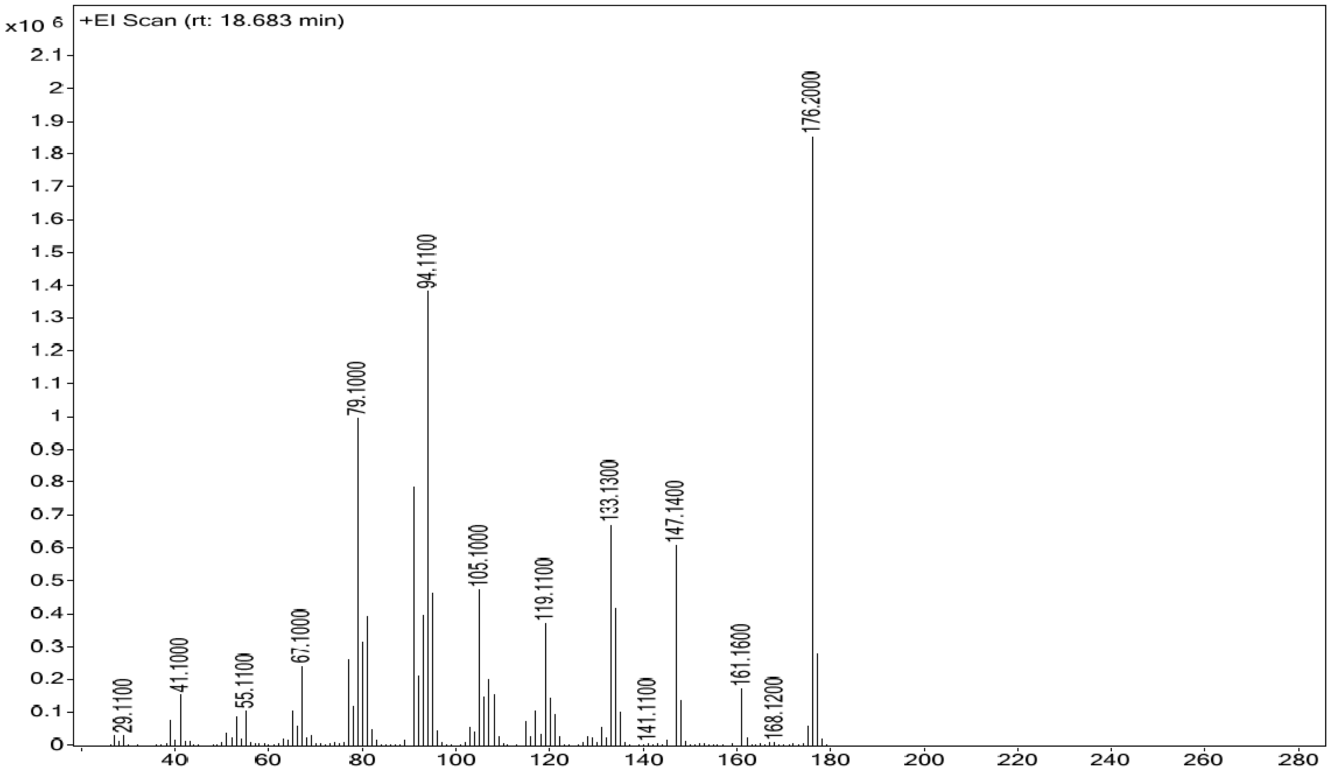

Figure S60. Mass spectrum (EI) of the GC peak of the reaction of **1** with 1 equivalent of cyclohexanone in THF-d_8 at ambient temperature for 3 d at 18.683 min.

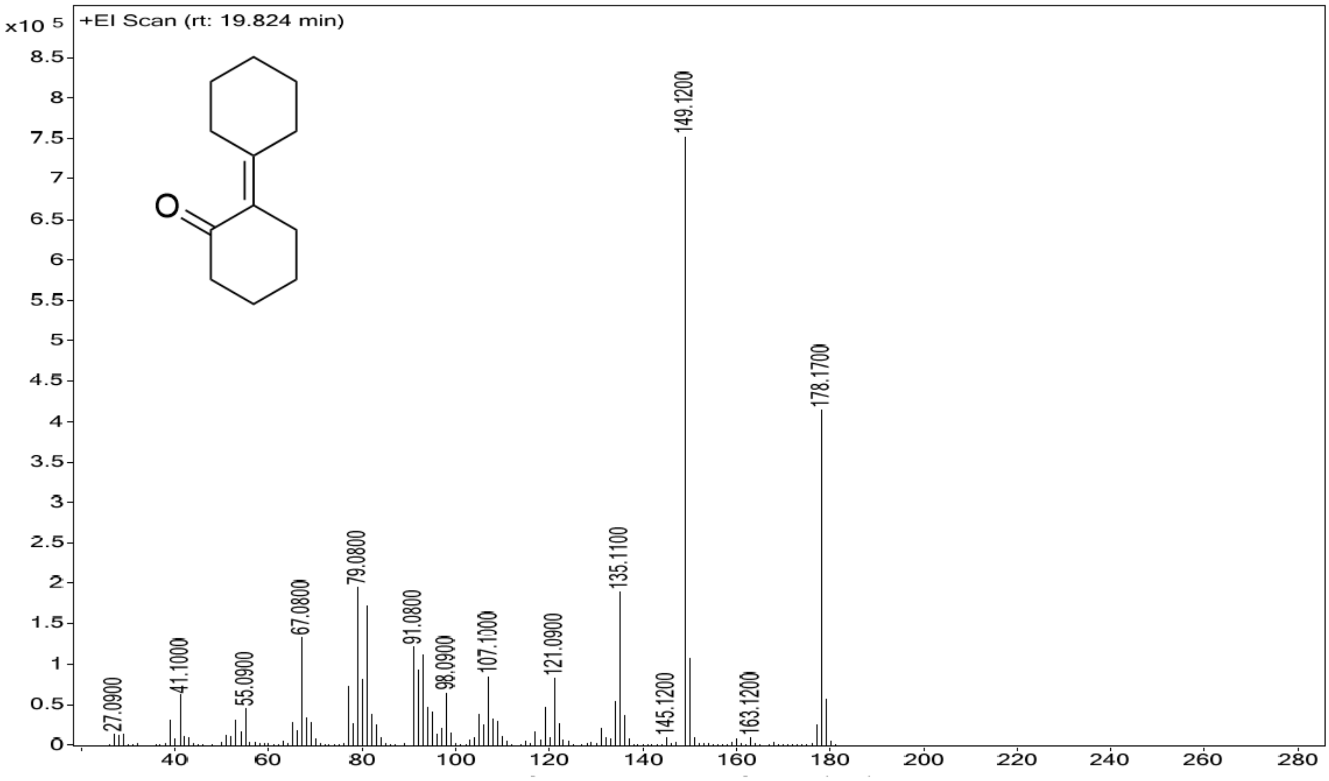

Figure S61. Mass spectrum (EI) of the GC peak of the reaction of **1** with 1 equivalent of cyclohexanone in THF-d_8 at ambient temperature for 3 d at 19.824 min, suggested compound (mainlib) 2-cyclohexylidene-cyclohexanone.

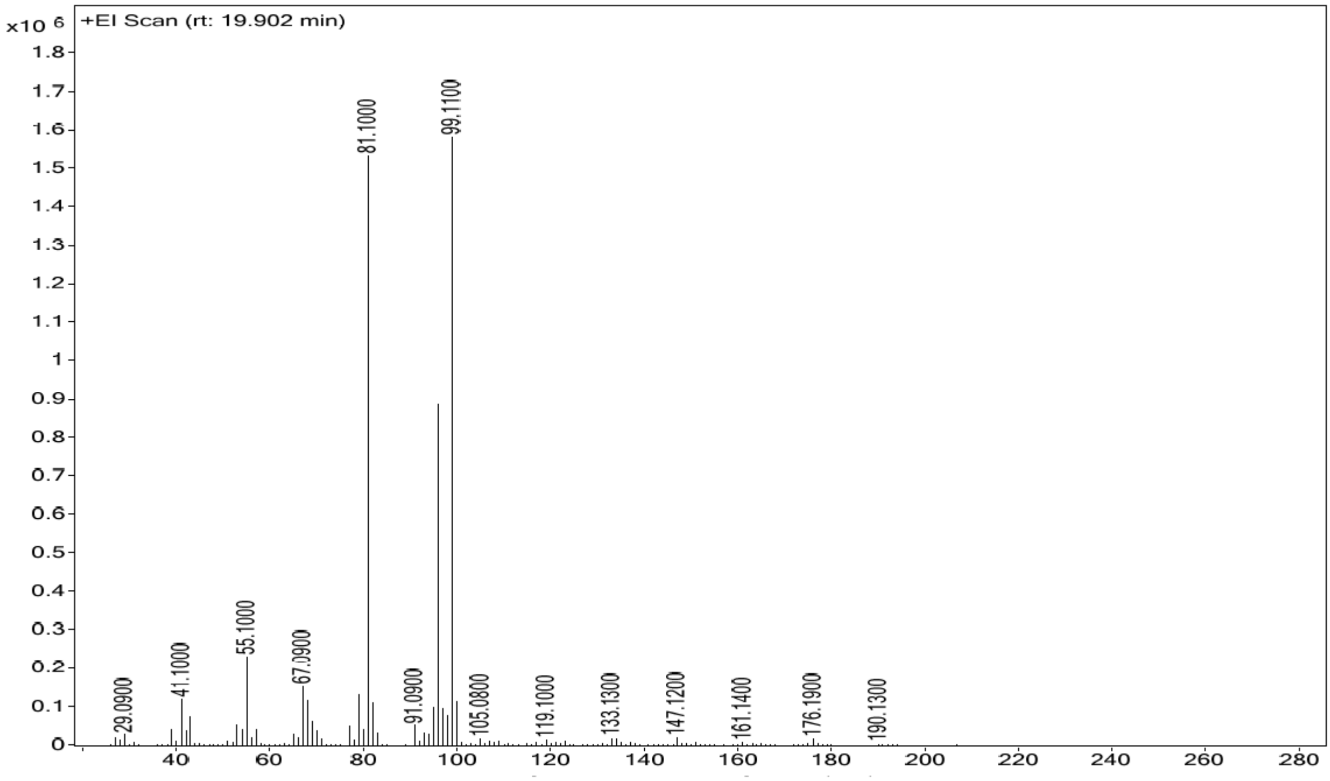

Figure S62. Mass spectrum (EI) of the GC peak of the reaction of **1** with 1 equivalent of cyclohexanone in THF-d_8 at ambient temperature for 3 d at 19.902 min.

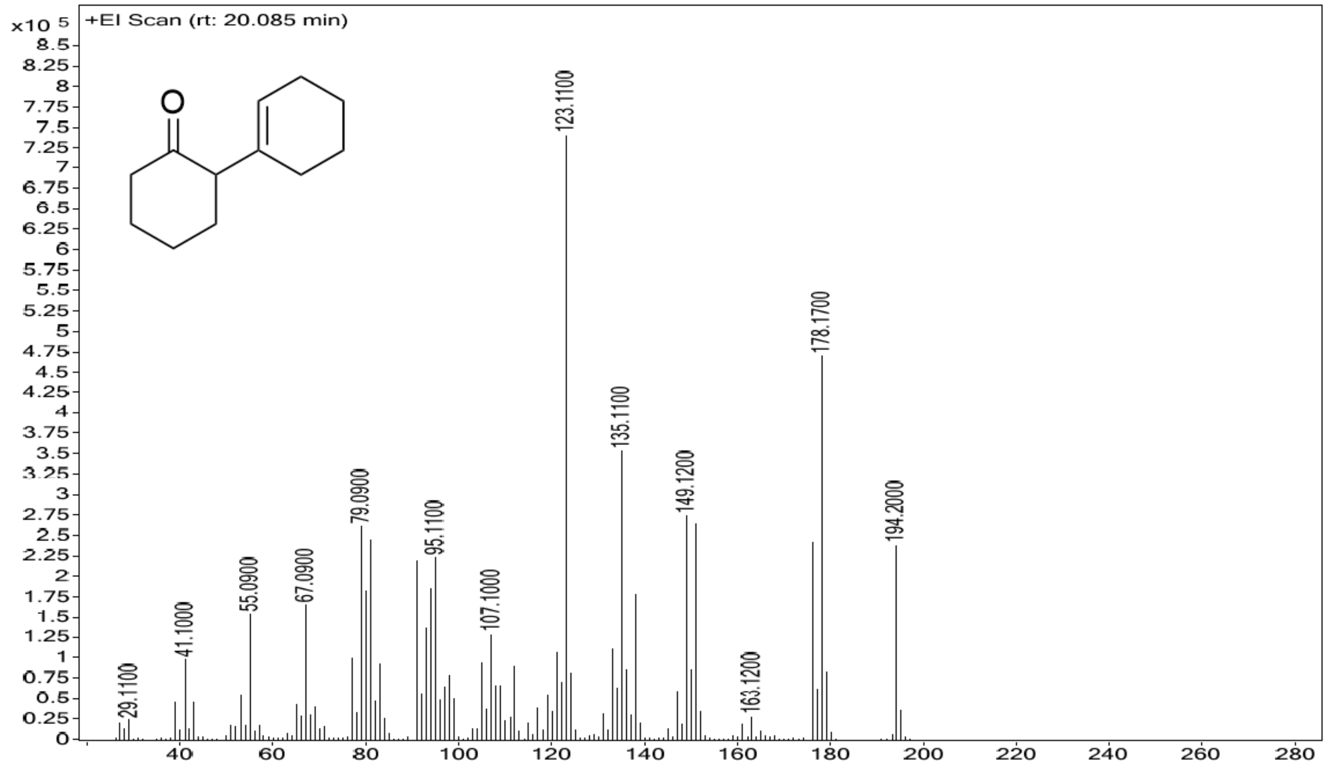

Figure S63. Mass spectrum (EI) of the GC peak of the reaction of **1** with 1 equivalent of cyclohexanone in THF-d8 at ambient temperature for 3 d at 20.085 min, suggested compound (mainlib) 2-(1-cyclohexen-1-yl)-cyclohexanone.

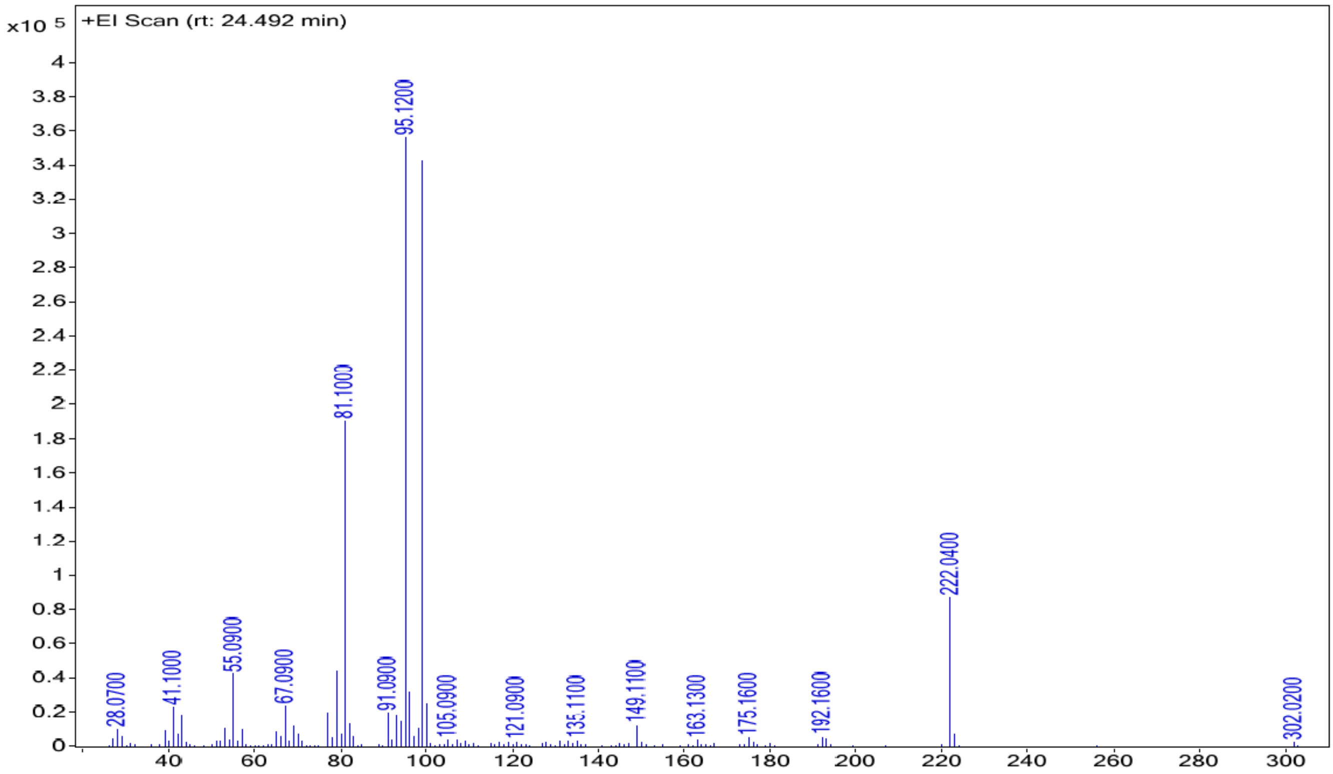

Figure S64. Mass spectrum (EI) of the GC peak of the reaction of **1** with 1 equivalent of cyclohexanone in THF-d8 at ambient temperature for 3 d Peak at 24.492 min.

References

[1] R. A. Heintz, R. L. Ostrander, A. L. Rheingold, K. H. Theopold, *J. Am. Chem. Soc.* **1994**, *116*, 11387-11396.

[2] G. R. Fulmer, A. J. M. Miller, N. H. Sherden, H. E. Gottlieb, A. Nudelman, B. M. Stoltz, J. E. Bercaw, K. I. Goldberg, *Organometallics* **2010**, *29*, 2176–2179.

[3] a) D. F. Evans *J. Chem. Soc.*, **1959**, 2003-2005; b) E. M. Schubert *J. Chem. Educ.*, **1992**, *69*, 62.

[4] Agilent MassHunter WorkStation - Qualitative Analysis for GC/MS (RRID:SCR_016657).

[5] P. Linstrom, *NIST Chemistry WebBook, NIST Standard Reference Database 69*, National Institute of Standards and Technology, **1997**.

[6] COSMO, v. 1.61; Bruker AXS Inc., Madison, WI, 2012.

[7] APEX 3, v. 2016.5-0; Bruker AXS Inc., Madison, WI, 2012.

[8] SAINT, v. 8.34A; Bruker AXS Inc., Madison, WI, 2010.

[9] L. Krause, R. Herbst-Irmer, G. M. Sheldrick, D. Stalke*, J. Appl. Cryst*. **2015**, *48*, 3-10.

[10] G. M. Sheldrick, *(2009), TWINABS,* University of Göttingen, Germany.

[11] G. M. Sheldrick, *Acta Crystallogr*., Sect. A **2015**, *71*, 3-8.

[12] C. B. Hübschle, G. M. Sheldrick, B. J. Dittrich, *J. Appl. Cryst*. **2011**, *44*, 1281-1284.

[13] D. Kratzert, J. J. Holstein, I. Krossing, DSR: enhanced modelling and refinement of disordered structures with SHELXL. *J. Appl. Cryst.* **2015**, *48*, 933-938.

[14] C. F. Macrae, I. J. Bruno, J. A. Chisholm, P. R. Edgington, P. McCabe, E. Pidcock, L. RodriguezMonge, R. Taylor, J. van de Streek, P. A. Wood, *J. Appl. Cryst*. **2008**, *41*, 466-470.

[15] G. A. Bain, J. F. Berry, *J. Chem. Educ.* **2008**, *85*, 532-536.
